# Genome-wide CRISPR screen identifies a cytokine-enhancer circuit driving HIF-2**α** activation in renal cancer

**DOI:** 10.1172/JCI201639

**Published:** 2026-03-24

**Authors:** Jun Fang, Jeremy M. Simon, Tao Wang, Yunpeng Gao, Xianju Bi, Lianxin Hu, Chengheng Liao, Cheng Zhang, Yayoi Adachi, Jin Zhou, Hongyi Liu, Qian Liang, James A. Nathan, Ram Mani, James Brugarolas, Qing Zhang

**Affiliations:** 1 Department of Pathology, University of Texas Southwestern Medical Center, Dallas, Texas, USA.; 2 Department of Data Science, Dana-Farber Cancer Institute, Boston, Massachusetts, USA.; 3 Department of Biostatistics, Harvard T.H. Chan School of Public Health, Boston, Massachusetts, USA.; 4 Department of Molecular Biology, University of Texas Southwestern Medical Center, Dallas, Texas, USA.; 5 Department of Urology, Institute of Urologic Science and Technology, The First Affiliated Hospital, School of Medicine, Zhejiang University, Hangzhou, Zhejiang, China.; 6 Cambridge Institute of Therapeutic Immunology and Infectious Disease (CITIID), Jeffrey Cheah Biomedical Centre, Department of Medicine, University of Cambridge, Cambridge, United Kingdom.; 7 Kidney Cancer Program, Simmons Comprehensive Cancer Center,; 8 Department of Internal Medicine, and; 9 Simmons Comprehensive Cancer Center, University of Texas Southwestern Medical Center, Dallas, Texas, USA.

**Keywords:** Genetics, Oncology, Cancer, Epigenetics, Hypoxia

## Abstract

Resistance to HIF-2α inhibitors such as belzutifan underscores the need to better understand how HIF-2α is transcriptionally regulated in clear cell renal cell carcinoma (ccRCC). Here, we uncover a cytokine-driven enhancer mechanism that sustains HIF-2α expression through the JAK1/STAT3 signaling pathway. Using a genome-wide CRISPR screen in von Hippel–Lindau–deficient (VHL-deficient) ccRCC cells, we identified SOCS3 as a key negative regulator of HIF-2α. Mechanistically, loss of SOCS3 activates JAK1/STAT3 signaling, leading to the recruitment of STAT3 to distal enhancers upstream of endothelial PAS domain-containing protein (*EPAS1*) that physically loop to its promoter to drive HIF-2α transcription. This cytokine-enhancer circuit was recapitulated in samples from patients with ccRCC and functionally validated using CRISPR interference (CRISPRi), which disrupted enhancer-promoter looping and reduced tumor growth in HIF-2α–dependent models. SOCS3 overexpression or pharmacologic inhibition of JAK1/STAT3 markedly suppressed HIF-2α expression and tumor progression both in vitro and in vivo. Unlike prior studies focusing on VHL/HIF occupancy–driven enhancer activation, this work defines a *trans*-acting cytokine–JAK1/STAT3 pathway that transcriptionally controls *EPAS1*. Together, these findings reveal a targetable enhancer mechanism that sustains HIF-2α expression and suggest that combined inhibition of JAK1/STAT3 and HIF-2α may overcome therapeutic resistance in kidney cancer.

## Abstract 2

We discovered that cytokine signaling drives kidney cancer by activating a genetic circuit that boosts HIF-2α expression, offering new targets to overcome treatment resistance.

## Introduction

In the United States, an estimated 80,980 new cases and 14,510 deaths from renal cancer (including renal cell carcinoma and renal pelvis cancer) were projected to occur in 2025 (1). The incidence of kidney cancer has been steadily rising over the past several decades, although the underlying causes remain unclear (2). Clear cell renal cell carcinoma (ccRCC) accounts for approximately 85% of all kidney cancers and is strongly associated with mutations in the von Hippel–Lindau (*VHL*) gene, with *VHL* inactivation observed in approximately 90% of patients with ccRCC (3). In conjunction with other E3 ligase components, VHL recognizes HIF-2α under normoxic conditions and facilitates its ubiquitination and subsequent proteasomal degradation. Loss of VHL leads to HIF-2α accumulation and ccRCC tumorigenesis (4–7). HIF-2α stabilization is both sufficient and necessary to promote kidney tumor growth. Furthermore, the critical role of HIF-2α in ccRCC is illustrated by studies showing that VHL-resistant HIF-2α variants can override the tumor-suppressive effects of VHL in vitro and in vivo (3, 8, 9). HIF-2α heterodimerizes with a constitutively expressed HIFβ subunit and transactivates genes with hypoxia response elements (HREs; consensus sequence NCGTG, where N represents any nucleotide)in their promoters or enhancer regions, such as VEGF and GLUT1. This results in angiogenesis, epithelial-to-mesenchymal transition, invasion and metastatic spread (6).

Given the central role of HIF-2α in ccRCC tumorigenesis, belzutifan (also known as MK-6482 or PT2977), an HIF-2α antagonist, was developed and is now approved by the FDA for the treatment of advanced RCC (10–13). Although belzutifan showed greater activity and was better tolerated than everolimus, 34% of the patients with ccRCC experienced progression as a best response. Additionally, prolonged treatment induces resistance mutations (14, 15). Mutations occurring during treatment prevent HIF-2α dimer dissociation by HIF-2α antagonists, thereby prolonging the transcription of downstream target genes. Additionally, some ccRCC models are sensitive to genetic depletion of HIF-2α but show a limited response to pharmacologic HIF-2α inhibitors. For instance, RCC10 cells grow normally in soft agar in the presence of PT2399, a first-generation HIF-2α antagonist that is structurally and mechanistically related to the clinically advanced HIF-2α inhibitor PT2977 (belzutifan), but are markedly suppressed by CRISPR/Cas9-mediated loss of HIF-2α. These observations underscore potential differences between genetic ablation and small-molecule inhibition of HIF-2α and motivate further investigation into upstream regulatory mechanisms controlling HIF-2α expression (16).

Through an unbiased, FACS-based, genome-wide CRISPR screen, we systematically investigated key regulators of HIF-2α activity. In addition to well-established regulators such as mTORC1 and ROS (17–19), we identified a number of what we believe to be novel positive and negative HIF-2α regulators. Among the top hits, the transcription/export (TREX) RNA export complex emerged as the most significant positive regulator. Conversely, SOCS3 was identified as the most significant negative regulator. SOCS3 is a well-characterized negative regulator of the JAK/STAT signaling pathway, targeting Janus kinases for proteasomal degradation (20, 21). Our findings demonstrate that SOCS3 suppressed HIF-2α expression, leading to ccRCC tumor growth inhibition. Mechanistically, SOCS3 regulates HIF-2α expression via the JAK1/STAT3 axis, with STAT3 promoting HIF-2α transcription by binding to an enhancer. Although the role of STAT3 in regulating HIF activity has been previously reported (19, 22, 23), the specific mechanism involving enhancer-mediated transcriptional activation of HIF-2α has not been previously described.

## Results

### FACS-based whole-genome CRISPR screen identifies HIF-2α positive regulators.

**Figure 1:**
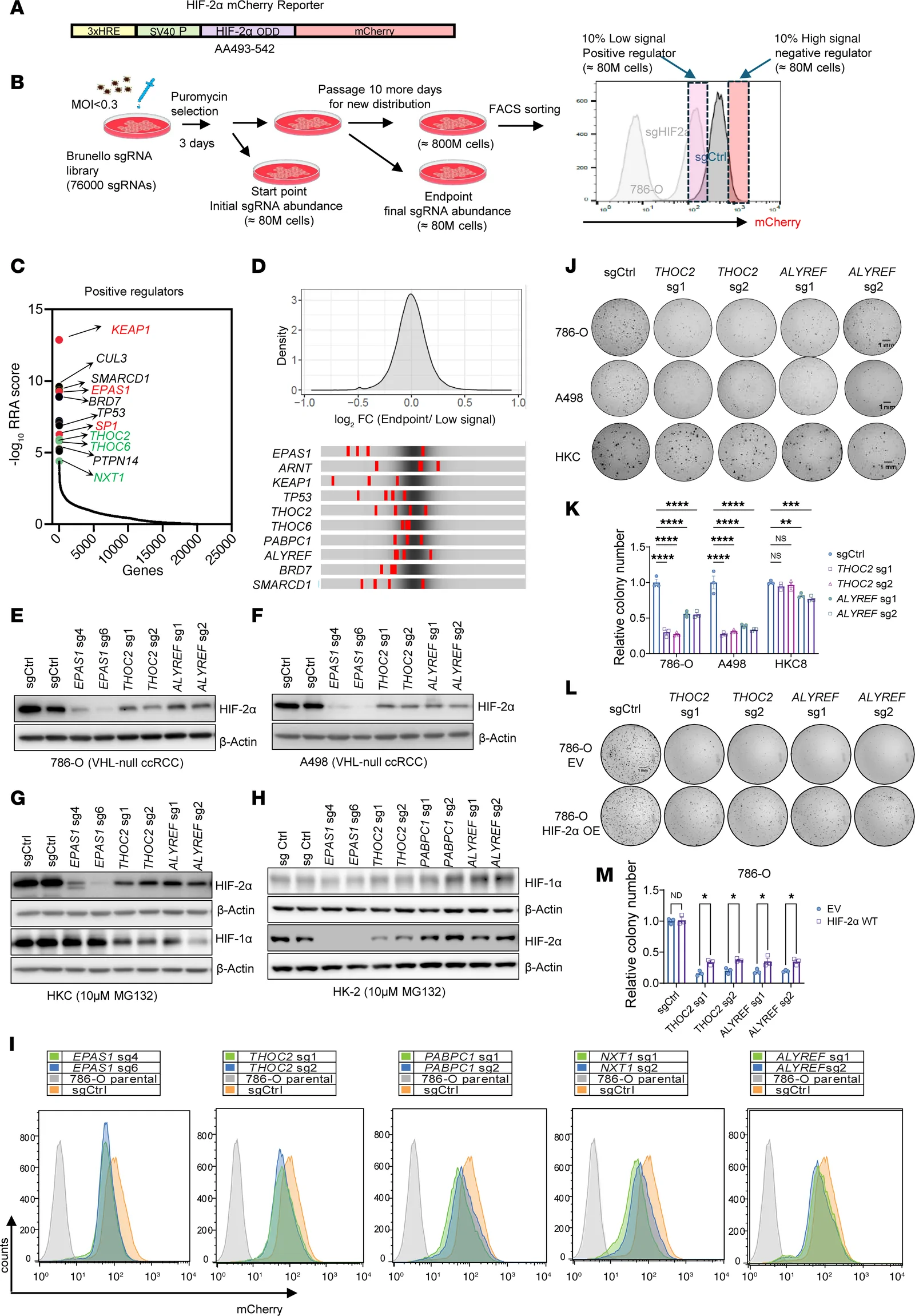
FACS-based whole-genome CRISPR screen identifies HIF-2α positive regulators. (**A**) Schematic of the HRE- HIF-2α ODD mCherry reporter construct. (**B**) Diagram of the loss-of-function, genome-wide screen using the human lentiviral sgRNA library (Brunello) in the HIF-2α reporter 786-O cell line. Ten percent of cells with either low or high signal intensity were sorted for NGS to identify factors that regulate HIF-2α activity. (**C**) Top genes identified for positive regulation of HIF-2α activity versus their log_10_ robust rank aggregation (RRA) scores in the genome-wide loss-of-function screen. Red circles are regulators reported before and green circles are genes related to the mRNA export pathway. (**D**) Distribution of sgRNA log fold change (LFC) comparing the mCherry-low group with the presorted group (positive regulators). Red bars represent 4 individual sgRNAs for the indicated genes. (**E**–**H**) Immunoblot analysis of cells with TREX component KOs. (**E** and **F**) 786-O and A498 ccRCC cell lines. (**G** and **H**) HKC and HK2 epithelial cell lines were treated with 10 μM MG132 for 6 hours prior to harvesting. (**I**)FACS-based analysis of HIF-2α activity in 786-O HIF-2α reporter cells following KO of RNA export pathway components. Each gene was targeted with 2 independent sgRNAs, and HIF-2α sgRNAs were used as positive controls. (**J** and **K**) Representative soft agar growth (**J**) and corresponding quantification (*n* = 3) (**K**) of 786-O, A498, and HKC cells expressing control (sgCtrl) or sgRNAs targeting the TREX components THOC2 and ALYREF. Scale bars: 1 mm. (**L** and **M**) Representative soft agar growth (**L**) and corresponding quantification (*n* = 3) (**M**) of 786-O cells with EV or HIF-2α overexpression that were then transduced with sgRNAs targeting TREX components or control. *P < 0.05, **P < 0.01, ***P < 0.001, and ****P < 0.0001, by 2-way ANOVA followed by Tukey’s multiple-comparison test (**J**) or multiple, unpaired *t* test (**L**). Data show the mean ± SEM.

To identify genes involved in the regulation of HIF-2α, we adapted a dynamic HIF-mCherry reporter (24) to generate a HIF-2α-specific reporter (Figure 1A). This system provides a dual-function readout for (a) genes that affect HIF function (as determined by reduced expression of target sequences) and (b) genes that affect oxygen-dependent degradation (through a chimeric protein incorporating the HIF-2α oxygen degradation domain). This dual-function reporter utilizes a triplicate consensus hypoxia response element (HRE) to drive mCherry expression upon endogenous HIF binding, but protein accumulation is also contingent on eluding VHL-mediated proteasomal degradation. The reporter was stably introduced into a VHL-null ccRCC cell line, 786-O (which expresses HIF-2α but no HIF-1α (25)) that constitutively expresses Cas9 (Supplemental Figure 1A; supplemental material available online with this article; https://doi.org/10.1172/JCI201639DS1). A single-cell clone was selected to establish a stable HIF-2α reporter cell line. This reporter system enables detection of both increased and decreased HIF-2α activity through mCherry fluorescence intensity following gene perturbation (Supplemental Figure 1, B and C).

We conducted a genome-wide mutagenesis screen by transducing Cas9-expressing 786-O HRE-HIF-2α(ODD)-mCh reporter cells with the Brunello sgRNA library (26) (Figure 1B). Following transduction, the top 10% of cells exhibiting either increased or decreased mCherry fluorescence were isolated using FACS. To identify candidate genes regulating HIF-2α activity, enriched sgRNAs from the sorted cell populations were analyzed by high-throughput deep sequencing and compared with the sgRNA distribution in the presorted cell population. HIF-2α (*EPAS1*) sgRNAs were highly enriched in the positive regulator pool (mCherry-low population), validating the robustness of our screening strategy (Figure 1, C and D). Several other top-ranked genes identified in the screen, including *KEAP1*, *CUL3*, and *SP1*, have previously been implicated in the regulation of HIF signaling, further serving as internal positive controls. *KEAP1* and *CUL3* function together as components of a CUL3-based E3 ubiquitin ligase complex that regulates NRF2, a central transcription factor in the cellular oxidative stress response (27). Elevated intracellular ROS can inhibit PHD2 activity, leading to stabilization of HIF-α proteins (28–31). In addition, SP1 has been reported to directly bind the *EPAS1* promoter and act as a transcriptional activator of HIF-2α expression (32). Together, the recovery of these established HIF regulators highlights the screen’s ability to capture both transcriptional and posttranslational mechanisms governing HIF-2α activity.

Notably, beyond these known regulators, the screen also identified multiple components of the TREX complex — including *THOC2,* *THOC6*, and *ALYREF* — as well as additional genes involved in mRNA export, such as *PABPC1* and *NXT1* (Figure 1, C and D, and Supplemental Table 1). Components of the RNA export pathway involved in HIF-2α regulation were validated across multiple cell lines, including ccRCC cell lines (786-O, A498, and UMRC2) and kidney epithelial cell lines (HKC and HK2). Notably, the effects of THOC2 and ALYREF inactivation were more pronounced in ccRCC cell lines 786-O and A498, whereas UMRC2 cells showed a comparatively modest response. This reduced sensitivity was consistent with the lower basal HIF-2α expression in UMRC2 cells relative to the other ccRCC lines examined (Figure 1, E–I, and Supplemental Figure 1, D–F). KO of TREX complex components resulted in reduced colony formation in HIF-2α–dependent ccRCC cell lines 786-O and A498 (16), while having a minimal effect on colony formation in HKC cells (Figure 1, J and K). Reexpression of HIF-2α partially rescued the growth defects observed in THOC2- or ALYREF-deficient 786-O cells (Figure 1, L and M, and Supplemental Figure 1G), indicating that impaired HIF-2α activity contributed to, but did not fully account for, the cytotoxic effects associated with TREX complex inactivation. Although the majority of mRNAs are exported via bulk export pathways, growing evidence suggests that mRNA export in mammals can be highly selective. The TREX complex has been shown to mediate the selective export of specific subsets of mRNAs, influencing a range of biological processes including DNA repair, pluripotency, stress responses, cell proliferation, survival, and hematopoiesis (33–37). Our data suggest that HIF-2α may be similarly regulated by the TREX/mRNA export pathway (Supplemental Figure 1H).

In addition, we validated 2 other top candidates, *BRD7* and *SMARCD1*, in 786-O HIF-2α reporter cells, however, their effects on HIF-2α regulation appeared to be less consistent across cell lines compared with TREX complex components, suggesting a more cell-type–specific regulation on HIF-2α (Supplemental Figure 1, I and J).

### SOCS3 is identified as the most significantly negative regulator of HIF-2α.

**Figure 2:**
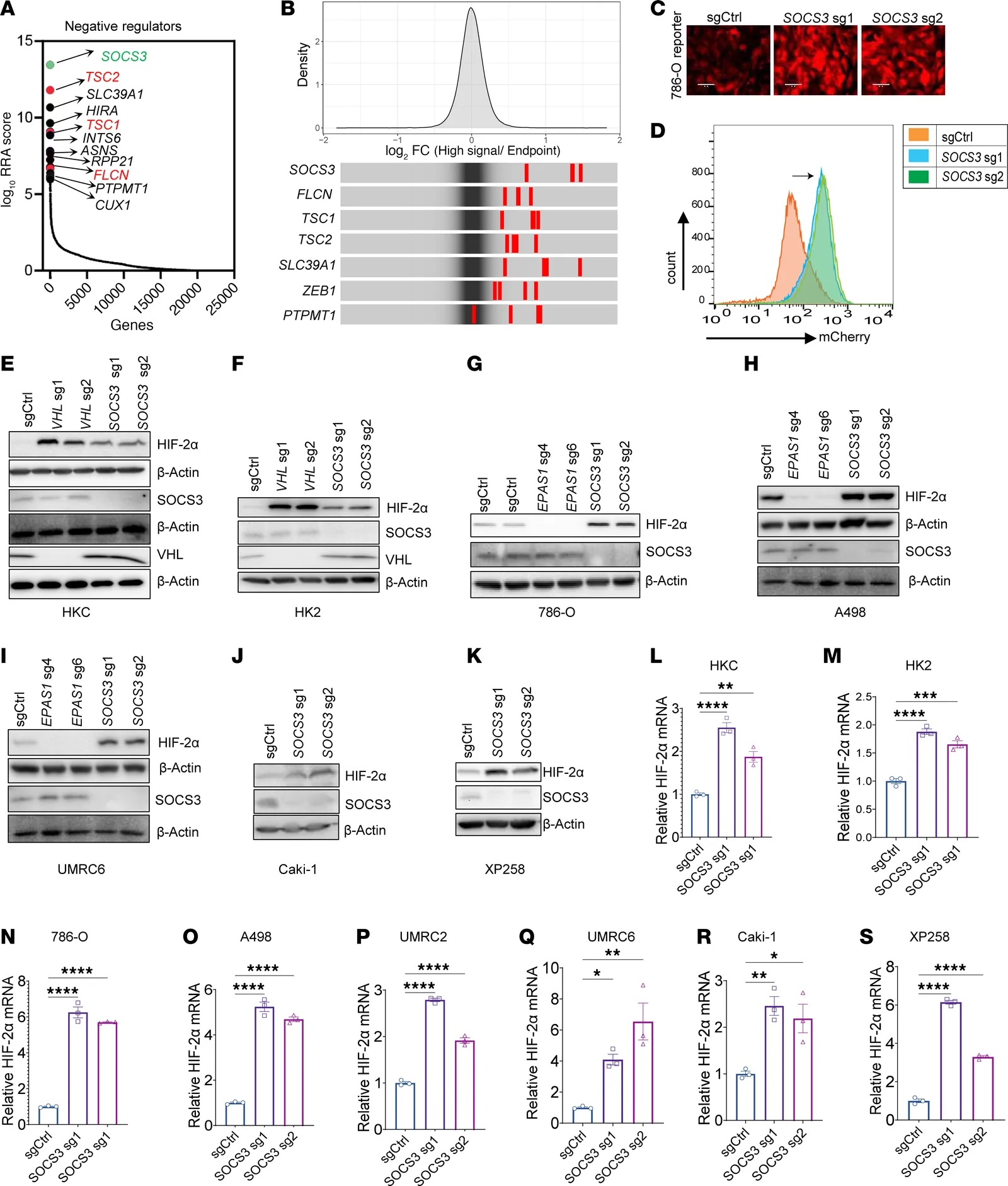
SOCS3 is identified as the most significantly negative regulator of HIF-2α. (**A**) Top genes identified for negative regulation of HIF-2α activity versus their log_10_ RRA scores in the genome-wide loss-of-function screen. Red circles are regulators reported previously. (**B**) Distribution of sgRNA LFC comparing the mCherry-enriched group with the presorted group (negative regulators). Red bars represent sgRNAs for those genes. (**C**) Representative images of mCherry fluorescence in 786-O HIF-2α reporter cells transduced with 2 individual sgRNAs targeting *SOCS3*. Scale bars: 9μM. (**D**) FACS-based analysis of HIF-2α activity in 786-O HIF-2α reporter cells following *SOCS3* KO using 2 independent sgRNAs. (**E** and **F**) Immunoblot analysis of the kidney epithelial cell lines HKC (**E**) and HK-2 (**F**) transduced with sgRNAs targeting *SOCS3* or *VHL* using the indicated antibodies. (**G**–**K**) Immunoblot analysis of the ccRCC cell lines 786-O (**G**), A498 (**H**), UMRC6 (**I**), Caki-1 (**J**), and PDX XP258 cells (**K**) transduced with sgRNAs targeting *SOCS3* or *EPAS1* using the indicated antibodies. (**L**–**S**) RT-qPCR quantification of *HIF2A* mRNA levels of the indicated cell lines transduced with sgCtrl or sgRNAs targeting *SOCS3*. *P < 0.05, **P < 0.01 ***P < 0.001, and ****P < 0.0001, by 1-way ANOVA followed by Tukey’s multiple-comparison test. Data show the mean ± SEM.

In our investigation of HIF-2α negative regulators, VHL was not recovered in the CRISPR screen, which was expected, given that 786-O cells are VHL null. While HIF stability and transcriptional activity are primarily controlled by oxygen availability and VHL-mediated degradation, the mTORC1 pathway also plays a key role in regulating HIF expression, particularly by modulating HIF mRNA translation (17, 38–40). We identified several canonical mTORC1 inhibitors including *TSC1* and *TSC2* as the top-enriched candidates (Figure 2, A and B, and Supplemental Table 2), further validating the robustness of our screening approach. Among all the HIF-2α negative regulators, SOCS3 emerged as the most significantly enriched candidate. Targeting SOCS3 with 2 independent sgRNAs produced a robust increase in mCherry fluorescence intensity in 786-O HIF-2α reporter cells, as assessed by both fluorescence imaging and FACS analysis (Figure 2, C and D). This finding was further validated in an independent cell model, UMRC2 HIF-2α reporter cells, in which SOCS3 KO similarly resulted in a substantial elevation of mCherry signal (Supplemental Figure 2, A and B). We further confirmed the suppressive role of SOCS3 in regulating HIF-2α by examining HIF-2α protein levels via Western blotting across multiple cell lines, including kidney epithelial cell lines (HK-2 and HKC), ccRCC cell lines (786-O, A498, UMRC6, and Caki-1), as well as a patient-derived xenograft (PDX) cell line, XP258 (Figure 2, E–K). In addition to protein analysis, we evaluated the effect of SOCS3 depletion on HIF-2α transcript levels. Reverse transcription quantitative PCR (RT-qPCR) analysis revealed a significant upregulation of HIF-2α mRNA in SOCS3-KO cells across multiple cell lines (i.e., HKC, HK-2, 786-O, A498, UMRC6, Caki-1, and XP258) (Figure 2, L–S). To determine whether this increase reflected altered mRNA stability, we treated cells with the transcriptional inhibitor actinomycin D and performed pulse-chase analyses to assess *HIF2A* mRNA decay. We show that, in 786-O, *EPAS1* mRNA in SOCS3-KO cells had faster turnover than did control cells, while in A498, SOCS3-KO cells showed comparable degradation rates. Even though SOCS3-KO cells had a faster degradation rate in some contexts, *EPAS1* mRNA levels remained consistently higher in SOCS3-deficient cells compared with sgControl (sgCtrl) cells throughout the chase period. From these observations, we conclude that SOCS3 regulated HIF-2α expression through mRNA transcription rather than by modulating mRNA stability (Supplemental Figure 2, C and D). While SOCS3 depletion robustly increased HIF-2α expression, we also observed a modest but reproducible induction of HIF-1α following SOCS3 inactivation. Across all cell lines examined, *HIF2A* mRNA levels typically increased by approximately 1.5-fold, whereas *HIF2A* mRNA levels increased by 2- to 8-fold under the same conditions (Supplemental Figure 2, E–K). These findings indicate that, although SOCS3 could regulate both HIF-α paralogs, HIF-2α was substantially more sensitive to SOCS3-mediated suppression.

Importantly, the comparatively limited induction of HIF-1α should be interpreted in the context of its distinct and context-dependent role in ccRCC. Genetic and functional studies from the Kaelin group and others have demonstrated that HIF-1α can act as a tumor suppressor in ccRCC, with frequent loss of the *HIF1A* locus on chromosome 14q and tumor-inhibitory effects of HIF-1α reexpression in renal cancer models (41). In contrast, HIF-2α functions as a dominant oncogenic driver in ccRCC. Thus, the preferential effect of SOCS3 loss on HIF-2α expression, coupled with only modest effects on HIF-1α, further supports a selective role for SOCS3 in restraining oncogenic HIF-2α signaling rather than broadly activating hypoxia responses.

Consistent with increased HIF-2α abundance, the expression of several canonical HIF-2α target genes — including *VEGFA*, *CCND1*, and *NDRG1* — was often elevated following SOCS3 depletion (Supplemental Figure 2L), indicating that SOCS3 loss can enhance the transcriptional output of HIF-2α signaling, although the response may vary depending on the cellular context. Similar effects were observed in U2OS cells, a non-ccRCC osteosarcoma line, where SOCS3 KO led to increased HIF-2α mRNA and protein levels, as well as upregulation of its downstream targets (Supplemental Figure 2, M–P), suggesting that this regulatory mechanism is conserved across different cell types.

### SOCS3 overexpression suppresses HIF-2α levels and inhibits ccRCC tumorigenesis.

**Figure 3:**
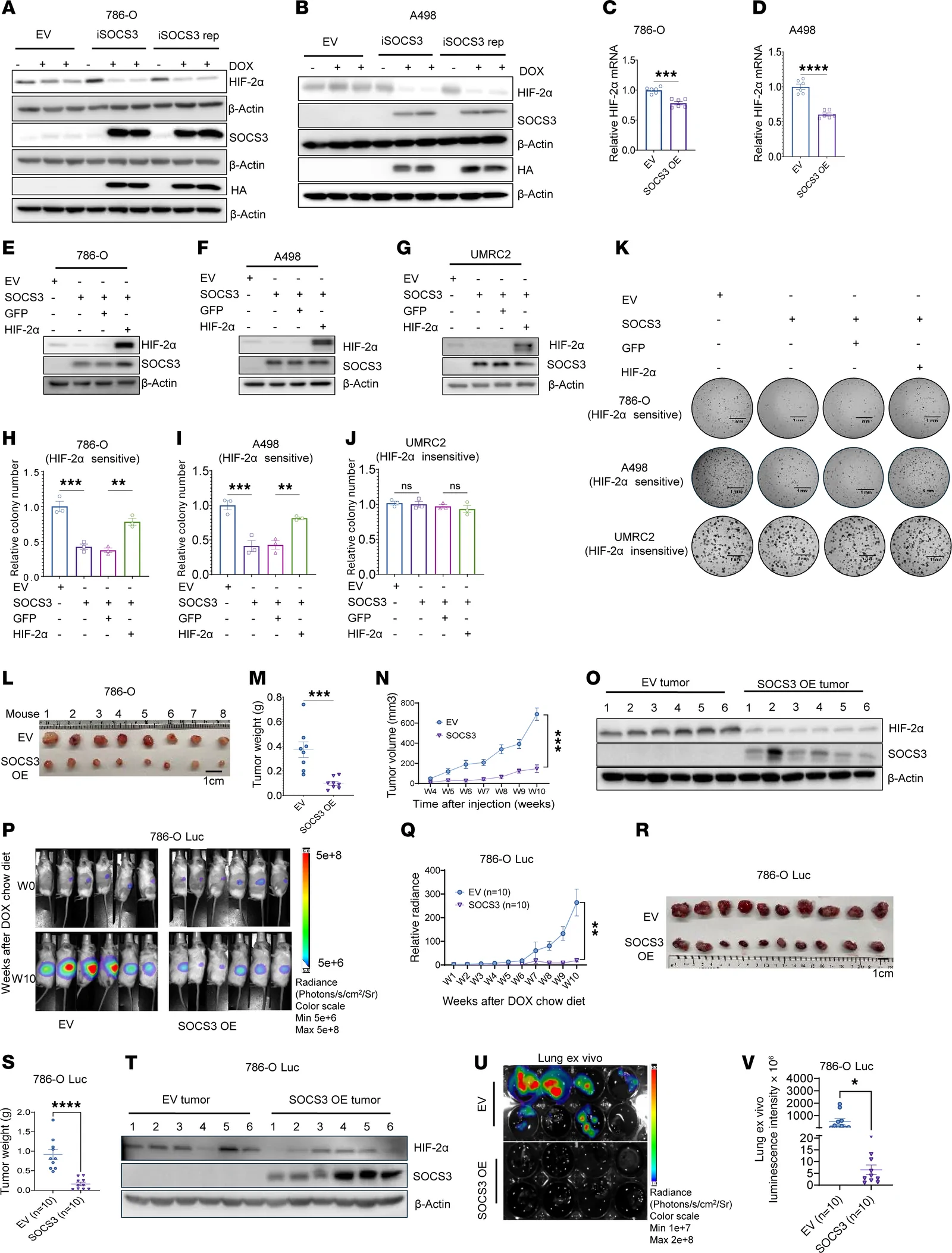
SOCS3 overexpression suppresses HIF-2α levels and inhibits ccRCC tumorigenesis. (**A** and **B**) Immunoblots of ccRCC cell lines 786-O (**A**) and A498 (**B**) expressing DOX-inducible EV or SOCS3, treated with 200 ng/mL DOX. iSOCS3-rep, independent biological replicates of DOX-induced SOCS3 expression. (**C** and **D**) RT-qPCR quantification of HIF-2α mRNA levels in 786-O (**C**) and A498 (**D**) cells with EV or SOCS3 expression induced by 200 ng/mL DOX. (**E**–**G**) Immunoblots of 786-O (**E**), A498 (**F**), and UMRC2 (**G**) cells expressing EV or DOX-induced SOCS3 and coexpressing SOCS3 and GFP or HIF-2α. (**H**–**J**) Soft agar growth quantification of 786-O (**H**), A498 (**I**), and UMRC2 (**J**) cells expressing EV or DOX-induced SOCS3, and coexpressing SOCS3 and GFP or HIF-2α. (**K**) Representative soft agar growth of 786-O, A498, and UMRC2 cells expressing EV or DOX-induced SOCS3 and coexpressing SOCS3 and GFP or HIF-2α (*n* = 3). Scale bars: 1 mm. (**L**–**O**) 786-O cells transduced with DOX-inducible SOCS3 or EV subcutaneous injection. Image of tumors after dissection (scale bar: 1 cm) (**L**), tumor weights (**M**), subcutaneous tumor growth (**N**), and immunoblots of tumor lysates (**O**). (**P**–**V**) 786-O cell lines with SOCS3-inducible expression were injected orthotopically into the renal capsule of NSG mice. Representative bioluminescence images at 0 weeks and 10 weeks after DOX treatment (**P**), corresponding quantification data (**Q**), Image of kidney orthotopic tumors (scale bar: 1 cm) (**R**), tumor weights (**S**), immunoblots of representative tumor samples (**T**), representative lung ex vivo bioluminescence images (**U**), and quantification of ex vivo imaging (**V**). Data show the mean ± SEM. *P < 0.05, **P < 0.01, and ***P < 0.001, by 1-way ANOVA followed by Tukey’s multiple-comparison test (**H**–**J**) or unpaired Student’s *t* test (**C**, **D**, **M**, **N**, **Q**, **S**, and **V**).

To further investigate the regulatory function of SOCS3 in ccRCC, we established a doxycycline-inducible (DOX-inducible) SOCS3 overexpression system in 3 independent ccRCC cell lines: 786-O, A498, and UMRC2. In 786-O cells, SOCS3 expression increased in a DOX dose–dependent manner, which was accompanied by a progressive reduction in HIF-2α protein levels; a concentration of 200 ng/mL DOX was sufficient to induce robust SOCS3 expression and a significant decrease in HIF-2α abundance (Supplemental Figure 3, A and B). On the basis of these dose-response experiments, we selected 200 ng/mL DOX for subsequent analyses. Treatment of 786-O and A498 inducible cell lines with 200 ng/mL DOX resulted in a consistent reduction of HIF-2α protein levels (Figure 3, A and B). This suppression was also reflected at the transcriptional level, as *HIF2A* mRNA expression was significantly downregulated upon SOCS3 induction, as measured by RT-qPCR (Figure 3, C and D). Given the inhibitory effect of SOCS3 on HIF-2α expression, we next evaluated its functional consequences on cell growth using 3D soft agar colony formation assays. In both 786-O and A498 cells, SOCS3 overexpression impaired cell growth (Figure 3, E, F, H, I, and K). In contrast, UMRC2 cells, which are less reliant on HIF-2α signaling (16), were largely unaffected. This is consistent with the known dependency of 786-O and A498 cells on HIF-2α for proliferation. To confirm that the observed growth defect was mediated through HIF-2α suppression, we induced ectopic reexpression of HIF-2α in SOCS3-overexpressing cells. Restoration of HIF-2α rescued colony formation in 786-O and A498 cells, indicating that the growth suppression resulting from SOCS3 overexpression was HIF-2α dependent (Figure 3, G, J, and K).

To assess the effect of SOCS3 overexpression on tumor growth in vivo, we performed subcutaneous xenograft experiments using DOX-inducible 786-O and A498 cells. Mice treated with DOX to induce SOCS3 expression developed significantly smaller tumors than did control mice, as evidenced by reduced tumor volumes and weights (Figure 3, L–N, and Supplemental Figure 3, C–E). Immunoblot analysis of tumor lysates confirmed sustained SOCS3 induction and a corresponding downregulation of HIF-2α in these tumors (Figure 3O and Supplemental Figure 3F).

To further investigate the effects of SOCS3 overexpression in a more physiologically relevant setting, we established an orthotopic model by injecting luciferase-expressing 786-O cells into the renal capsule of NSG mice. SOCS3 induction by DOX significantly suppressed tumor growth, as revealed by longitudinal bioluminescence imaging and endpoint tumor weight measurements (Figure 3, P–S). Immunoblot analysis of orthotopic tumor lysates demonstrated reduced HIF-2α protein levels in response to SOCS3 expression (Figure 3T). SOCS3 overexpression also markedly suppressed lung metastasis, as shown by ex vivo bioluminescence imaging and quantification (Figure 3, U and V). Together, these results demonstrate that SOCS3 acted as a potent suppressor of HIF-2α expression and ccRCC tumor progression. Its overexpression not only reduced HIF-2α transcription and protein stability but also impaired tumor growth and metastatic dissemination in vivo.

### Loss of SOCS3 leads to JAK1 activation and promotes HIF-2α transcription.

**Figure 4:**
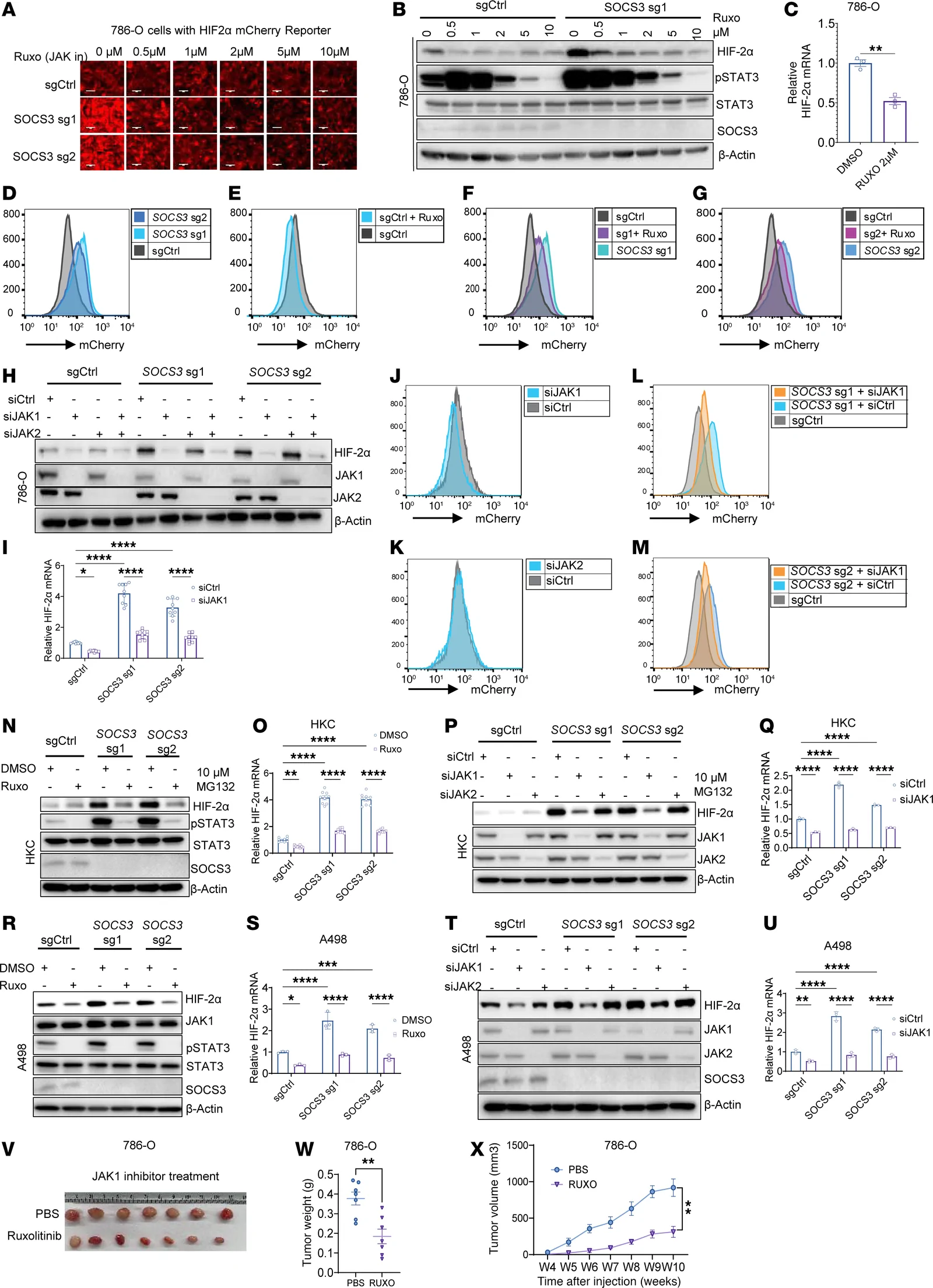
Loss of SOCS3 leads to JAK1 activation and promotes HIF-2α transcription. (**A**) Representative fluorescence images of 786-O reporter cells transduced with the indicated sgRNA and ruxolitinib (Ruxo) for the indicated concentration for 48 hours. Scale bars: 90µm. (**B**) Corresponding immunoblots analysis for **A**. (**C**) RT-qPCR quantification of *HIF2A* mRNA for 786-O cells treated with the indicated inhibitor for 48 hours. (**D**–**G**) FACS-based analysis of HIF-2α activity in 786-O reporter cells. Cells were transduced with the indicated sgRNA and treated with 2 μM ruxolitinib for 48 hours. (**H**) Immunoblots of 786-O cells transduced with the indicated sgRNA and siRNA. (**I**) RT-qPCR quantification of *HIF2A* mRNA for 786-O cells transduced with the indicated sgRNA and siRNA for 48 hours. (**J**–**M**) FACS-based analysis of HIF-2α activity in 786-O reporter cells upon treatment with the indicated sgRNA and siRNA. (**N** and **O**) Immunoblots (**N**) and RT-qPCR quantification of *HIF2A* mRNA (**O**) for HKC cells transduced with the indicated sgRNA and ruxolitinib for 48 hours. Cells for immunoblots were treated with 10 μM MG132 for 6 hours. (**P** and **Q**) Immunoblots (**P**) and RT-qPCR quantification of *HIF2A* mRNA levels (**Q**) of HKC cells transduced with the indicated sgRNA and siRNA. Cells for immunoblots were treated with 10 μM MG132 for 6 hours. (**R** and **S**) Immunoblots (**R**) and RT-qPCR quantification of HIF-2α mRNA (**S**) for A498 cells transduced with indicated sgRNA and ruxolitinib for 48 hours. (**T** and **U**) Immunoblots (**T**) and RT-qPCR quantification of *HIF2A* mRNA (**U**) for A498 cells transduced with the indicated sgRNA and siRNA. (**V**–**X**) Image of tumors (**V**), tumor weights after dissection (**W**), and tumor growth (**X**) after treatment with the JAK1 inhibitor ruxolitinib (120 mg/kg/d, oral gavage) in the 786-O–derived xenograft model (NSG, *n* = 7). *P < 0.05, **P < 0.01, ***P < 0.001, and ****P < 0.0001, by 2-way ANOVA followed by Tukey’s multiple-comparison test (**I**, **O**, **Q**, **S**, and **U**) or 2-tailed Student’s *t* test (**C**, **W**, and **X**). Data show the mean ± SEM.

SOCS3 is a known inhibitor of Janus kinases, including JAK1 and JAK2, acting through its SH2 domain and kinase-inhibitory region to block kinase activity and promote proteasomal degradation (20, 21). To determine whether the elevated HIF-2α expression observed upon SOCS3 loss is mediated through JAK signaling, we treated SOCS3-deficient 786-O HIF-2α reporter cells with ruxolitinib, a clinically approved dual JAK1/2 inhibitor. Ruxolitinib treatment significantly reduced HIF-2α level induced by SOCS3 KO, as shown by fluorescence microscopy, FACS analysis, Western blotting, and RT-qPCR (Figure 4, A–G, and Supplemental Figure 4, A–D), suggesting that JAK signaling is required for HIF-2α transcription in the absence of SOCS3. In contrast, other JAK/STAT pathway inhibitors tested at the indicated concentrations were notably less effective in suppressing HIF-2α expression (Supplemental Figure 4, A–C). To distinguish between JAK1 and JAK2, we performed siRNA-mediated knockdown of each kinase in SOCS3-deficient 786-O reporter cells. Knockdown of JAK1, but not JAK2, suppressed both HIF-2α protein and mRNA expression and diminished mCherry reporter activity (Figure 4, H–M). These results established JAK1 as the dominant effector responsible for HIF-2α induction in this context. To further substantiate the role of JAK1, we tested itacitinib, a structurally distinct and highly selective JAK1 inhibitor. Similar to ruxolitinib, itacitinib suppressed SOCS3 loss–induced HIF-2α expression in a dose-dependent manner (Supplemental Figure 4, E and F), providing pharmacologic confirmation of JAK1 dependence. We next assessed whether this mechanism was conserved across additional models. In HKC cells, SOCS3 KO elevated HIF-2α expression, which was reversed by either ruxolitinib treatment or JAK1 knockdown, as shown by immunoblot and RT-qPCR analyses (Figure 4, N–Q). Similarly, in A498 ccRCC cells, JAK1 inhibition or knockdown reduced HIF-2α levels induced by SOCS3 loss, whereas JAK2 silencing had no significant effect (Figure 4, R–U). To confirm the clinical relevance of this axis, we examined XP258, a patient-derived ccRCC model (42). SOCS3 KO in XP258 cells also increased HIF-2α, which was suppressed by ruxolitinib treatment (Supplemental Figure 4, G and H), further supporting a conserved SOCS3/JAK1/HIF-2α–regulatory mechanism.

We then assessed whether JAK inhibition could suppress ccRCC cell growth. In 3D soft agar assays, ruxolitinib treatment significantly reduced colony formation in 786-O and A498 cells, but had a minimal effect on UMRC2 cells (Supplemental Figure 4, I and J). This pattern aligns with our SOCS3 overexpression experiments in which 786-O and A498 — but not UMRC2 — showed growth suppression upon HIF-2α inhibition, reflecting their differential dependence on HIF-2α signaling (16). Overexpression of HIF-2α in 786-O cells conferred increased resistance to ruxolitinib treatment, indicating that the cytotoxic effects of ruxolitinib were mediated, at least in part, through suppression of HIF-2α activity (Supplemental Figure 4, K and L). Importantly, RCC10 cells — which remain dependent on HIF-2α signaling yet are resistant to pharmacologic HIF-2α antagonists — were nevertheless sensitive to ruxolitinib treatment (Supplemental Figure 4, M and N). These findings suggest that targeting the SOCS3/JAK1 axis can suppress HIF-2α–dependent tumor growth through mechanisms distinct from direct HIF-2α binding and may therefore represent a therapeutic strategy for treating ccRCC tumors that have acquired resistance to HIF-2α inhibitors such as belzutifan.

To evaluate the in vivo relevance of JAK signaling, we treated NSG mice bearing 786-O xenografts with ruxolitinib. JAK1/2 inhibition significantly impaired tumor growth and reduced endpoint tumor weights compared with vehicle-treated controls (Figure 4, V–X), demonstrating that JAK activity supported in vivo tumor progression in HIF-2α–dependent ccRCC, even in the absence of SOCS3 perturbation.

Collectively, these findings support a model in which JAK1/HIF-2α signaling promotes both ccRCC cell growth and in vivo tumor development in ccRCC. These results highlight the SOCS3/JAK1/HIF-2α axis as a critical regulatory pathway and a potential therapeutic target in HIF-2α–driven kidney cancer.

### STAT3 mediates the effect of SOCS3/JAK1 on HIF-2α expression.

**Figure 5:**
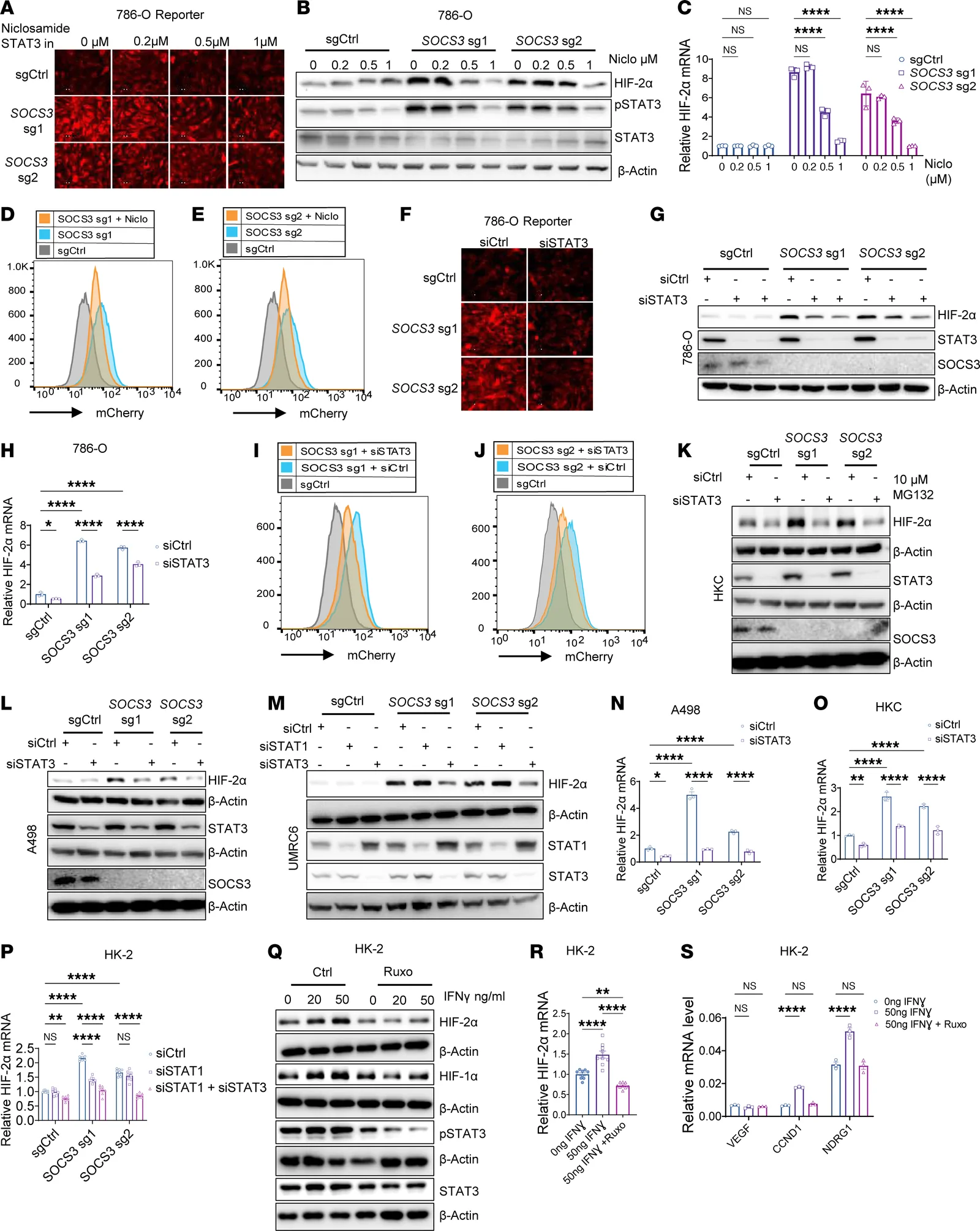
STAT3 mediates the effect of SOCS3/JAK1 on HIF-2α expression. (**A**–**C**) Representative fluorescence imaging (**A**) of 786-O HIF-2α mCherry reporter cells transduced with *SOCS3* KO and treated with increasing concentrations of niclosamide, a STAT3 inhibitor for 48 hours, corresponding immunoblot analysis of HIF-2α and STAT3 levels (**B**), and RT-qPCR quantification of *HIF2A* mRNA levels (**C**). (**D** and **E**) FACS-based analysis of HIF-2α activity in 786-O reporter cells transduced with *SOCS3* sgRNAs sg1 (**D**) and sg2 (**E**) and treated with 1 μM niclosamide. (**F**–**H**) Representative fluorescence images (**F**) of 786-O HIF-2α mCherry reporter cells transduced with *SOCS3* KO combined with STAT3 siRNA and the corresponding immunoblot analysis (**G**) and RT-qPCR quantification of *HIF2A* mRNA levels (**H**). (**I** and **J**) FACS-based analysis of HIF-2α activity in 786-O reporter cells transduced with *SOCS3* sgRNA sg1 (**I**) and sg2 (**J**) combined with STAT3 siRNA. (**K** and **L**) Immunoblot analysis of HKC cells (**K**) and A498 cells (**L**) transduced with sgCtrl or *SOCS3* sg1 and sg2 and treated with STAT3 siRNA for 48 hours. HKC cells were treated with 10 μM MG132 for 6 hours before harvesting. (**M**) Immunoblot analysis of UMRC6 cells transduced with sgCtrl or *SOCS3* sg1 and sg2 and treated with STAT1 siRNA or STAT3 siRNA for 48 hours. (**N**–**P**) RT-qPCR quantification of *HIF2A* mRNA levels of A498 cells (**N**), HKC cells (**O**), and HK-2 cells (**P**) transduced with sgCtrl or *SOCS3* sg1 and sg2 and treated with the indicated siRNAs for 48 hours. (**Q**–**S**) Immunoblot analysis (**Q**), RT-qPCR quantification of *HIF2A* mRNA levels (**R**) and RT-qPCR analysis (**S**) of HIF-2α downstream targets for HK-2 cells treated with IFN-γ at the indicated concentration and combined with ruxolitinib treatment. *P < 0.05, **P < 0.01, and ****P < 0.0001, by 2-way ANOVA followed by Tukey’s multiple-comparison test (**C**, **H**, **N**–**P**, and **S**) or 1-way ANOVA followed by Tukey’s multiple-comparison test (**Q**). Data show the mean ± SEM.

Having established JAK1 as the principal mediator of HIF-2α upregulation following SOCS3 loss, we next investigated the downstream effector responsible for this regulation. STAT3 is a well-characterized transcription factor activated by JAK1, and although it has been shown to regulate HIF-1α in other tumor contexts (19, 22, 23, 43), its role in modulating HIF-2α (EPAS1) remains largely unexplored. To determine whether STAT3 contributes to HIF-2α regulation in ccRCC, we first treated 786-O HIF-2α reporter cells with niclosamide, a small-molecule inhibitor of STAT3 (44). Niclosamide treatment led to a dose-dependent reduction in mCherry fluorescence, HIF-2α protein levels, and *EPAS1* mRNA expression following SOCS3 inactivation (Figure 5, A–E). Importantly, niclosamide also suppressed the expression of multiple HIF-2α downstream target genes, including *VEGFA*, *CCND1*, and *NDRG1*, in a dose-dependent manner upon SOCS3 inactivation (Supplemental Figure 5, A–D). However, niclosamide had a limited effect on HIF-2α expression in SOCS3-proficient cells, suggesting that its inhibitory effects were more prominent in the context of JAK/STAT pathway activation. Because small-molecule inhibitors like niclosamide can exhibit off-target effects, incomplete pathway inhibition, and variable cellular uptake, we used siRNA-mediated knockdown of STAT3 to validate specificity. STAT3 knockdown not only recapitulated the niclosamide effects in SOCS3-deficient 786-O cells, but also reduced HIF-2α levels in parental (SOCS3-proficient) cells (especially at the mRNA level), indicating that STAT3 was required for basal HIF-2α expression as well (Figure 5, F–J). This STAT3-dependent regulation was conserved across cell types; STAT3 knockdown similarly suppressed HIF-2α protein levels in A498, HKC, UMRC6, and HK-2 cells (Figure 5, K–O). In contrast, STAT1 knockdown had a minimal effect on HIF-2α expression in these cells (Figure 5, M and P, and Supplemental Figure 5, E and F), reinforcing the idea that STAT3 is the predominant transcriptional regulator of HIF-2α in this pathway.

To further validate that STAT3 activation is sufficient to induce HIF-2α expression, we treated HK-2 cells with IFN-γ, a cytokine known to activate JAK1/-2/STAT signaling (45–47). IFN-γ stimulation resulted in increased levels of phosphorylated STAT3 (p-STAT3) and elevated HIF-2α protein expression (Figure 5Q). We observed a similar result in HKC cells (Supplemental Figure 5G). Consistent with the enhanced HIF-2α activity, IFN-γ treatment also led to significant upregulation of HIF-2α downstream target genes, including *CCND1* and *NDRG1*. Importantly, cotreatment with ruxolitinib effectively inhibited IFN-γ–induced HIF-2α upregulation, demonstrating that this response was dependent on JAK activity and confirming that the JAK/STAT3 pathway mediated cytokine-driven HIF-2α induction (Figure 5, R and S).

Together, these results establish STAT3 as both necessary and sufficient for HIF-2α induction, acting downstream of JAK1 and providing a direct mechanistic link between cytokine signaling and HIF-2α activation. This identifies the SOCS3/JAK1/STAT3/HIF-2α axis as what we believe to be a novel and targetable regulatory pathway in ccRCC.

### STAT3 binds to the enhancer region upstream of the HIF-2α gene to regulate its expression.

**Figure 6:**
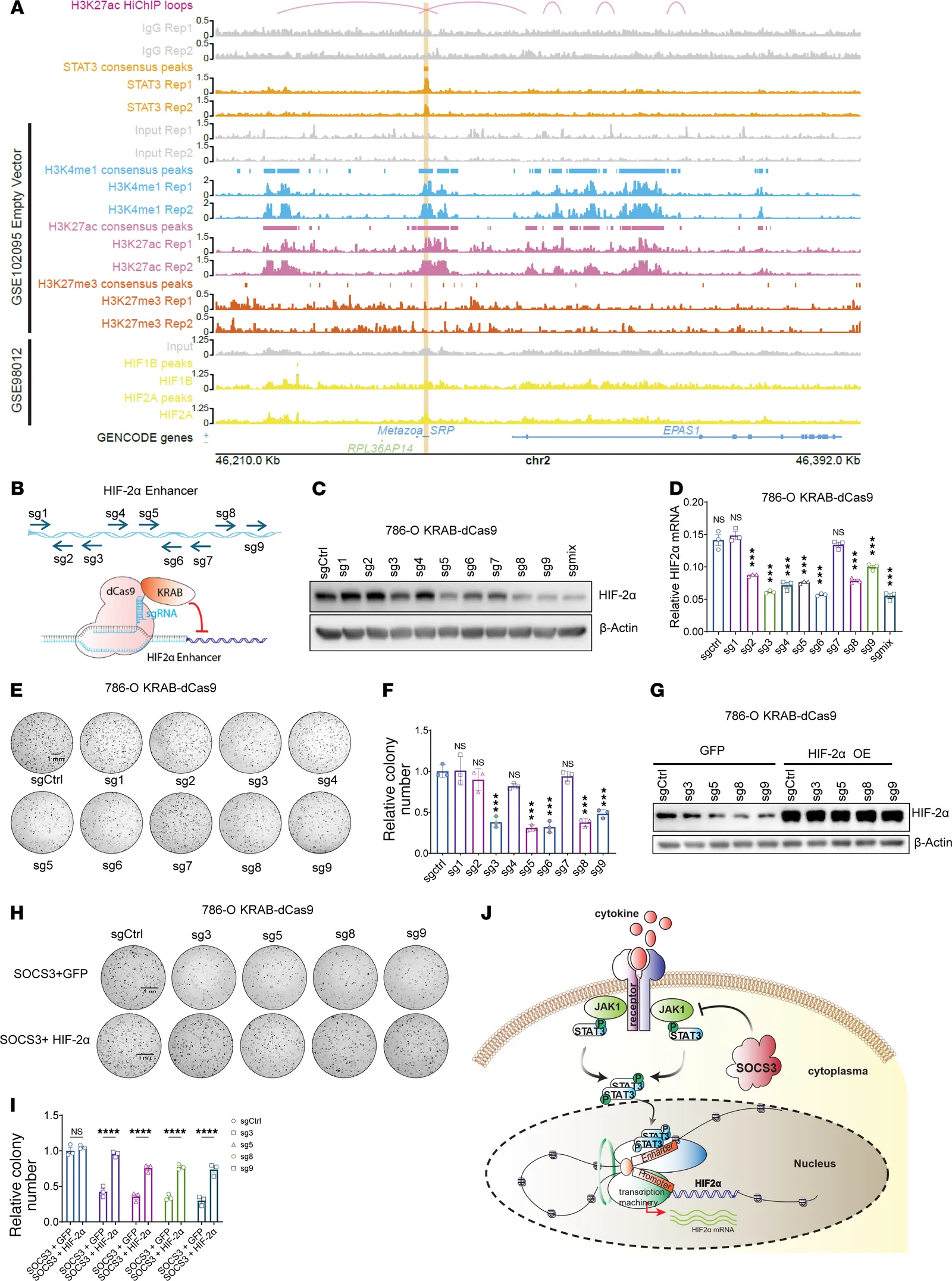
STAT3 binds to the enhancer region upstream of the HIF-2α gene to regulate its expression. (**A**) HiChIP chromatin interaction analysis and ChIP-seq binding peaks for STAT3, HIF-2α, HIF1B, H3K4me1, H3K4me3, H3K27ac, and H3K27me3 upstream of the HIF-2α gene (*EPAS1*) in 786-O cells. (**B**) Schematic representation of the CRISPRi system (dCas9-KRAB) and the targeted sites within the HIF-2α enhancer region indicated in **A**. (**C** and **D**) Immunoblot analysis (**C**) and RT-qPCR quantification of *HIF2A* mRNA levels (**D**) in cells treated with the dCas9-KRAB system targeting the HIF-2α enhancer region using 9 individual sgRNAs. (**E** and **F**) Representative soft agar growth (*n* = 3) (**E**) and corresponding quantification (**F**) of cells from **B**. (**G**–**I**) Immunoblot analysis (**G**), representative soft agar growth (**H**), and corresponding quantification (**I**) of cells reexpressing HIF-2α after applying the dCas9-KRAB system. (**J**) Schematic representation of the SOCS3-mediated regulation of HIF-2α expression through the JAK1/STAT3 signaling pathway. Cytokine binding activates JAK1, leading to STAT3 phosphorylation. In the absence of SOCS3, STAT3 is hyperactivated and binds to enhancer regions located upstream of the HIF-2α gene (*EPAS1*), facilitating the formation of a long-range chromatin loop between the enhancer and the HIF-2α promoter. This interaction promotes transcriptional activation of HIF-2α. In the presence of SOCS3, it inhibits JAK1 activity, preventing STAT3 phosphorylation and subsequent HIF-2α upregulation. CRISPRi (dCas9-KRAB) targeting of STAT3-bound enhancers leads to reduced HIF-2α expression and impaired tumorigenic potential in ccRCC cells. ***P < 0.001 and ****P < 0.0001, by 1-way ANOVA followed by Tukey’s multiple-comparison test (**D** and **F**) or 2-way ANOVA followed by Tukey’s multiple-comparison test (**I**). Data represent the mean ± SEM.

To investigate the mechanism by which STAT3 regulates *EPAS1* (HIF-2α) expression, we performed STAT3 ChIP-seq in 786-O cells, which revealed 2 prominent STAT3-binding peaks located approximately 60 kb and 20 kb upstream of the *EPAS1* transcription start site (TSS). These peaks overlap with active enhancer histone marks, including H3K27ac and H3K4me1, consistent with enhancer activity. Hi-ChIP chromatin interaction data showed a looping interaction between these STAT3-bound regions and the *EPAS1* promoter for the 20 kb enhancer (Figure 6A), supporting a model of long-range transcriptional regulation. In contrast, the –60 kb STAT3-bound region showed weaker or inconsistent promoter interactions under the conditions examined, suggesting a more context-dependent or auxiliary regulatory role. Consistent with the findings from 786-O cells, we also observed STAT3 binding at the same enhancer regions at the 20 kb upstream region in HKC cells, reinforcing the notion that this enhancer-mediated regulation is conserved across multiple cell lines (Supplemental Figure 6A). Moreover, tumor samples from 2 patients with RCC exhibited increased activity at these 2 enhancers compared with their respective normal controls, suggesting that these enhancers may serve to enhance transcription of HIF-2α in tumors (Supplemental Figure 6B) (48). On the basis of its strong promoter contact and reproducibility across datasets, we therefore focused our functional studies on the –20 kb enhancer.

To determine whether this enhancer is required for endogenous HIF-2α expression, we applied CRISPRi using dCas9-KRAB targeting the STAT3-bound sites. Repression of the enhancers led to a marked reduction in both *EPAS1* mRNA and HIF-2α protein levels (Figure 6, B–D) and significantly impaired colony formation in a 3D soft agar assay (Figure 6, E and F), confirming their functional importance. To further verify the specificity of the enhancer-mediated regulation, we performed a rescue experiment by reexpressing HIF-2α in CRISPRi-treated cells. Notably, HIF-2α reexpression successfully restored the colony formation ability, indicating that the loss of tumorigenic capacity upon enhancer repression was directly attributable to reduced HIF-2α levels (Figure 6, G–I). These findings highlight the critical role of STAT3-bound enhancers in maintaining HIF-2α expression and demonstrate that disruption of these enhancers specifically compromised HIF-2α–dependent tumor growth.

Previous studies have shown that STAT3 regulates HIF-1α, often via posttranslational mechanisms such as inhibition of phosphorylated von Hippel–Lindau–mediated (p-VHL-mediated) degradation (23). However, the regulation of HIF-2α by STAT3 has remained largely unexplored. Our findings identify what we believe to be a novel mechanism in which STAT3 binds to and activates distal enhancer elements located upstream of *EPAS1*, promoting HIF-2α transcription. This enhancer-mediated regulation is distinct from the known STAT3–HIF-1α interaction and reveals a previously unrecognized cytokine-responsive pathway controlling HIF-2α expression in ccRCC.

## Discussion

Our genome-wide CRISPR/Cas9 screen revealed a comprehensive regulatory network that governs HIF-2α activity in ccRCC. Among the top hits, we identified both known and what we believe novel modulators of HIF-2α, including components of the TREX complex as the most significantly enriched positive regulators, and SOCS3 as the most significantly enriched negative regulator. Our findings uncovered a broad spectrum of what we believe to be previously unrecognized HIF-2α regulators — including both transcriptional and posttranscriptional factors — that collectively shape the HIF-2α–regulatory landscape in ccRCC (Supplemental Tables 1 and 2). These candidates not only deepen our understanding of the upstream control of *EPAS1* but also represent a rich source of potential therapeutic targets. Given the clinical success, yet limitations, of direct HIF-2α inhibitors such as belzutifan (14, 16) — including the emergence of resistance and incomplete responses — targeting these previously unrecognized regulators may offer alternative or complementary strategies to more effectively suppress HIF-2α signaling and improve patient outcomes.

Although the regulation of HIF-2α protein stability via oxygen-dependent hydroxylation and p-VHL–mediated degradation has been extensively studied, relatively little is known about how *EPAS1* (HIF-2α) is transcriptionally regulated. Previous work identified a functional iron-responsive element (IRE) within the 5′-UTR of *EPAS1* mRNA and demonstrated that this region can be exploited as a therapeutic target using small molecules that enhance IRP1 binding to repress HIF-2α translation (49, 50). However, most reports to date have focused on such translational or posttranslational mechanisms, whereas the upstream signaling pathways and *cis*-regulatory elements that control *EPAS1* transcription remain poorly characterized. This knowledge gap has been emphasized in recently comprehensive reviews of HIF biology, which note that, while HIF target gene regulation is well understood, the transcriptional regulation of HIF-2α itself remains underexplored (51, 52). These observations underscore the need to investigate transcriptional and posttranscriptional mechanisms that may sustain HIF-2α expression independently of VHL inactivation or hypoxia signaling. Our study addresses this critical gap by uncovering 2 complementary regulatory pathways that converge on HIF-2α expression: (a) the TREX RNA export complex, which promotes *EPAS1* mRNA export and stability, thereby enhancing HIF-2α accumulation posttranscriptionally; and (b) the SOCS3/JAK1/STAT3 axis, which transcriptionally activates *EPAS1* via enhancer binding by STAT3. Together, these findings reveal a layered regulatory architecture that expands our mechanistic understanding of HIF-2α dysregulation in ccRCC and highlight previously unrecognized opportunities for therapeutic intervention.

The TREX complex, which links transcription to mRNA nuclear export, likely enhances HIF-2α function by promoting the export and stability of *EPAS1* mRNA, suggesting a previously underappreciated posttranscriptional mechanism for HIF-2α accumulation. Recent studies have implicated TREX complex components in cancer progression. For example, THOC3, a core TREX subunit, is upregulated in lung squamous cell carcinoma (LUSC) and correlates with poor patient prognosis. Functional studies show that THOC3 promotes tumor growth, migration, and glycolysis in LUSC cells, underscoring its oncogenic role (53). Similarly, THOC1 is overexpressed in multiple cancer types, including lung, colon, and ovarian tumors, and its expression in breast cancer is associated with larger tumor size and increased metastatic potential (54). Additionally, dysfunction of the TREX complex can lead to genomic instability via R-loop accumulation, a known hallmark of cancer. These findings suggest that the TREX complex not only facilitates mRNA export but also contributes more broadly to tumorigenesis through transcriptional and genomic stability regulation (55).

In contrast, SOCS3 suppresses HIF-2α expression primarily through a transcriptional mechanism. As a classical negative regulator of cytokine signaling, SOCS3 restrains basal JAK1 activity and limits downstream STAT3 activation under steady-state conditions (20, 21). In WT 786-O cells and HKC cells, STAT3 exhibited detectable basal occupancy at the enhancer located approximately 20 kb upstream of the *EPAS1* promoter, consistent with ongoing enhancer-mediated control of HIF-2α transcription. Loss of SOCS3 resulted in enhanced STAT3 activation, which was associated with increased HIF-2α transcription, whereas enforced SOCS3 expression suppressed STAT3 signaling and reduced HIF-2α levels. This SOCS3/JAK1/STAT3/HIF-2α axis represents what we believe a novel regulatory pathway that integrates cytokine signaling with enhancer-mediated transcriptional control (Figure 6J). Repression of these enhancers using CRISPRi substantially reduced HIF-2α expression and impaired tumorigenic phenotypes, confirming their functional relevance.

Our findings further extend the established link between VHL loss and dysregulated cytokine signaling in ccRCC. Previous studies have shown that VHL can suppress JAK/STAT signaling by promoting SOCS1-dependent regulation of JAK2 independently of HIF transcriptional activity (56). In contrast, our data identify SOCS3 as an additional negative regulator that constrains JAK/STAT signaling through a distinct JAK1/STAT3-dependent mechanism that directly affects HIF-2α expression. Together, these findings suggest that VHL inactivation disrupts multiple SOCS-mediated feedback loops, including SOCS1/JAK2 and SOCS3/JAK1 pathways, thereby promoting sustained JAK/STAT signaling and reinforcing HIF-2α dependency in ccRCC.

While our study highlights STAT3 as a central transcriptional regulator of *EPAS1* in ccRCC, prior work has demonstrated that additional transcription factors also directly control HIF-2α expression. For example, SP1 has been reported to bind the *EPAS1* promoter and activate HIF-2α transcription, consistent with its broader role in hypoxia-responsive gene regulation (32). In addition, E2F1 has been shown to directly bind the *EPAS1* promoter and promote HIF-2α transcription, linking cell-cycle control to HIF-2α expression (57). Similarly, the noncanonical NF-κB subunit p52 has been reported to bind the *EPAS1* promoter and induce HIF-2α transcription downstream of noncanonical NF-κB signaling (58).

Notably, both E2F1 and NF-κB p52 have been reported to interact functionally with STAT3, suggesting potential cooperation among these transcriptional programs. STAT3 has been shown to associate with RelB/p52 heterodimers to regulate gene expression in inflammatory contexts, including placental and immune signaling pathways (59). In addition, E2F1 and STAT3 have been demonstrated to physically and functionally interact on specific gene promoters, such as the *IL6* promoter in melanoma cells, where STAT3 coimmunoprecipitates with E2F1 and both factors cooperatively regulate transcription (60, 61). Together, these studies support a broader paradigm in which STAT3 signaling intersects with E2F- and NF-κB–dependent transcriptional networks in cancer.

In this context, our findings suggest that STAT3-dependent regulation of HIF-2α may not occur in isolation, but instead may be integrated with other transcriptional programs known to control *EPAS1* expression. Although our data establish STAT3-driven, enhancer-mediated regulation as a key mechanism of HIF-2α transcription in ccRCC, future studies will be required to determine whether STAT3 cooperates with E2F1, NF-κB p52, or SP1 at the *EPAS1* locus under specific oncogenic or inflammatory conditions.

Importantly, this SOCS3/JAK1/STAT3 mechanism is not limited to basal oncogenic signaling. We demonstrated that IFN-γ, a cytokine commonly secreted by activated T cells and NK cells in the tumor microenvironment (62), activated this pathway. IFN-γ stimulation led to robust STAT3 phosphorylation, increased HIF-2α expression, and upregulation of HIF-2α target genes — effects that were abrogated by the JAK1/2 inhibitor ruxolitinib. These findings directly link immune system activation to HIF-2α transcription, suggesting that cytokine-rich tumor microenvironments may inadvertently fuel HIF-2α–driven tumor progression. This cytokine-driven HIF-2α upregulation has broad implications for tumor progression and immunotherapy resistance. Chronic IFN signaling has been implicated in driving resistance to immune checkpoint blockade (ICB) by inducing multigenic immune escape programs, as shown by Benci et al. in melanoma models (63). Our findings suggest that HIF-2α may act as a downstream effector of this resistance, contributing to VEGF-mediated immunosuppression and metabolic reprogramming. Thus, targeting the JAK1/STAT3/HIF-2α axis could represent a strategy to overcome both intrinsic and immune-induced resistance. Support for this approach comes from recent clinical and preclinical studies. In non–small cell lung cancer, combining JAK inhibition with programmed death protein 1 (PD-1) blockade improved antitumor responses in immunotherapy-resistant patients (64). Similarly, a study showed that JAK inhibition restores responsiveness to nivolumab in Hodgkin lymphoma by reversing T cell exhaustion (65). Together, our findings provide a mechanistic rationale for targeting the SOCS3/JAK1/STAT3/HIF-2α axis in ccRCC. In addition to its role in oncogenic transcription, this pathway integrates immune-derived cytokine signals to modulate HIF-2α expression and tumor progression. As such, JAK/STAT inhibition offers a dual therapeutic opportunity: to suppress HIF-2α–driven tumorigenesis and to enhance the efficacy of immunotherapy by remodeling the immune-resistant tumor microenvironment. Our study thus establishes a unified framework linking inflammation, chromatin regulation, and HIF-2α activation in ccRCC and potentially other HIF-driven malignancies.

## Methods

### Sex as a biological variable.

To account for sex as a biological variable, both male and female mice were included in all in vivo studies. For orthotopic xenograft experiments and subcutaneous xenograft studies, a male-to-female ratio of 2:1 was utilized to reflect the clinical epidemiology of ccRCC, in which the incidence and mortality rates in men are approximately twice those observed in women (66).

### Orthotopic tumor xenograft.

Seven-week-old NSG mice (*n* = 8 males and *n* = 4 females per group) were used for orthotopic xenograft studies. Approximately 5 × 10^5^ viable luciferase-stable cells, either with DOX-inducible empty vector (EV) or SOCS3 expression, were resuspended in 20 μL PBS containing 50% Matrigel (Corning, 354234) and injected orthotopically into the left kidney of each mouse, as described previously (67). After injection, bioluminescence imaging was performed to confirm successful tumor implantation in the kidney. Mice were then provided Purina rodent chow no. 5001 supplemented with 2,000 ppm DOX (Research Diets) to induce target gene expression. Tumor growth was monitored weekly by bioluminescence imaging using the Spectral AMI-HTX imaging system. After the indicated duration, mice were euthanized, and tumors were harvested. Tumor weight was calculated by subtracting the weight of the right normal kidney from that of the left tumor-bearing kidney. Lungs were collected for ex vivo imaging by placing them in a 24-well plate and incubating with 300 μg/mL luciferin solution.

### Subcutaneous tumor xenograft.

Six-week-old male and female NSG mice (The Jackson Laboratory) were used for subcutaneous tumor transplantation. A total of 2 × 10^6^ cells suspended in 100 μL PBS were injected directly into the back of the skin flaps of each mouse. For cell lines with DOX-inducible vectors, 1 week after injection, mice were provided Purina rodent chow no. 5001 supplemented with 2,000 ppm DOX (Research Diets) to induce target gene expression. In the 786-O–derived xenograft model, the JAK1 inhibitor ruxolitinib was administered daily at 120 mg/kg/day via oral gavage. Tumor growth was monitored every week using digital calipers, and tumor volume was calculated using the formula: V = 0.5 × L × W², where V is the tumor volume, L is the tumor length, and W is the tumor width. Mice were euthanized at the final time point, and tumors were harvested and weighed. The maximum tumor size permitted by the ethics committee was 2 cm for a single tumor or a cumulative diameter of 3 cm for multiple tumors. Tumors exceeding these limits were not allowed.

### Cell culture.

786-O, A498, 293 T, HKC, U2OS, and Caki-1 cells were purchased from the American Type Culture Collection (ATCC); UMRC2 and UMRC6 cells were purchased from MilliporeSigma; and HK-2 cells were acquired from Peter Ly’s laboratory at UTSW. The cell lines used in this study were cultured in DMEM (Gibco, Thermo Fisher Scientific) high-glucose medium supplemented with 10% FBS and 1% penicillin/streptomycin unless otherwise indicated. The PDX cell line Xp258 was acquired from James Brugarolas’s laboratory at UT Southwestern and were grown in DMEM high-glucose supplemented with 10% FBS, 1% Pen/Strep, 1× MEM nonessential amino acids (Gibco, Thermo Fisher Scientific, 11140050), 0.01 μg/mL EGF (Bio-Techne, 236-EG), and 0.4 μg/mL hydrocortisone (STEMCELL Technologies, 07925). All cells were cultured in a humidified incubator at 37°C with 5% CO_2_. All cells were tested and confirmed to be mycoplasma free using the mycoplasma detection kit (Lonza, LT07–218) or mycoplasma elimination reagent-plasmocin (InvivoGen, ant-mpt).

### Antibodies and reagents.

Rabbit anti-SOCS3 (no. 52113), rabbit anti-VHL (no. 68547), rabbit anti-HIF1α (no. 3716), rabbit anti-HIF2α (no. 7096), rabbit anti–HIF-1β/ARNT (no. 5537), mouse anti–β-actin (no. 3700), rabbit anti-vinculin (no. 4650), mouse anti-STAT3 (no. 9139), rabbit anti–p-STAT3 (Tyr705) (no. 9145), rabbit anti-JAK1 (no. 3332), rabbit anti-JAK2 (no. 3230), rabbit anti-STAT1 (no. 14994), and rabbit anti-HA tag (no. 3724) were from Cell Signaling Technology (CST). Rabbit anti-BRD7 (51009-2-AP) and rabbit anti-SMARCD1 (10998-2-AP) were from Proteintech. The following siRNAs were from Thermo Fisher Scientific; siRNAs against STAT1 (siRNA IDs 277, 278, and 279), siRNAs against STAT3 (siRNA IDs 743, 744, and 745), siRNAs against JAK1 (siRNA IDs 7646, 7647, and 7648), and siRNAs against JAK2 (siRNA ID 607, 608, and 609).

### Plasmids.

The LentiCRISPRv2 (sgRNA/Cas9, F. Zhang, Addgene catalog no. 52961), psPAX2 (Addgene, 12260), and pMD2.G (Addgene, 12259) plasmids were used. The HIF-2α-ODDmCherry reporter was derived from the previously described HIF-1α-ODDmCherry reporter (24). HIF-2α expression was driven by the pLX304 backbone plasmid (Addgene, 25890). SOCS3 overexpression was achieved using the pInducer20 backbone (Addgene, 44012). All CRISPR/Cas9 plasmids targeting genes were constructed based on the LentiCRISPRv2 vector (Addgene, 52961). For CRISPRi stable cell lines, cells were transduced with lentivirus expressing pLV-dCas9-KRAB-PGK-HygR (Addgene, 83890), followed by treatment with the appropriate selection marker. sgRNAs were expressed using the lentiGuide-Puro vector (Addgene, 52963).

### Lentivirus production and transduction.

Lentivirus was generated by transfecting HEK293T cells (at 70%–80% confluence) with the appropriate plasmid, along with the packaging vectors psPAX2 (Addgene, 12260) and pMD2.G (Addgene, 12259). The virus supernatant was collected 48 hours after transfection, filtered through a 0.45 μm filter, and stored at –80°C. For transduction, cells were seeded to reach 30%–50% confluence and infected with 200 μL virus per well in the presence of 6–8 μg/mL polybrene to enhance infection efficiency. After 8 hours, the medium was replaced, and the cells were cultured for 1 additional day before undergoing antibiotic selection. Puromycin (2 μg/mL) was used for sgRNA/shRNA plasmid selection, blastocidin (10 μg/mL) for pLX-304 backbone plasmids, and G418 (800 μg/mL) for pInducer-20 backbone plasmids.

### CRISPR screening and analysis.

The human sgRNA library Brunello in lentiGuide-Puro (Addgene catalog 73178) was amplified according to the manual instructions. Library PCR was performed to amplify and barcode the sgRNA sequences with Illumina adaptors followed by next-generation sequencing (NGS) to check sgRNA distribution. To produce the library virus for infection, the pooled lentiviral vectors and packaging system, as described above, were applied for virus production. Clonal 786-O HRE-ODDmCherry cells were transduced with LentiCas9-BSD (Addgene no. 52962) and selected for Cas9 expression using hygromycin. A total of 3 × 10^8^ 786-O HRE-ODDmCherry cells were transduced with pooled sgRNA virus (multiplicity of infection of ~0.3), maintaining a 1,000-fold sgRNA coverage. After 24 hours, cells were treated with puromycin 2 μg/mL for 3 days. Representation was maintained throughout the screen at a minimum of 8 × 10^7^ cells. After 10 days, FACS was performed by harvesting and sorting 8 × 10^8^ cells. Cells were sorted according to the mCherry signal and kept on ice through this process to maintain stability of the reporter. Ten percent high mCherry signal cells and 10% low mCherry signal cells were harvested (8 × 10^7^ cells in each group), making the final coverage approximately 1,000 fold. Genomic DNA was extracted using a Gentra Puregene Core kit (Qiagen). Lentiviral sgRNA inserts were amplified in a 2-step PCR (with Illumina adapters added on the second PCR). All the primers used are listed in Supplemental Table 3. Amplicon sequencing was performed on an Illumina NextSeq 2000 sequencer. sgRNA sequences were extracted from FASTQ files and aligned to the sgRNA sequences of the CRISPR screen library. Read counts for each sgRNA were compared between conditions, and Benjamini-Hochberg FDRs for each gene were calculated using MAGeCK (Supplemental Tables 1 and 2).

### RT-qPCR.

For RT-qPCR, total RNA was extracted using the RNeasy Mini Kit, and cDNA synthesis was performed using the iScript cDNA Synthesis Kit (Bio-rad, 1708841BUN). RT-qPCR was conducted using the CFX384 Real-Time PCR System (Bio-Rad). Relative amplicon expression was calculated by applying the 2^–ΔΔCt^ method. The sequence of RT-qPCR primers can be found in Supplemental Table 4.

### 3D colony formation assay.

For the 3D colony formation assay, approximately 20,000 cells were seeded in the upper layer agar, cultured for 4 weeks, and stained with 100 μg/mL iodonitrotetrazoliuim chloride solution. The quantification of colonies was performed using ImageJ software (NIH).

### ChIP-seq and analyses.

ChIP was performed using the SimpleChIP Enzymatic Chromatin IP Kit (CST, no. 9003). Briefly, cells were cross-linked with 1% formaldehyde, followed by treatment with 10× glycine to quench the reaction. Cells were lysed in lysis buffer (10 mM Tris-HCl pH 8.0, 100 mM NaCl, 1 mM EDTA, 0.5 mM EGTA, 1% Triton X-100) and sonicated with a BioRuptor to shear chromatin. Immunoprecipitation was performed by incubating chromatin with the following specific antibodies: STAT3 (CST, 9139), H3K4me1 (Abcam, ab8895), H3K4me3 (Abcam, ab8580), and H3K27ac (Abcam, ab4729) using protein G magnetic beads at 4°C overnight with rotation. The samples were washed, collected using a magnetic stand, and eluted for crosslink reversal. Proteinase K (MilliporeSigma, 3115828001) was used for protein degradation and RNAse A (MilliporeSigma, 70856-3) for RNA degradation. DNA was purified using the Qiagen PCR Purification Kit (Qiagen, 28106). For ChIP-seq, libraries were prepared using the NEBNext Ultra II DNA Library Prep Kit (NEB, E7645S) and sequenced at the McDermott Center Next Generation Sequencing Core at UT Southwestern on Illumina Novaseq platform (HKC data) and NEXTseq 2000 (786-O data). ChIP-seq data for HKC (STAT3, H3K4me1, H3K4me3, H3K27ac, input control) and 786-O (STAT3, IgG control) were processed with nf-core/chipseq1,2 version 2.1.0 using the hg38 reference and GENCODE version 43 gene annotations. Default parameters were used except that MACS3 additionally specified “--keep-dup all --nomodel --extsize 147” for peak calling. Additional raw ChIP-seq data in 786-O (H3K4me1, H3K27ac, H3K27me3, HIF1B, HIF2A) were obtained from GSE1020953 and GSE980124 and reprocessed in an identical fashion. Data were visualized using plotgardener5 version 1.6.4 in R version 4.3.0. ChIP-seq reads for patients’ tissue were obtained from GSE86095 and aligned to the hg38 reference genome with Bowtie2 version 2.3.4.3 (68). PCR duplicates were filtered out using Picard version 2.20.3 (http://broadinstitute.github.io/picard). Peaks were detected with MACS2 version 2.1.0 (69) under default settings, using input DNA as the control. Finally, average signal profiles and heatmaps were produced with deepTools version 2.4.2 (70).

### Patient tissue RNA-seq analysis.

Patient tissue RNA-seq data are from GSE86095, and raw RNA-seq reads were first assessed for initial overall sequencing quality control using FastQC. (version 0.11.8, http://www.bioinformatics.babraham.ac.uk/projects/fastqc). Clean sequences were mapped to the human hg38 genome using STAR version 2.7.3a (71). Transcript abundance for each sample was quantified with RSEM version 1.3.2 (72) under default settings.

### HiChIP, data analysis, and loop calling.

The HiChIP experiment was outsourced to Dovetail Genomics (Cantata Bio) and performed per their established protocol. Briefly, approximately 10 million 786-O cells were collected, and chromatin was subsequently fixed with 3 mM disuccinimidyl glutarate (DSG), followed by another fixation with 1% formaldehyde. The fixed chromatin was digested in situ with micrococcal nuclease (MNase) and then extracted with RIPA lysis buffer. Chromatin fragments were incubated with anti-H3K27ac antibody (CST, 8173) overnight at 4°C for ChIP. The antibody-protein-DNA complex was pulled down with protein A/G–coated magnetic beads. Chromatin ends were repaired and ligated to a biotinylated bridge adapter followed by proximity ligation of adapter-containing ends. After proximity ligation, crosslinks were reversed, and the DNA was purified from proteins and converted into a sequencing library. The sequencing library was generated using Illumina-compatible adapters. Biotin-containing fragments were isolated using streptavidin beads before PCR enrichment of the library. The library was sequenced on the Illumina NovaSeq platform with paired-end sequencing at 300 million reads per sample.

H3K27ac HiChIP data were first processed with nf-core/hic2,6 using the pairtools processing mode (available from https://github.com/nservant/nf-core-hic/tree/dev) with “--no_digestion --min_cis_dist 1000” and bin sizes ranging from 1 kb to 1 Mb. The 5 kb contact maps were then used as input for FitHiChIP7 version 11.0 alongside H3K27ac ChIP-seq consensus peaks from GSE102095 as identified above, running in Peak-to-Peak mode with the following parameters: BINSIZE=5000, LowDistThr=5000, UppDistThr=2000000, UseP2PBackgrnd=1, BiasType=2, MergeInt=1, QVALUE=0.1. 

### Statistics.

Statistical analyses were performed with GraphPad Prism 9.0 (GraphPad Software). For experiments comparing 2 sets of data, an unpaired, 2-tailed Student’s *t* test was used. A 1- or 2-way ANOVA was used for multiple comparisons. All other data represent the mean ± SEM from at least 3 independent experiments. A *P* value of less than 0.05 was considered significant.

### Study approval.

All procedures involving mice were approved by the IACUC of the University of Texas Southwestern Medical Center (approval 2019-102794).

### Data availability.

Raw and processed ChIP-seq and HiChIP data have been deposited in the Gene Expression Omnibus (GEO) database (GEO GSE303721). Values for all data points are available in the Supporting Data Values file. All other data and reagents relevant to the current study are available on reasonable request, excluding confidential patient identity information.

## Author contributions

JF and QZ conceived of and designed the study. JF and TW acquired data. JF, JMS, YG, XB, and QZ analyzed and interpreted data. JF, JB, JMS, JAN, and QZ wrote and reviewed and/or revised the manuscript. JF, LH, CZ, JZ, HL, QL, CL, RM, YA, JB, and QZ performed administrative, technical, and material support duties.

## Conflict of interest

QZ received consultation fees from Exelixis.

## Funding support

This work is the result of NIH funding, in whole or in part, and is subject to the NIH Public Access Policy. Through acceptance of this federal funding, the NIH has been given a right to make the work publicly available in PubMed Central.

- DoD Kidney Cancer Research Program (KC240077, to JF).
- Cancer Prevention and Research Institute of Texas (CPRIT) (RR190058, to QZ).
- UTSW SPORE grant P50CA196516 (to JB).

## Supplementary Material

Supplemental data

Unedited blot and gel images

Supplemental tables 1-4

Supporting data values

## Version 1 03/24/2026

In-Press Preview

## Version 2 05/15/2026

Electronic publication

## Version 3 09/15/2026

Corrected Supplemental Figure 2G and legend only for Figure 1I and J
