# Corrigendum to Genome-wide CRISPR screen identifies a cytokine-enhancer circuit driving HIF-2α activation in renal cancer

**DOI:** 10.1172/JCI211482

**Published:** 2026-09-15

**Authors:** Jun Fang, Jeremy M. Simon, Tao Wang, Yunpeng Gao, Xianju Bi, Lianxin Hu, Chengheng Liao, Cheng Zhang, Yayoi Adachi, Jin Zhou, Hongyi Liu, Qian Liang, James A. Nathan, Ram Mani, James Brugarolas, Qing Zhang

Original citation: *J Clin Invest*. 2025;136(10):e201639. https://doi.org/10.1172/JCI201639

Citation for this corrigendum: *J Clin Invest*. 2026;136(18):e211482. https://doi.org/10.1172/JCI211482

After publication of this article, the authors became aware of the following errors: A portion of the qPCR data was incorrectly duplicated in Supplementary Figure 2G. The error is isolated to the A498 dataset in Supplementary Figure 2G. Also, we identified a typographical error in the legend for Figure 1 regarding the reference to panels (I and J). The correct figure legend is shown below. The HTML and PDF versions of the article, including Supporting data values, have been updated online.

The authors regret the errors.

**Figure 1. FACS-based whole-genome CRISPR screen identifies HIF-2α positive regulators.** (**A**) Schematic of the HRE- HIF-2α ODD mCherry reporter construct. (**B**) Diagram of the loss-of-function, genome-wide screen using the human lentiviral sgRNA library (Brunello) in the HIF-2α reporter 786-O cell line. Ten percent of cells with either low or high signal intensity were sorted for NGS to identify factors that regulate HIF-2α activity. (**C**) Top genes identified for positive regulation of HIF-2α activity versus their log_10_ robust rank aggregation (RRA) scores in the genome-wide loss-of-function screen. Red circles are regulators reported before and green circles are genes related to the mRNA export pathway. (**D**) Distribution of sgRNA log fold change (LFC) comparing the mCherry-low group with the presorted group (positive regulators). Red bars represent 4 individual sgRNAs for the indicated genes. (**E**–**H**) Immunoblot analysis of cells with TREX component KOs. (**E** and **F**) 786-O and A498 ccRCC cell lines. (**G** and **H**) HKC and HK2 epithelial cell lines were treated with 10 μM MG132 for 6 hours prior to harvesting. (**I**)FACS-based analysis of HIF-2α activity in 786-O HIF-2α reporter cells following KO of RNA export pathway components. Each gene was targeted with 2 independent sgRNAs, and HIF-2α sgRNAs were used as positive controls. (**J** and **K**) Representative soft agar growth (**J**) and corresponding quantification (*n* = 3) (**K**) of 786-O, A498, and HKC cells expressing control (sgCtrl) or sgRNAs targeting the TREX components THOC2 and ALYREF. Scale bars: 1 mm. (**L** and **M**) Representative soft agar growth (**L**) and corresponding quantification (*n* = 3) (**M**) of 786-O cells with EV or HIF-2α overexpression that were then transduced with sgRNAs targeting TREX components or control. *P < 0.05, **P < 0.01, ***P < 0.001, and ****P < 0.0001, by 2-way ANOVA followed by Tukey’s multiple-comparison test (**J**) or multiple, unpaired *t* test (**L**). Data show the mean ± SEM.

## Version 1 09/15/2026

Electronic publication

