# Supplementary figures and images for: Genome-wide CRISPR screen identifies a cytokine-enhancer circuit driving HIF-2α activation in renal cancer
Source: J Clin Invest. 2026 Mar 24;136(10):e201639. doi: 10.1172/JCI201639 (PMC13178659; doi:10.1172/JCI201639)

Supplement Figure 1

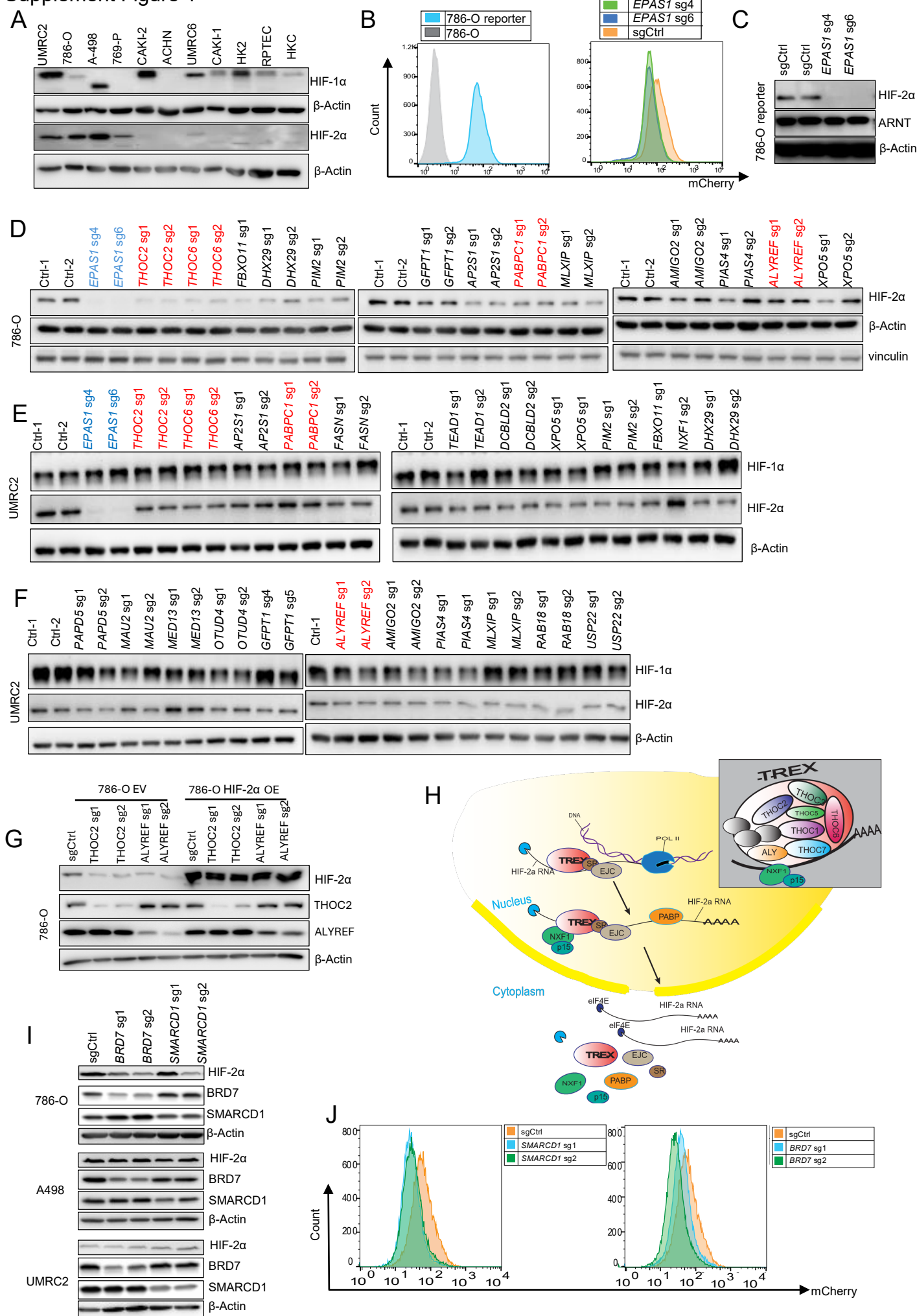

Supplement Figure 2

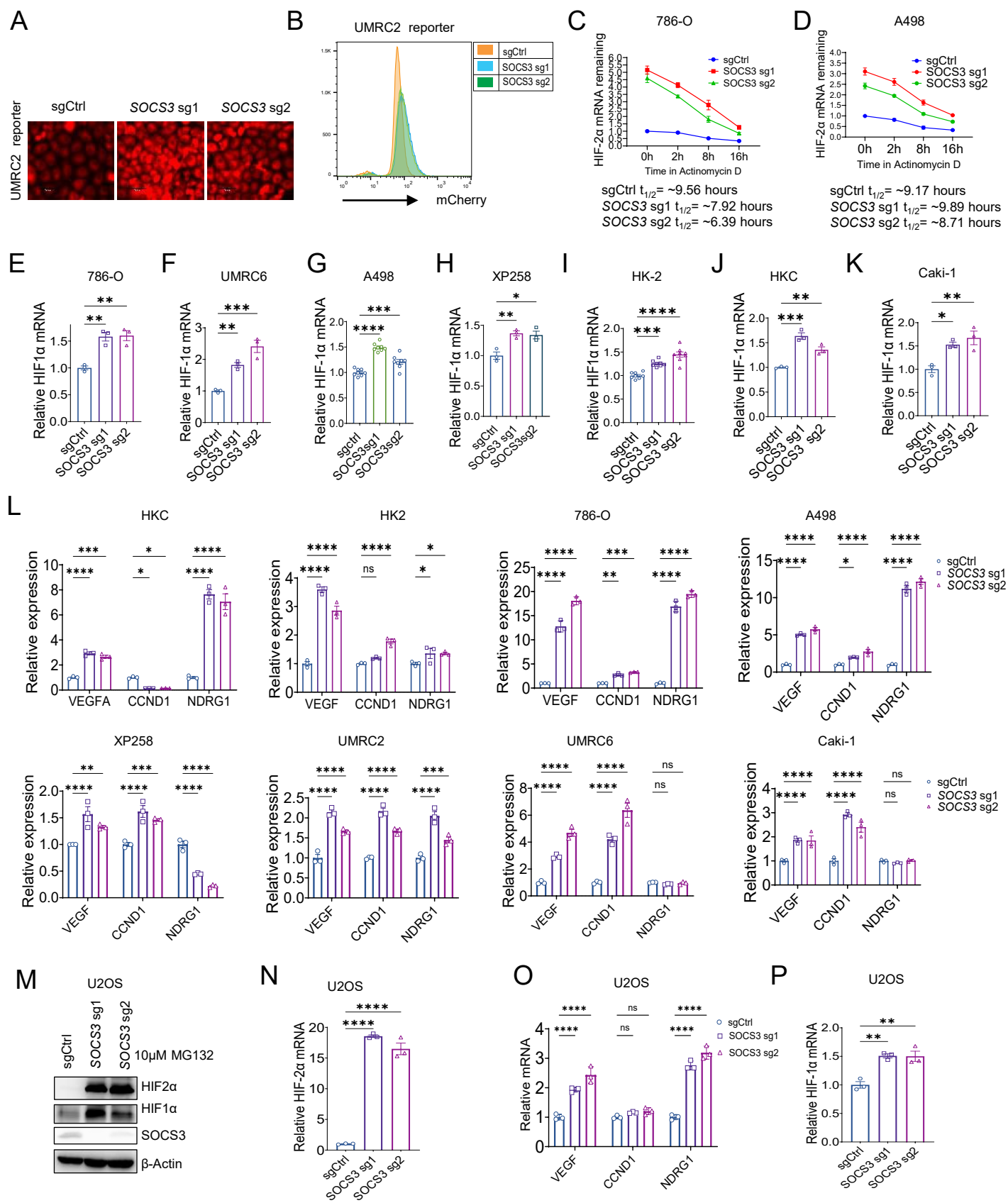

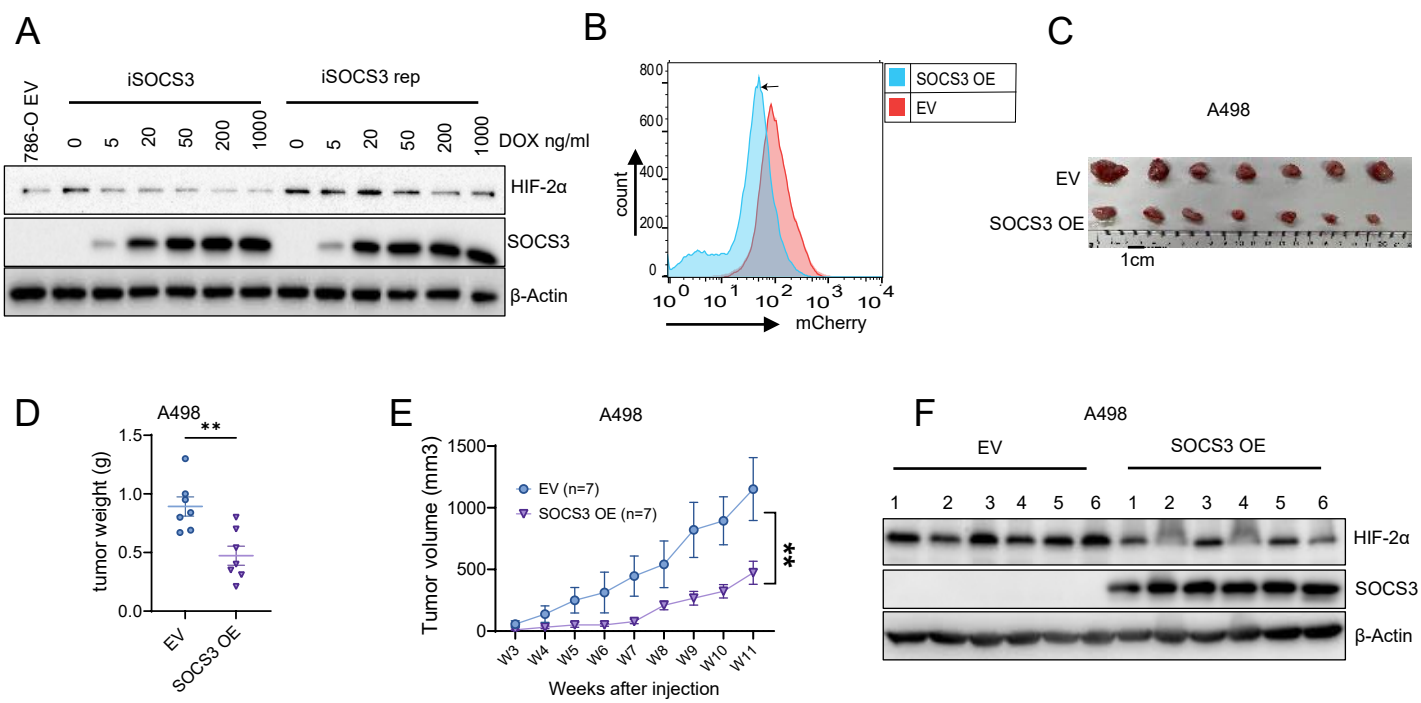

Supplement Figure 4

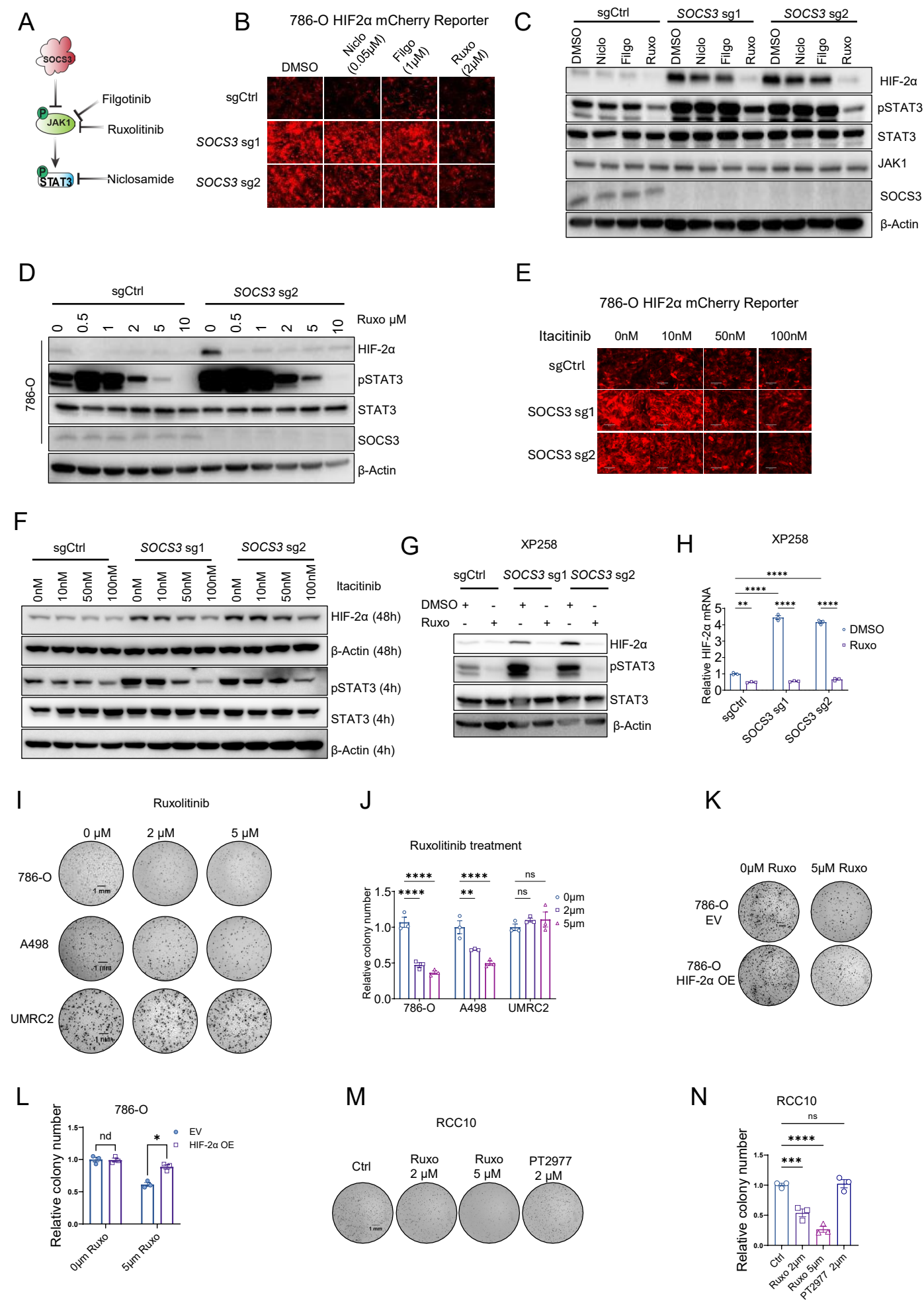

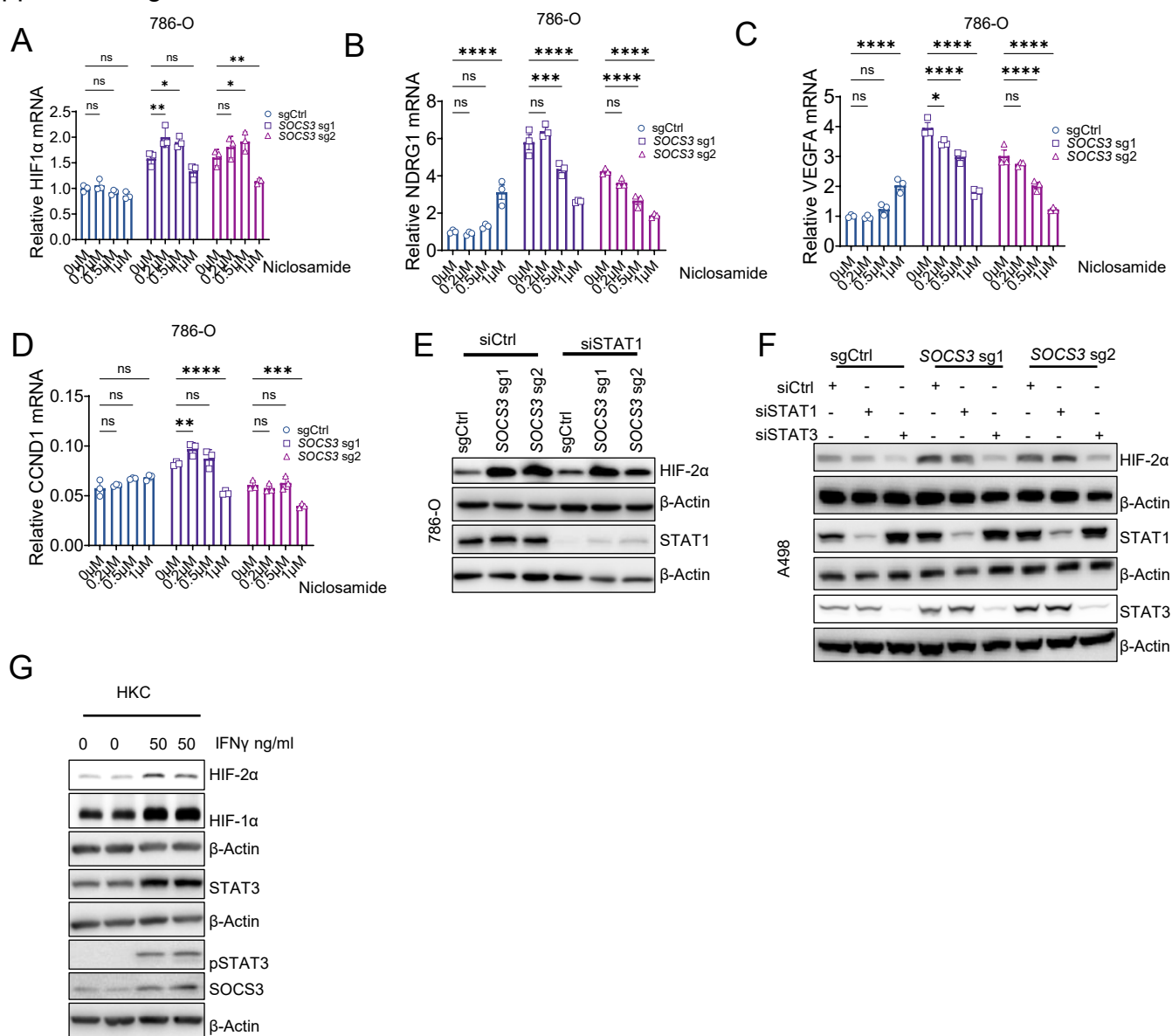

A

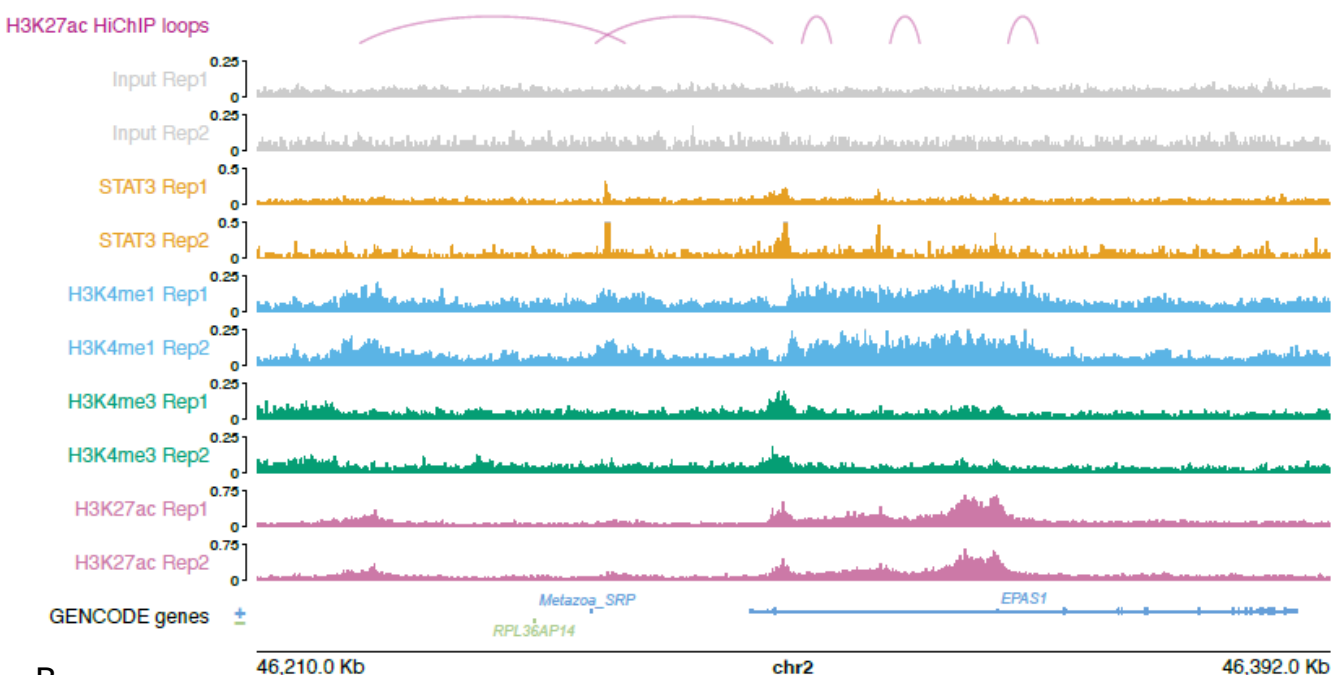

B

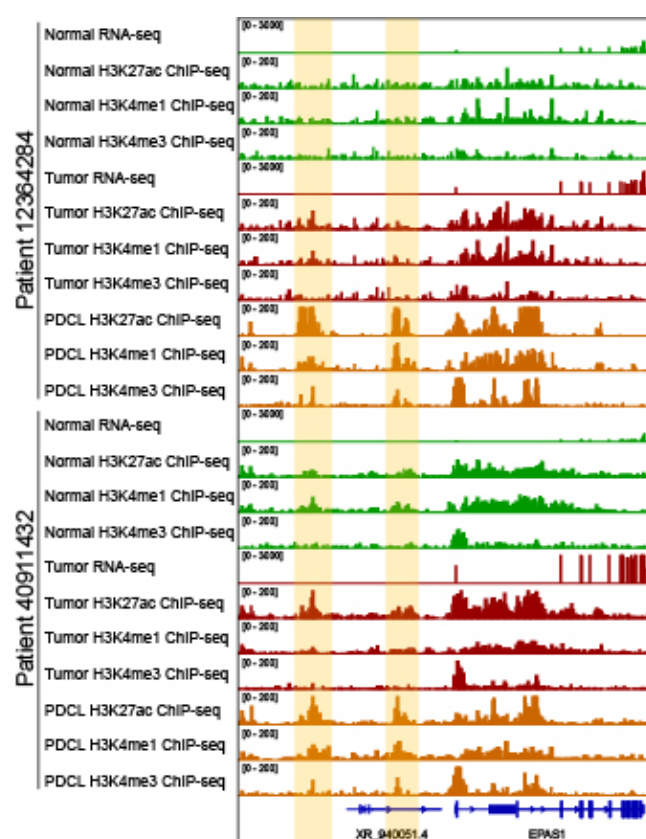

Supplement: Supplemental data [file jci-136-201639-s007.pdf]
