# Supplementary material for: Genome-wide CRISPR screen identifies a cytokine-enhancer circuit driving HIF-2α activation in renal cancer
Source: J Clin Invest. 2026 Mar 24;136(10):e201639. doi: 10.1172/JCI201639 (PMC13178659; doi:10.1172/JCI201639)

Full unedited blot/gel for Figure 1E, 1F, 1G and 1H

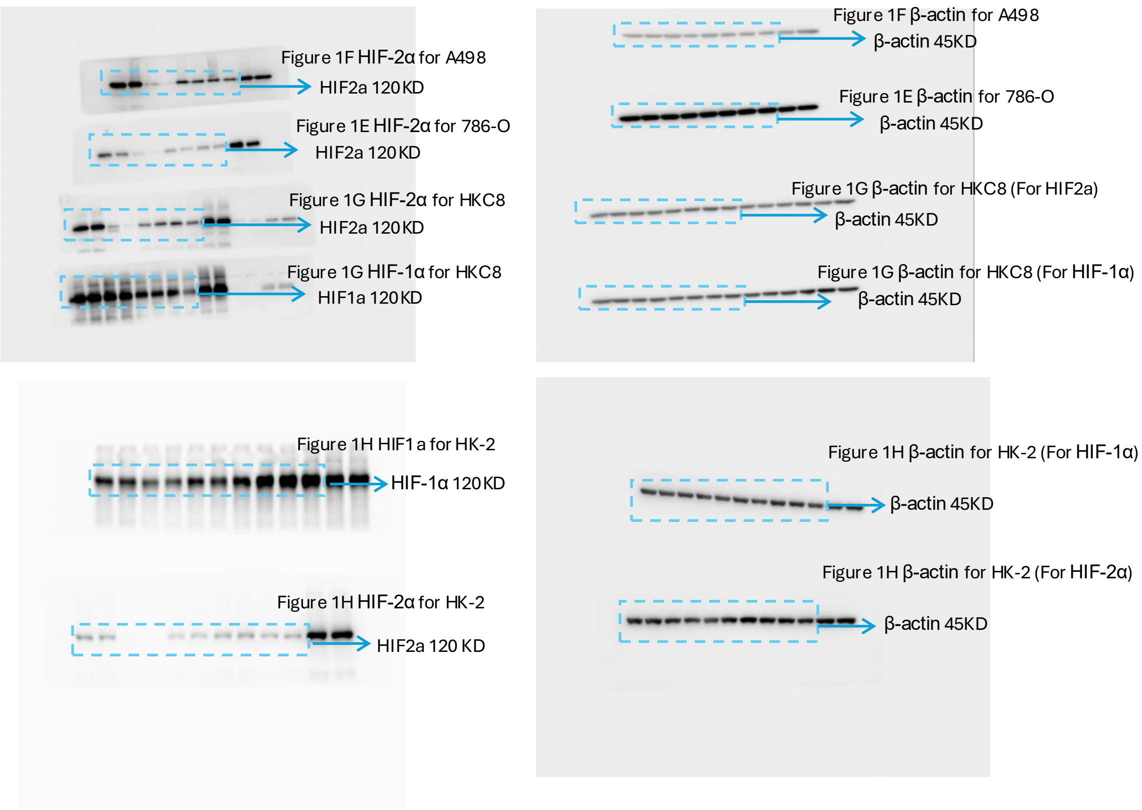

Full unedited blot/gel for Figure S1A

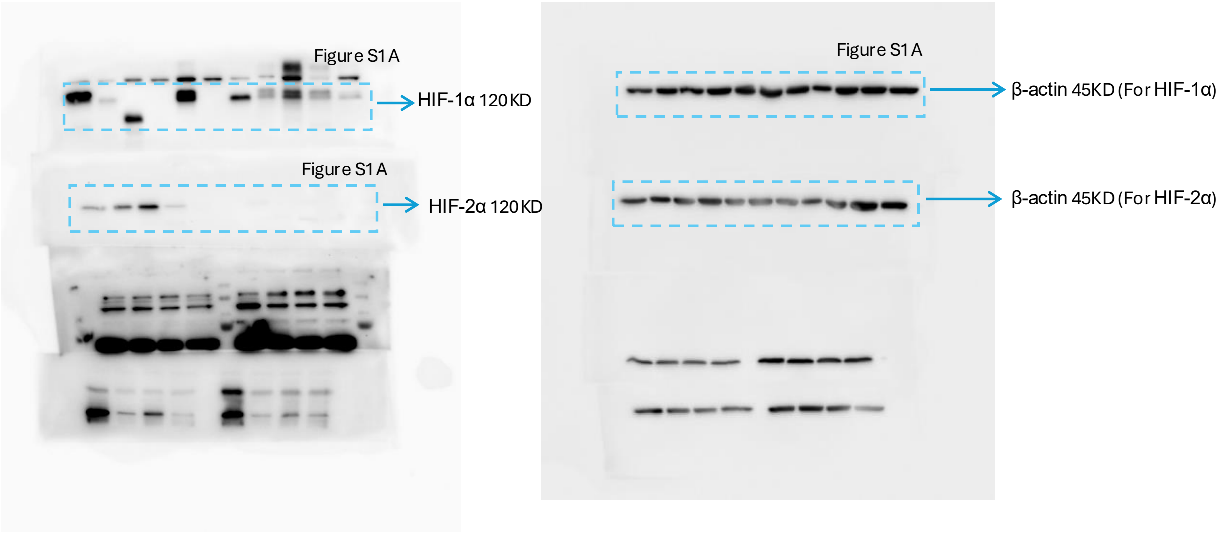

Full unedited blot/gel for Figure S1C

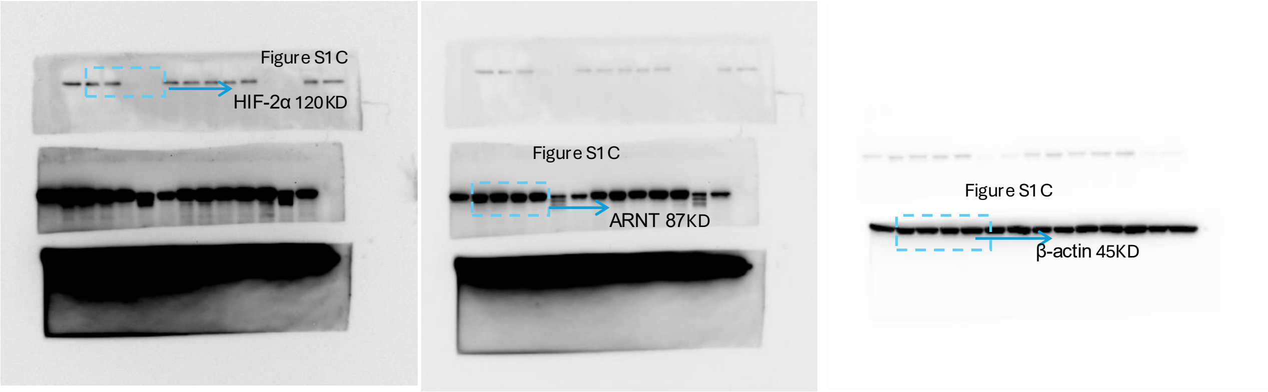

Full unedited blot/gel for Figure S1D

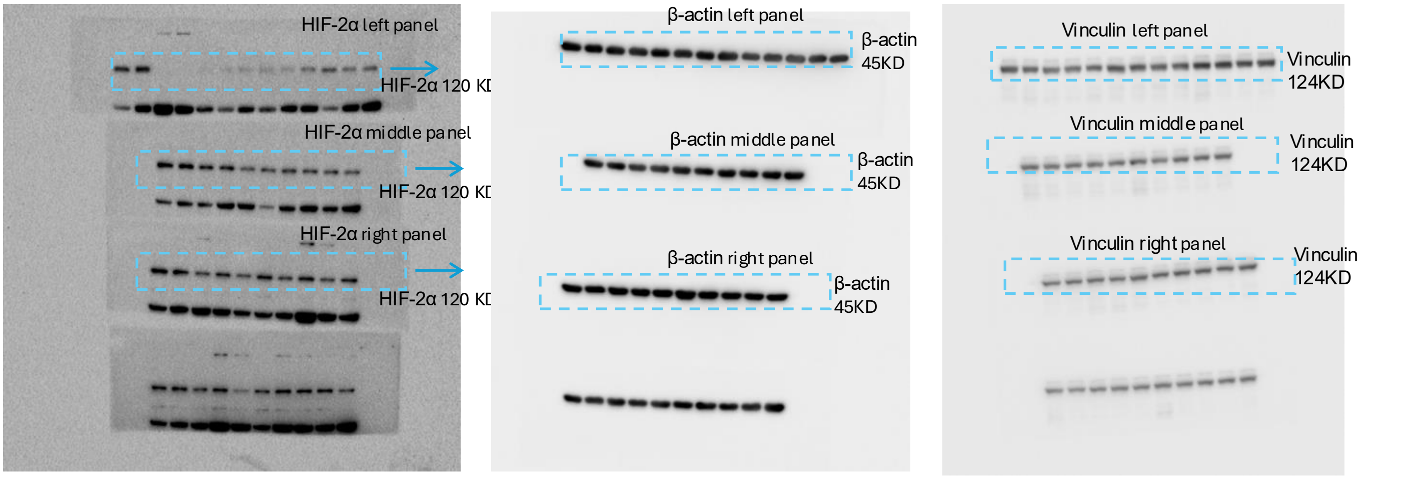

Full unedited blot/gel for Figure S1Eand S1F

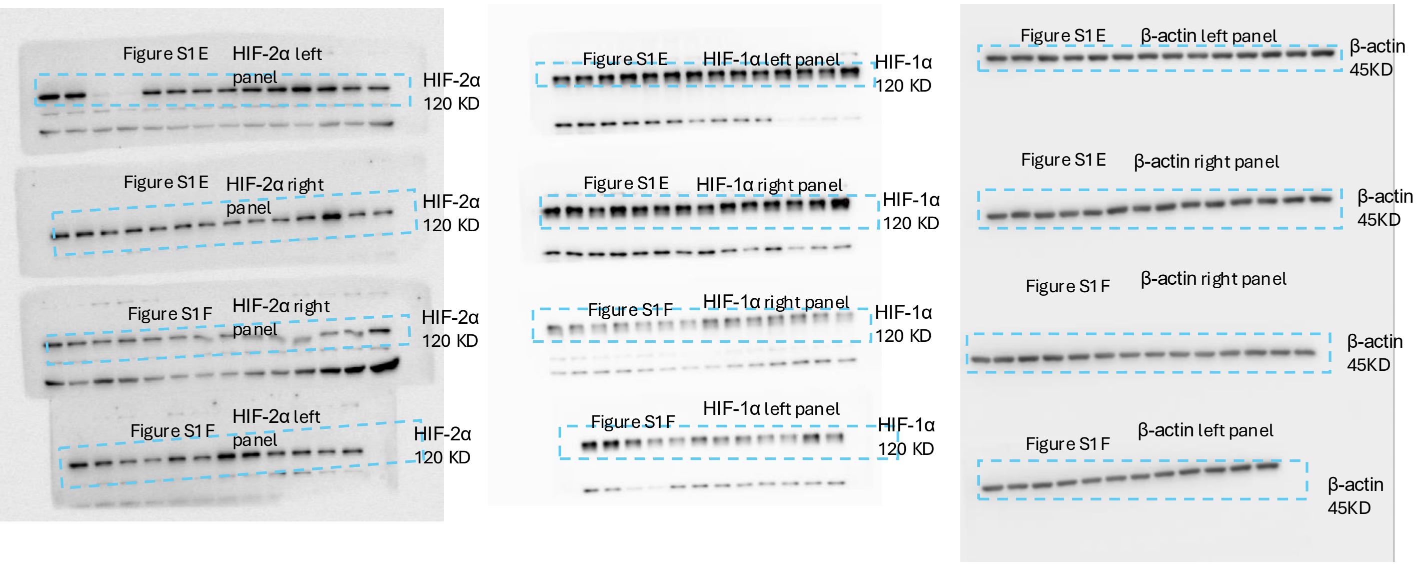

Full unedited blot/gel for Figure S1G

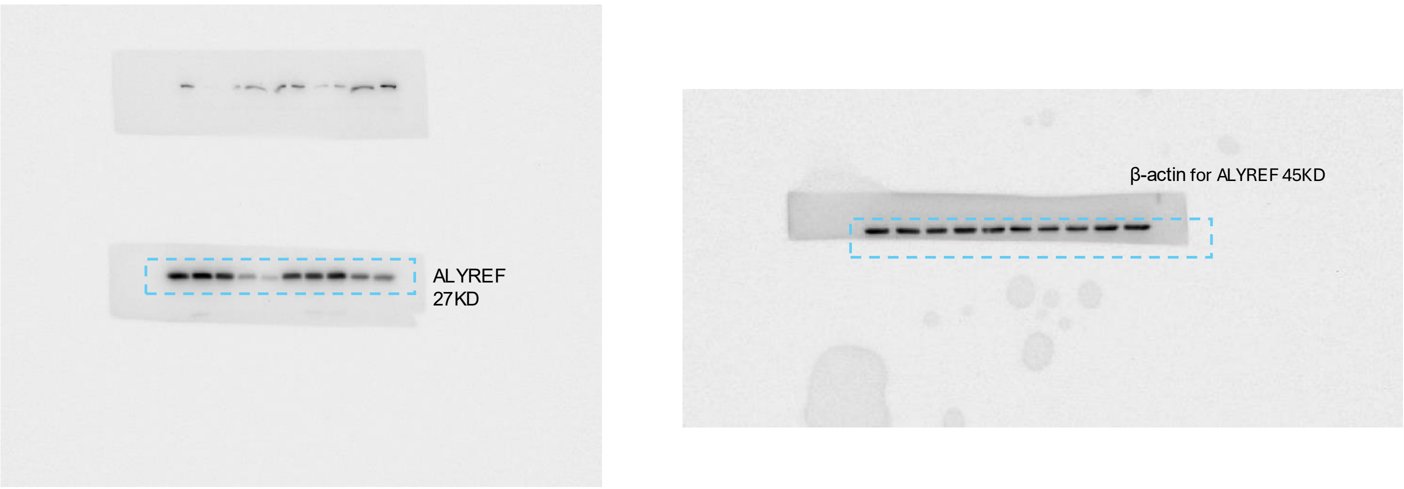

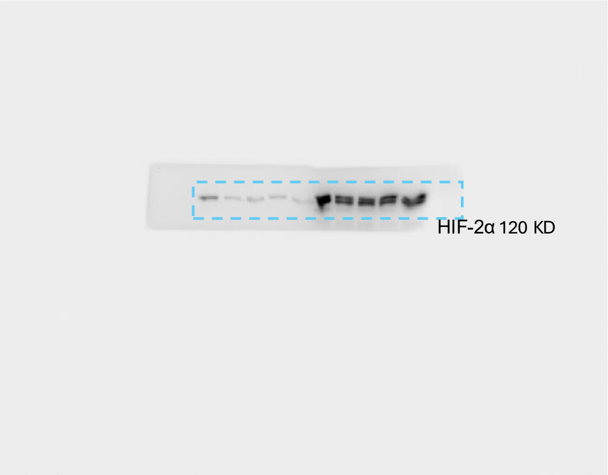

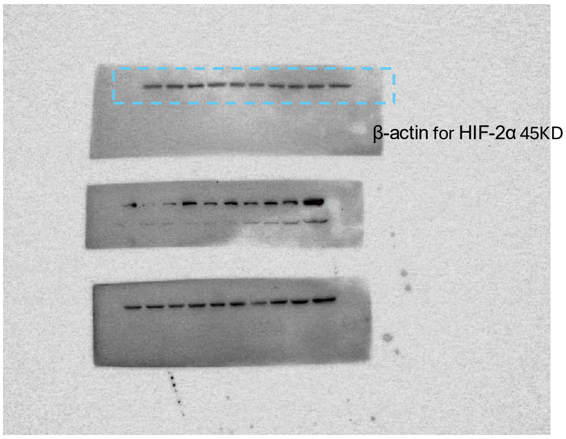

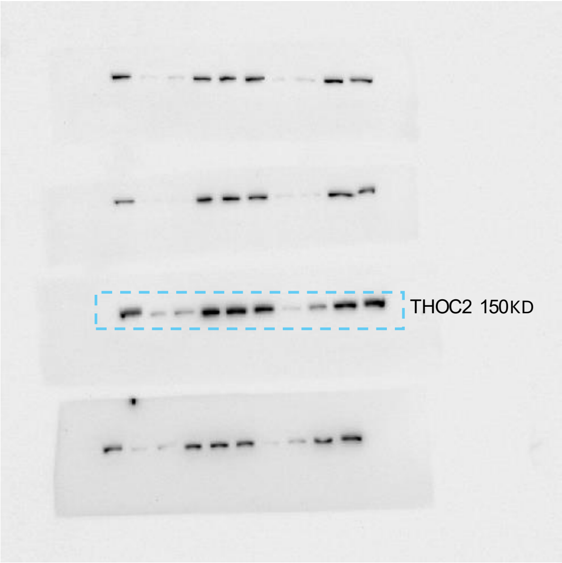

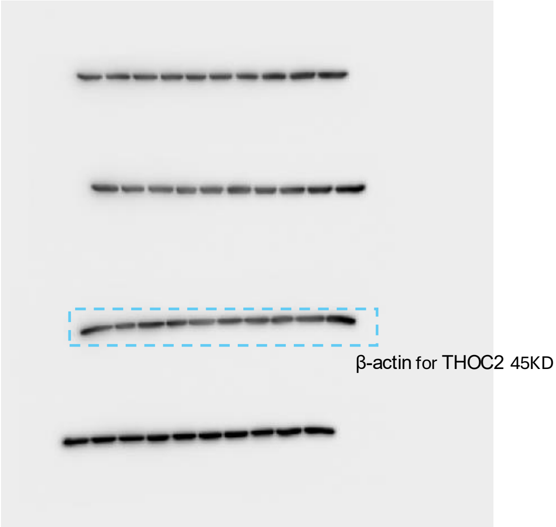

Full unedited blot/gel for Figure S1I

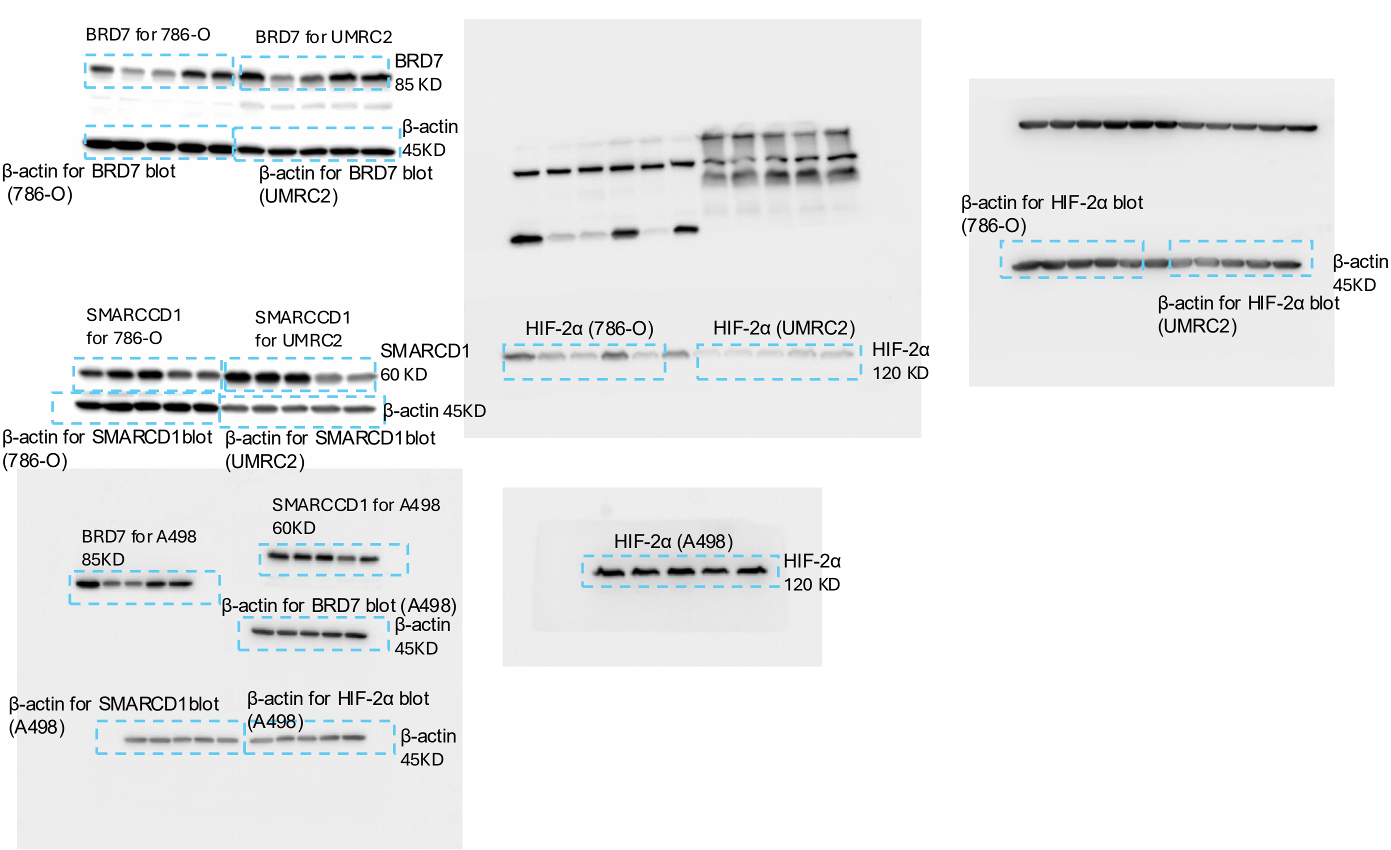

Full unedited blot/gel for Figure 2E

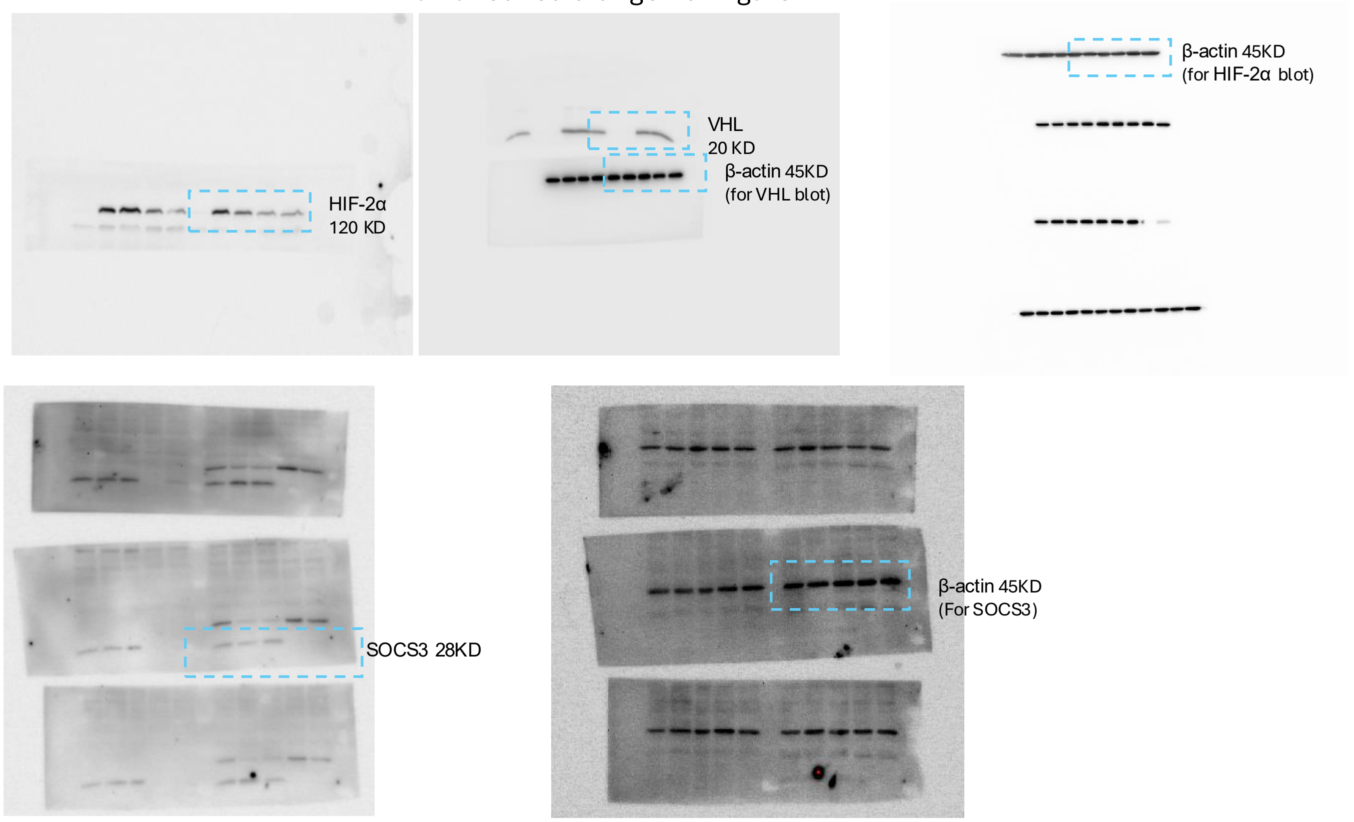

Full unedited blot/gel for Figure 2F

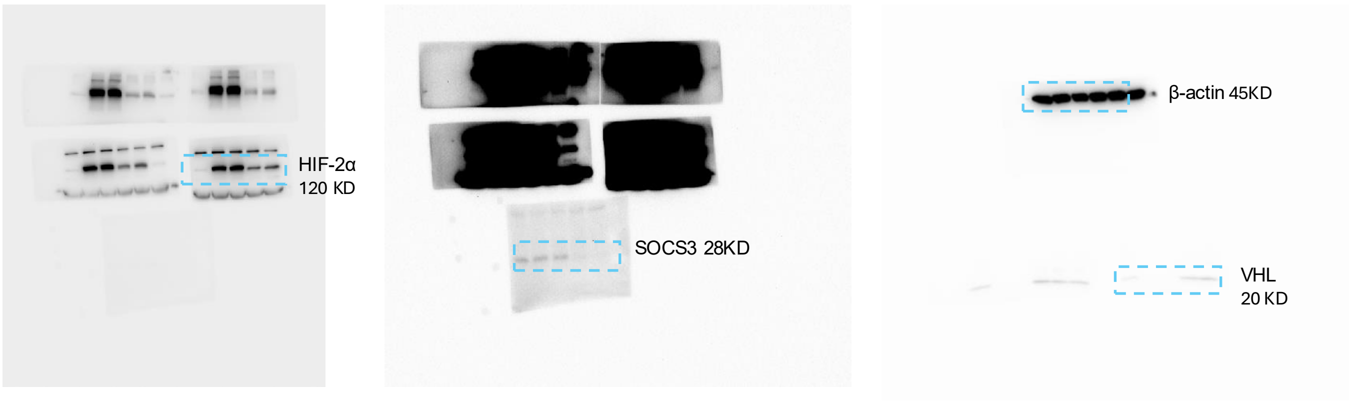

Full unedited blot/gel for Figure 2G

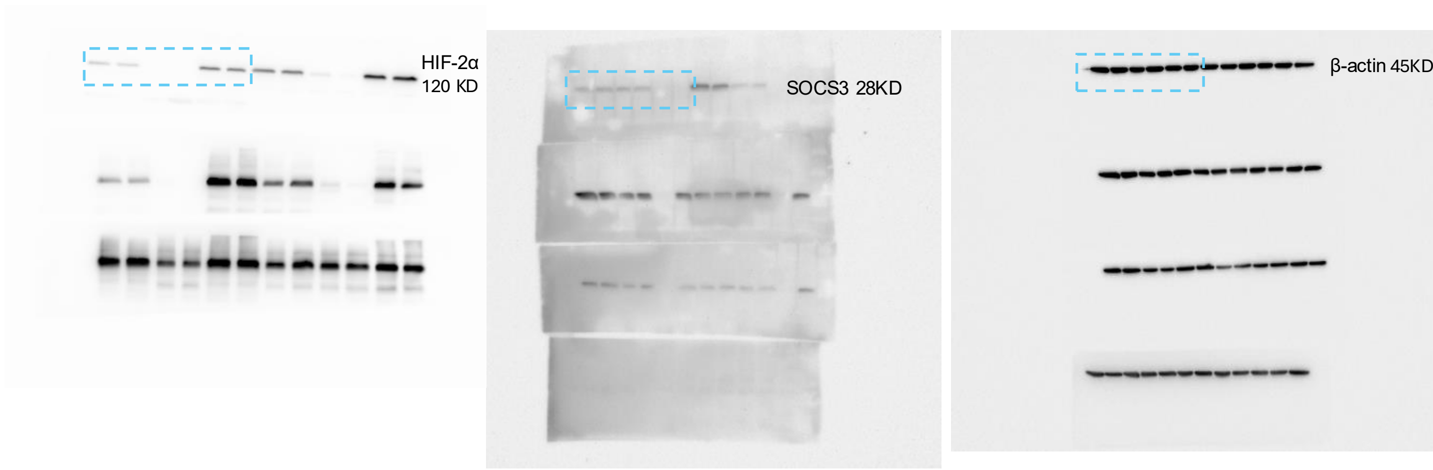

Full unedited blot/gel for Figure 2H

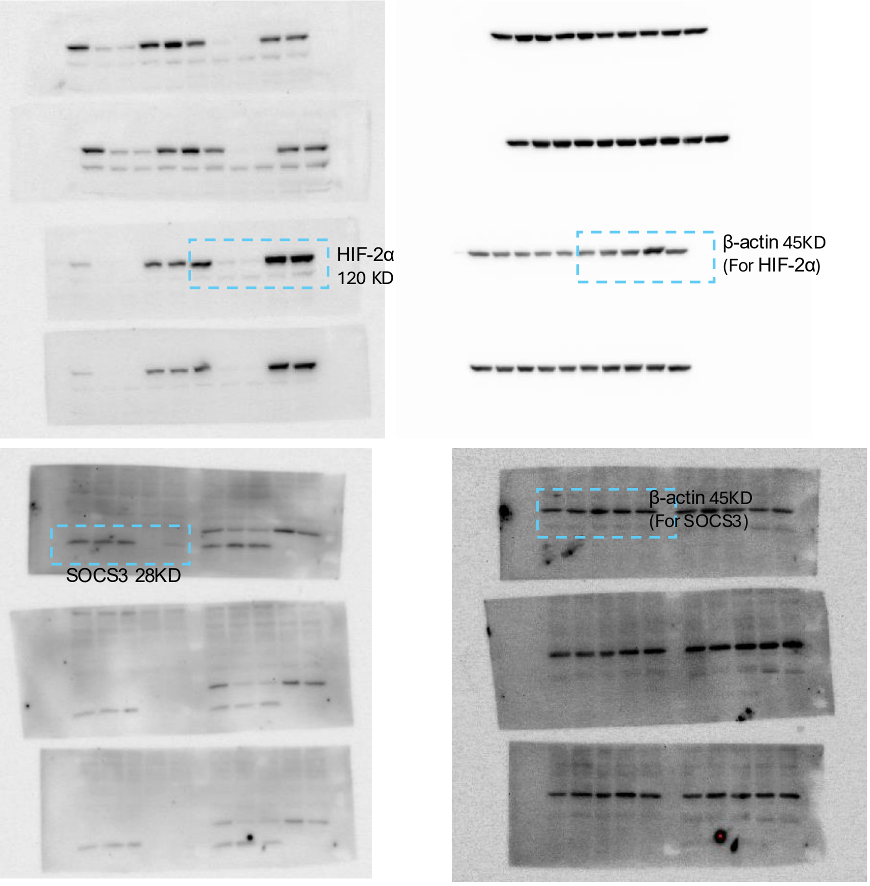

Full unedited blot/gel for Figure 2I

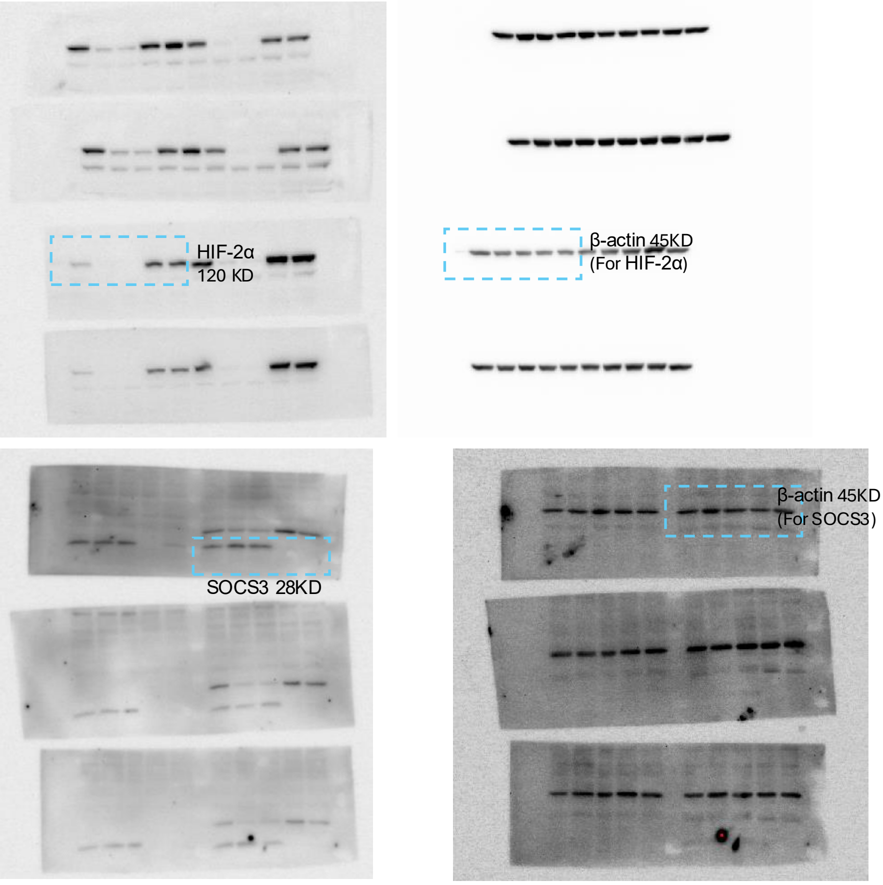

Full unedited blot/gel for Figure 2J

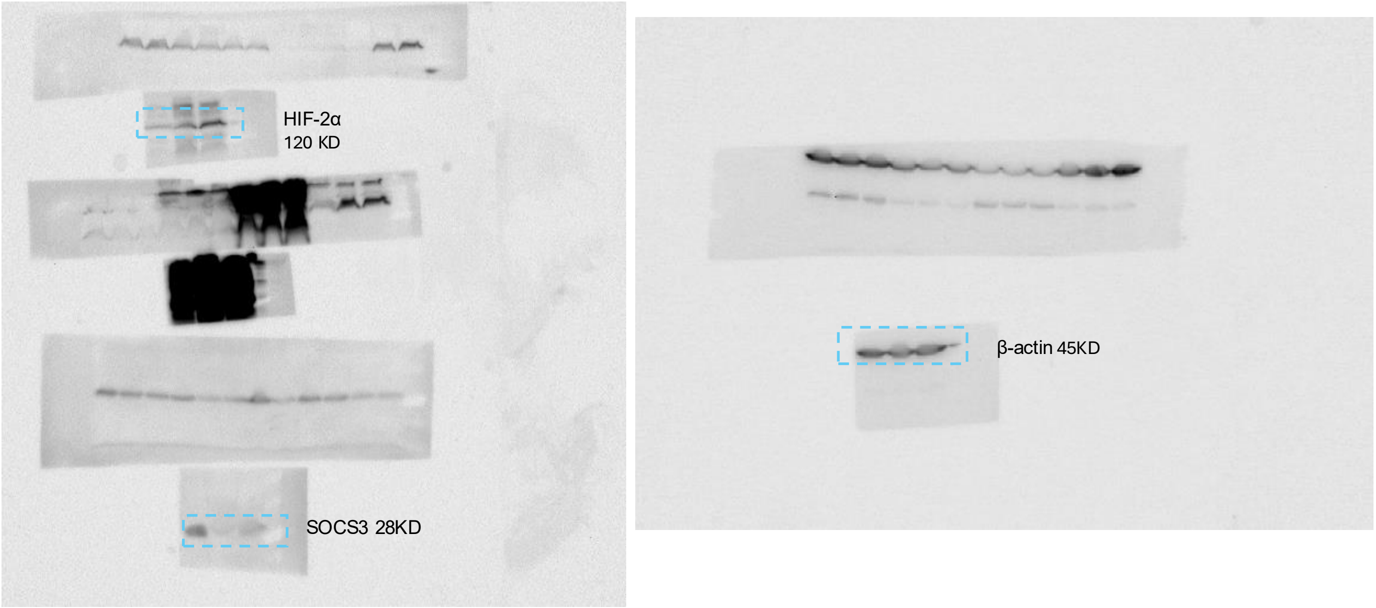

Full unedited blot/gel for Figure 2K

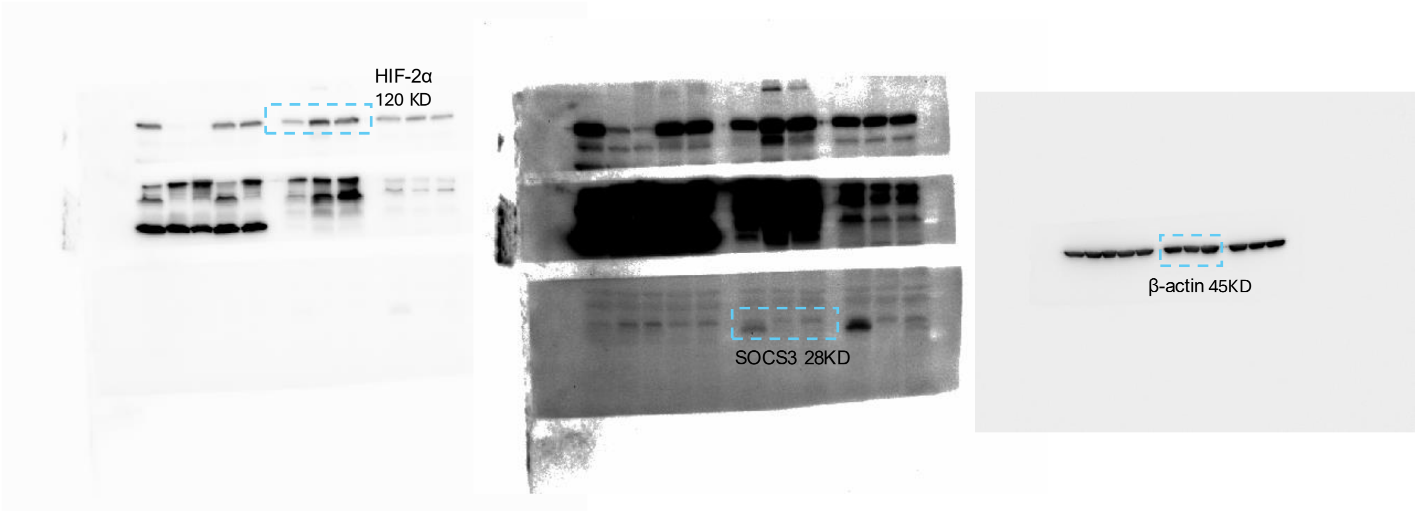

Full unedited blot/gel for Figure S2M

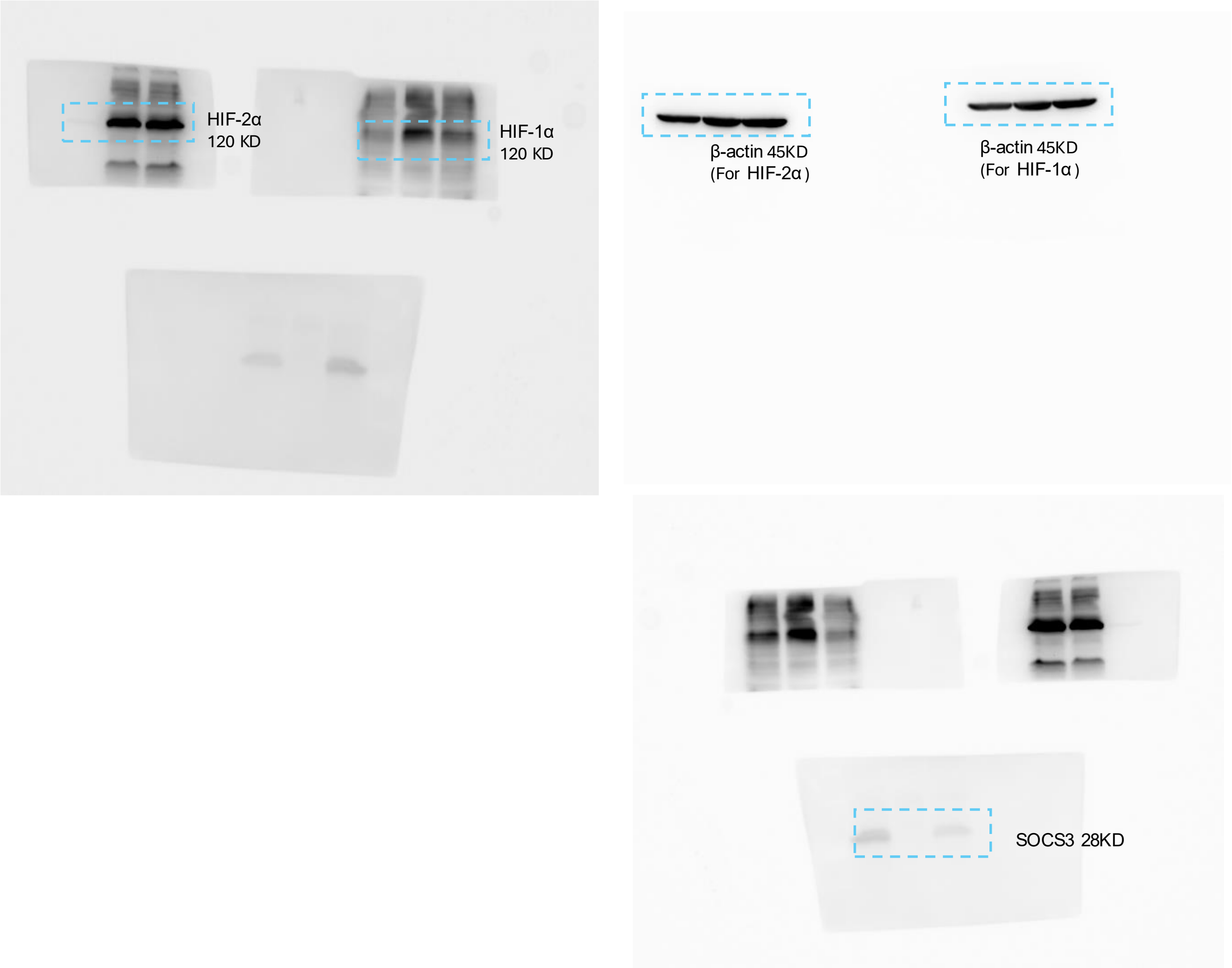

Full unedited blot/gel for Figure 3A

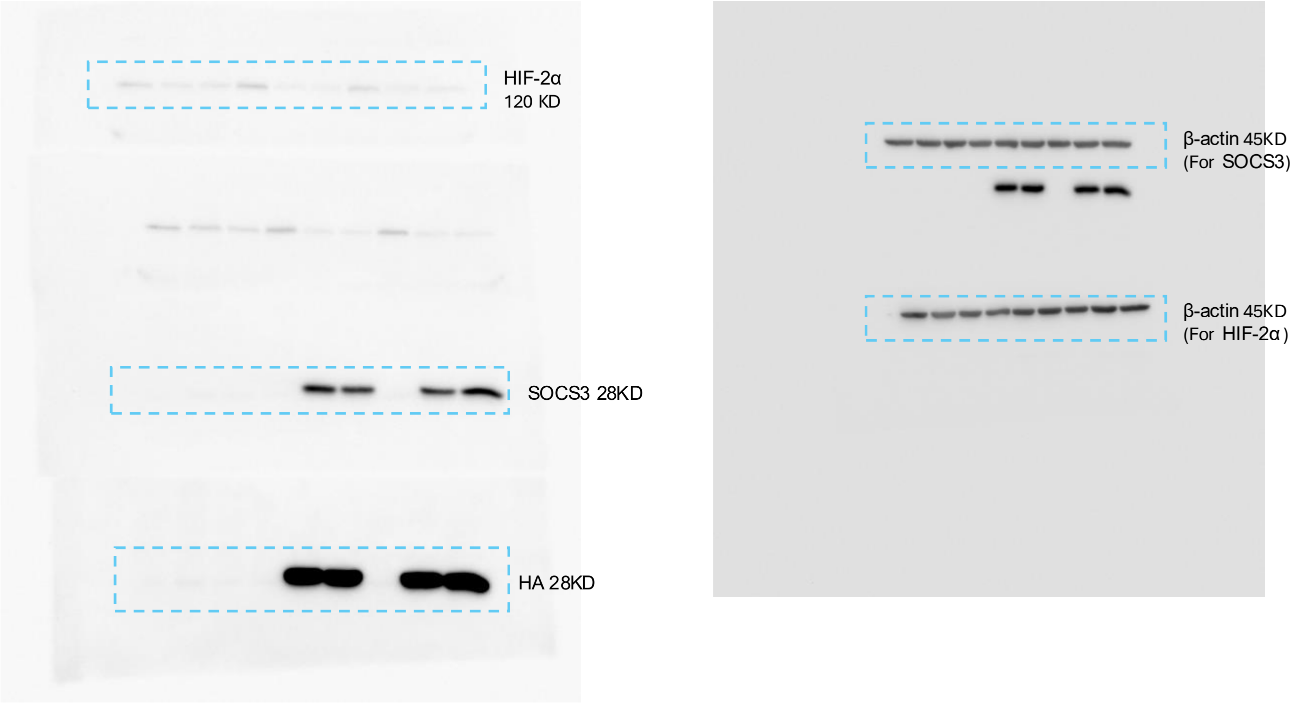

Full unedited blot/gel for Figure 3B

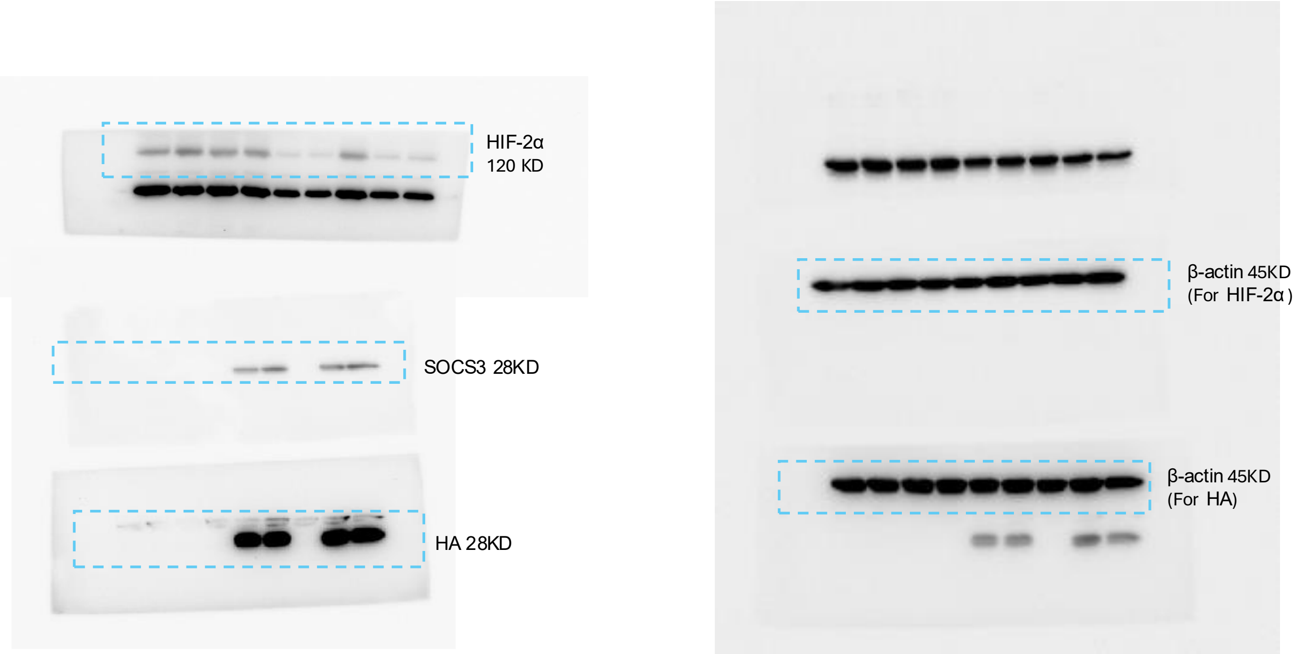

Full unedited blot/gel for Figure 3E

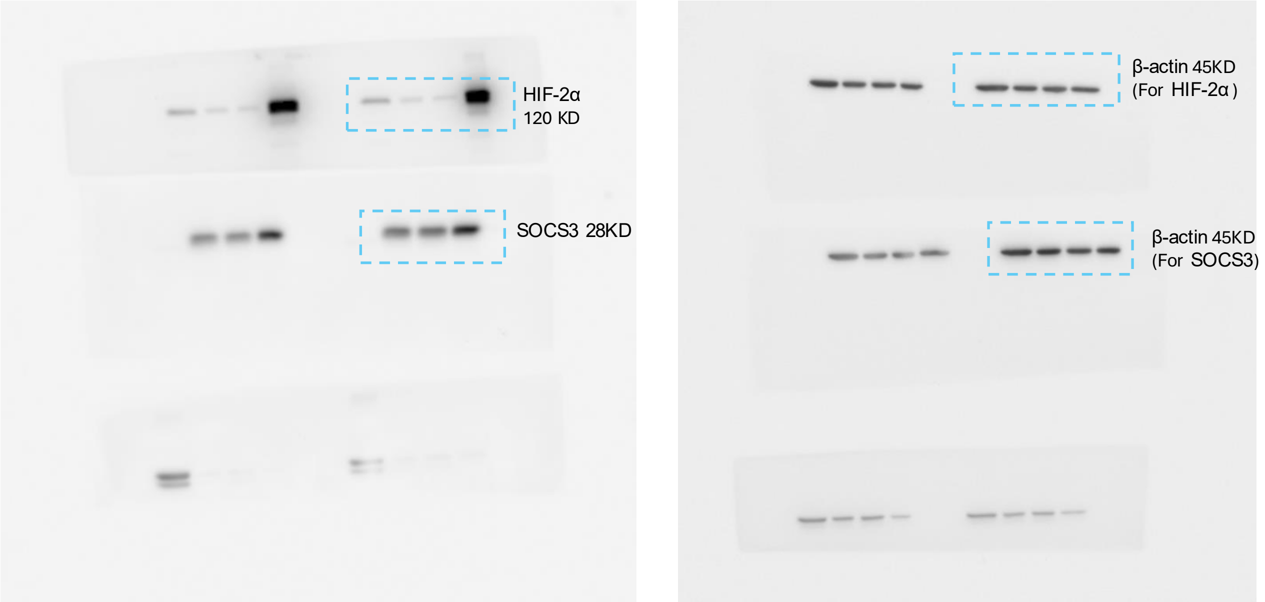

Full unedited blot/gel for Figure 3F

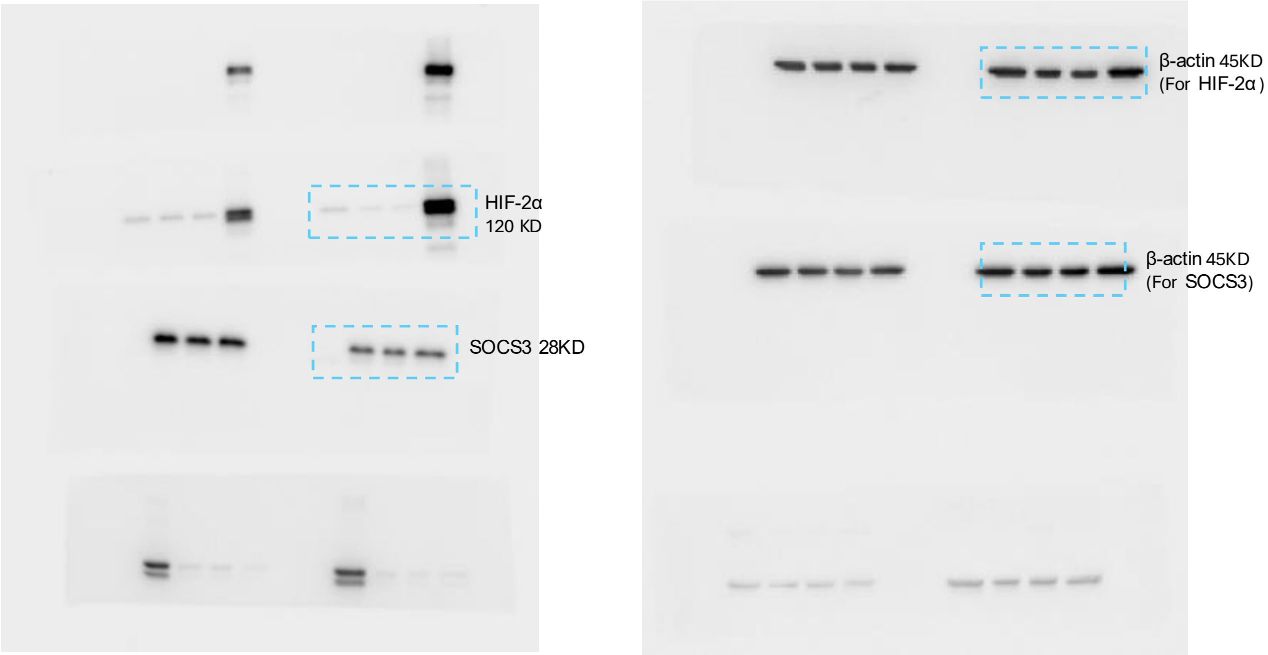

Full unedited blot/gel for Figure 3G

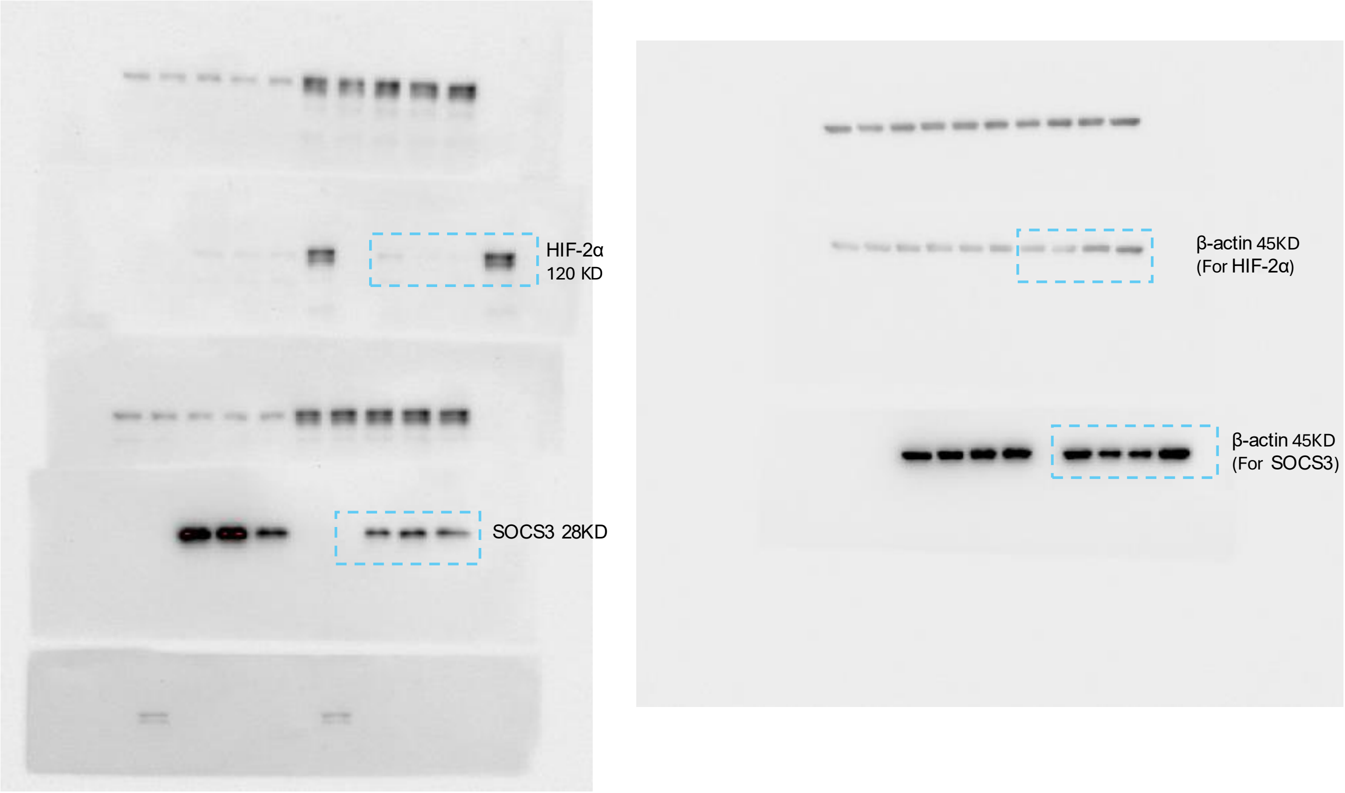

Full unedited blot/gel for Figure 3O

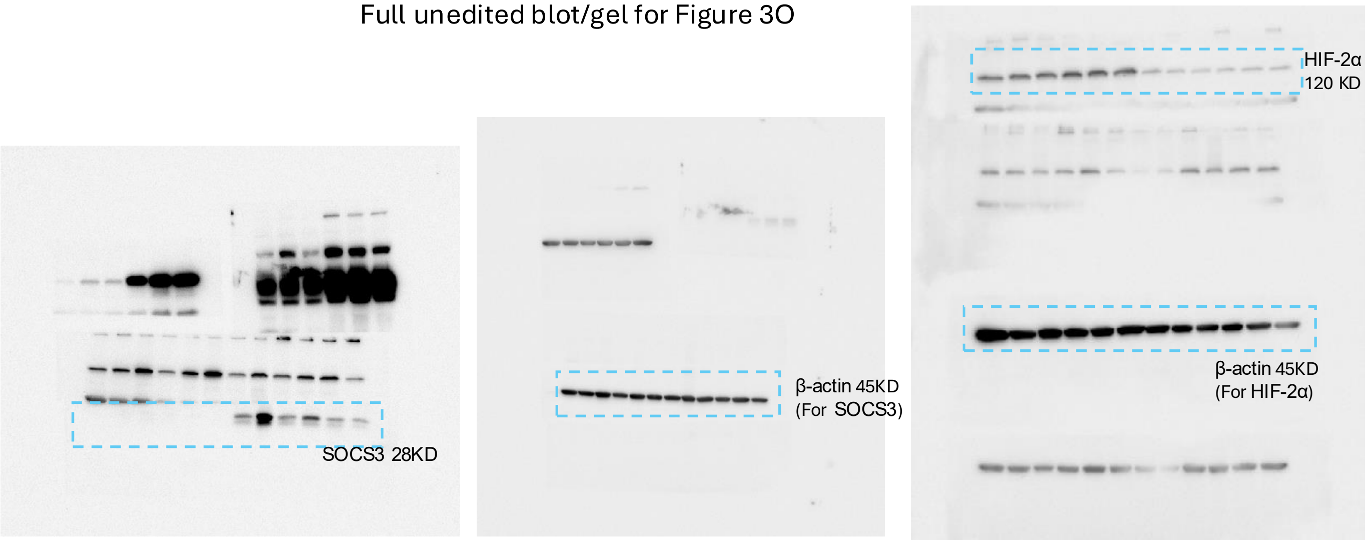

Full unedited blot/gel for Figure 3T

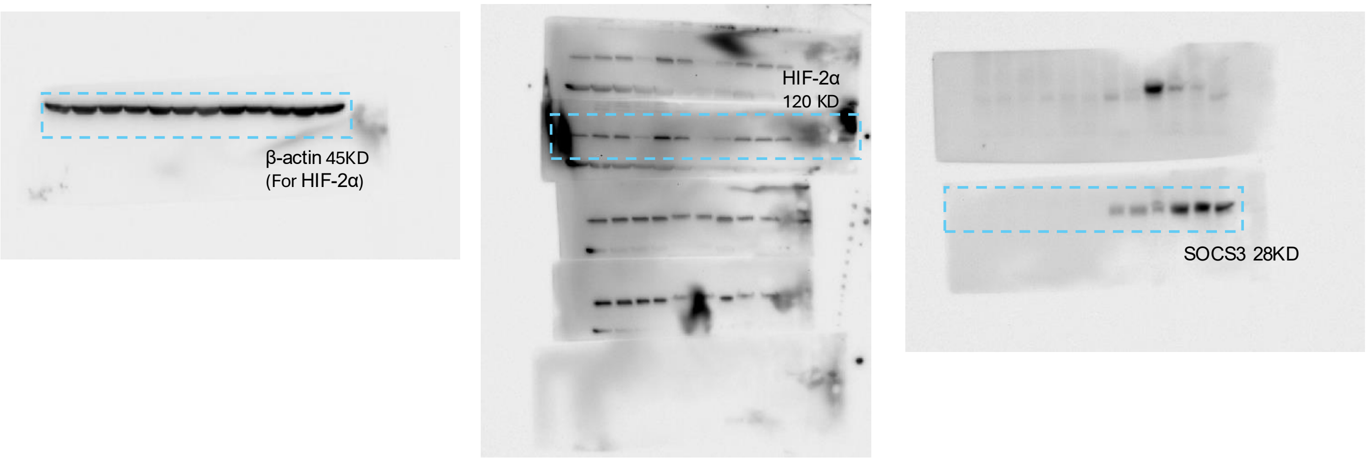

Full unedited blot/gel for Figure S3A

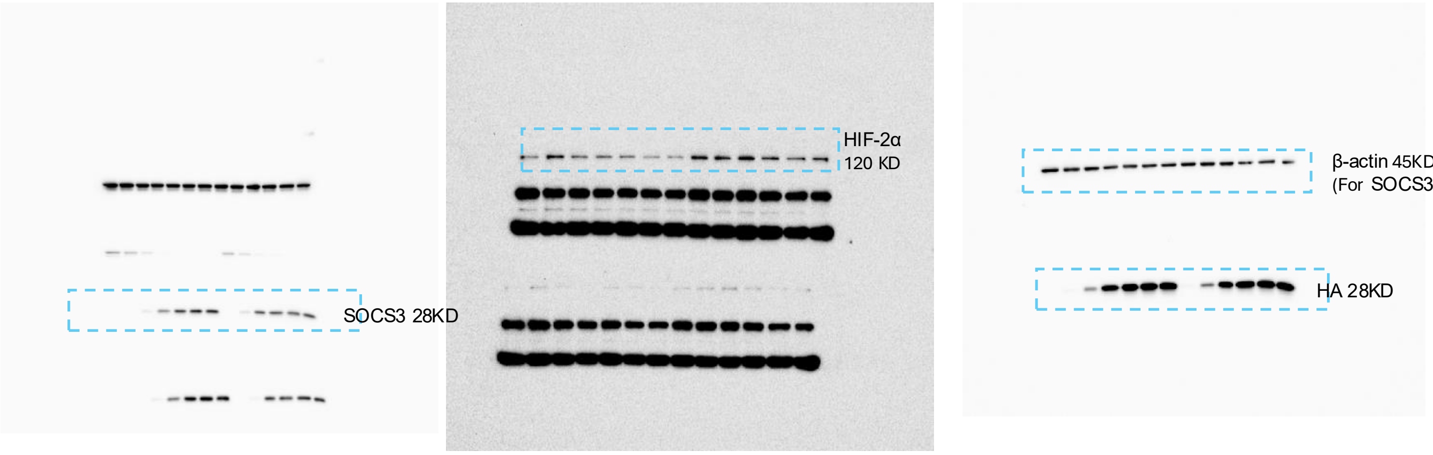

Full unedited blot/gel for Figure S3F

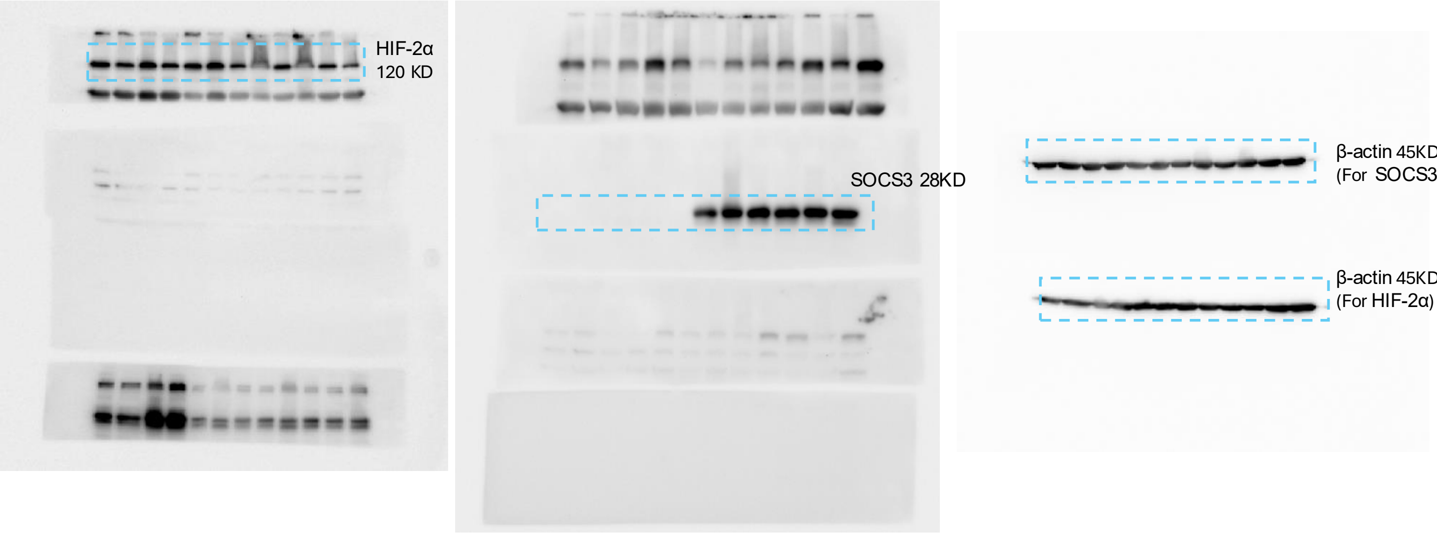

Full unedited blot/gel for Figure 4B

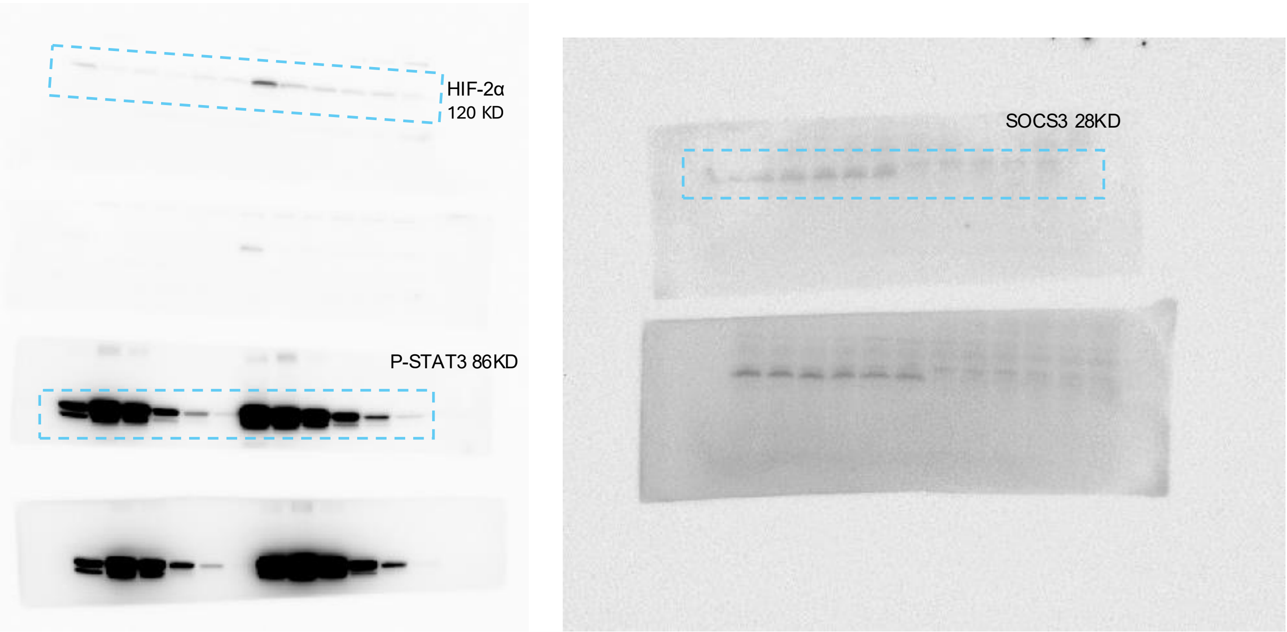

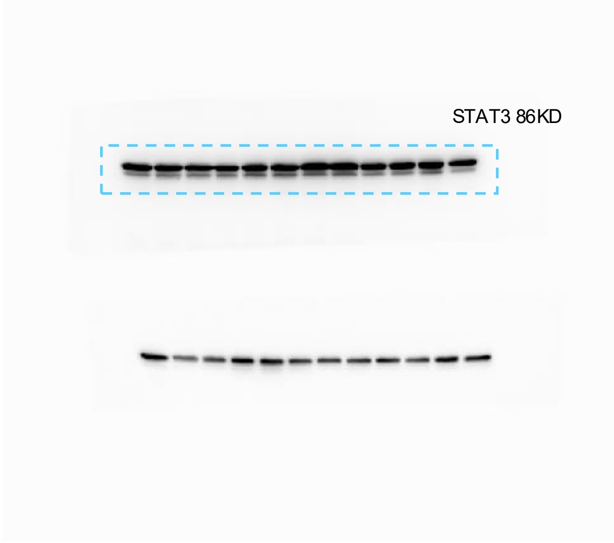

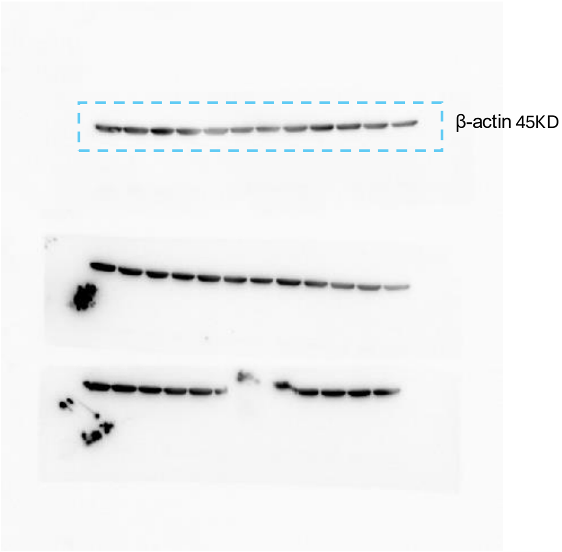

Full unedited blot/gel for Figure 4H

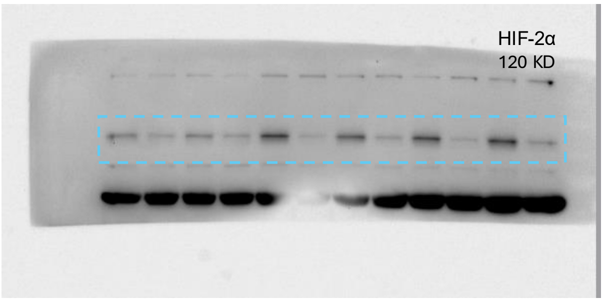

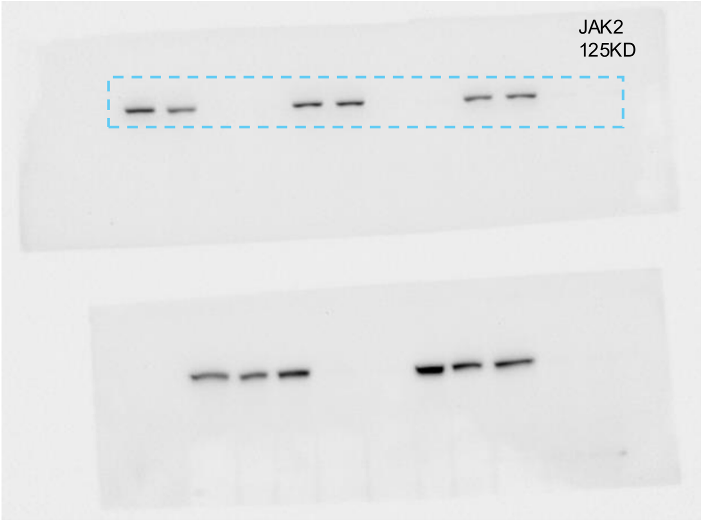

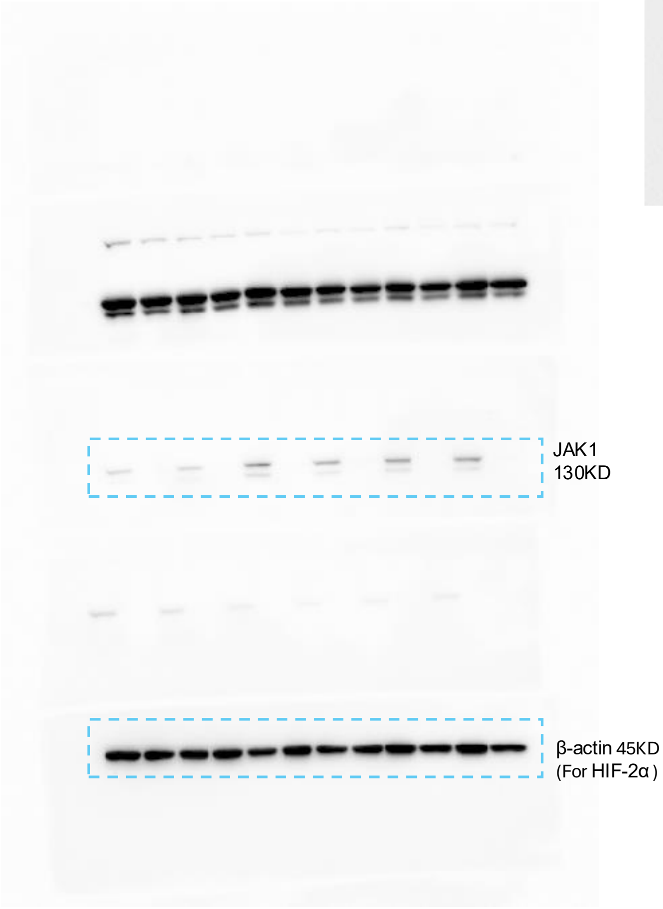

Full unedited blot/gel for Figure 4N

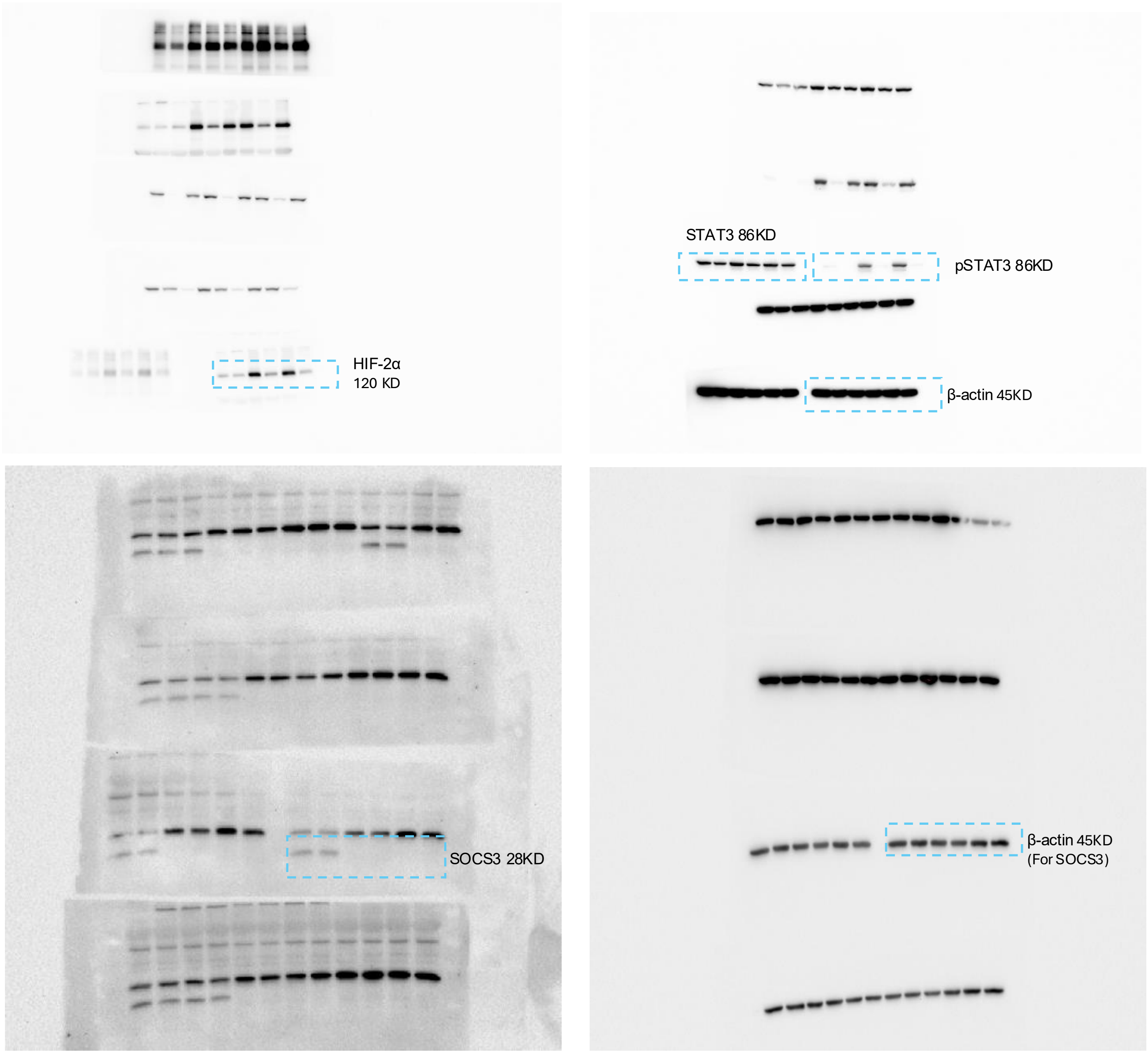

Full unedited blot/gel for Figure 4P

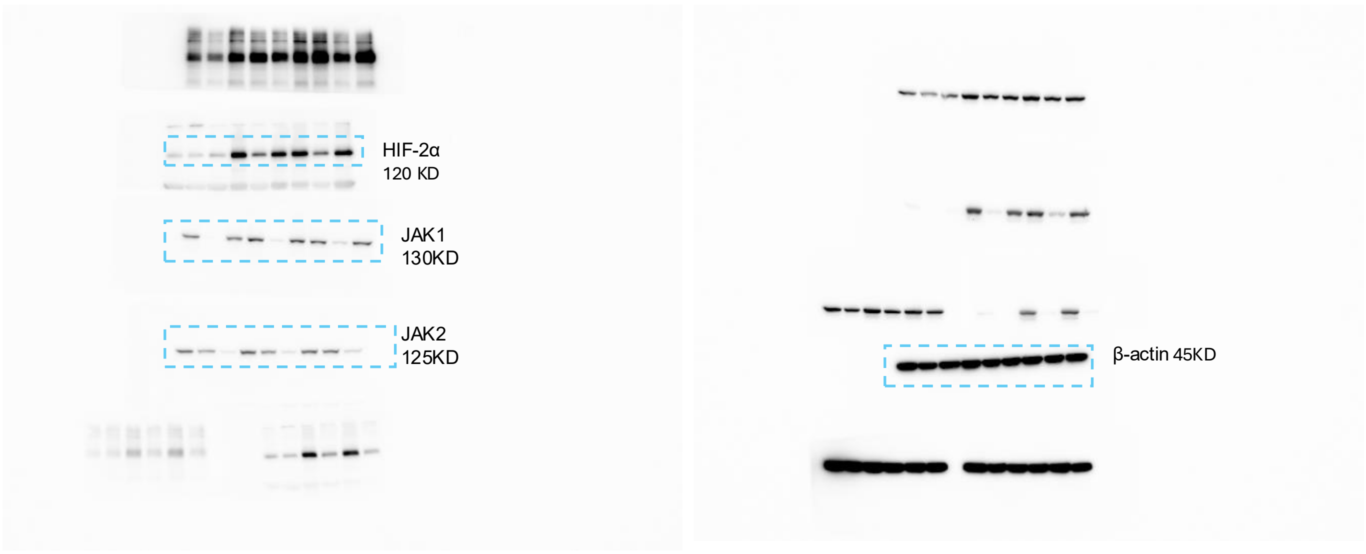

Full unedited blot/gel for Figure 4R

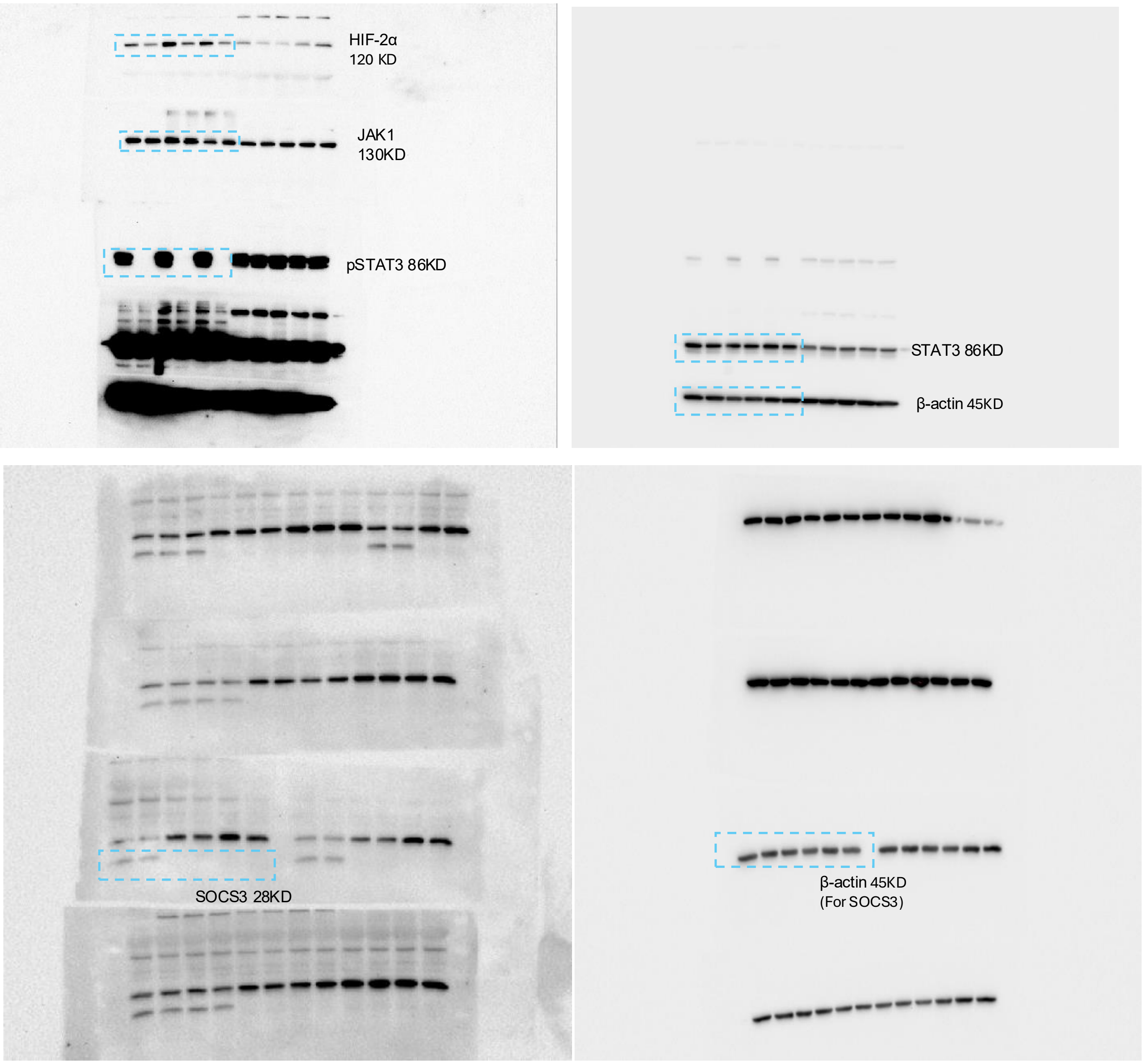

Full unedited blot/gel for Figure 4T

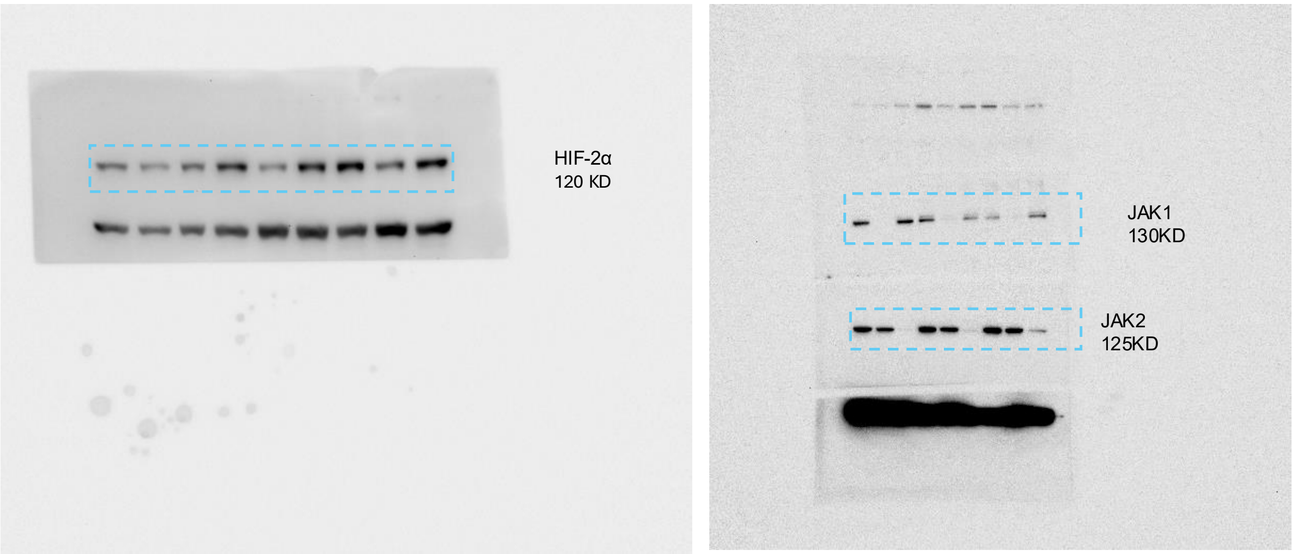

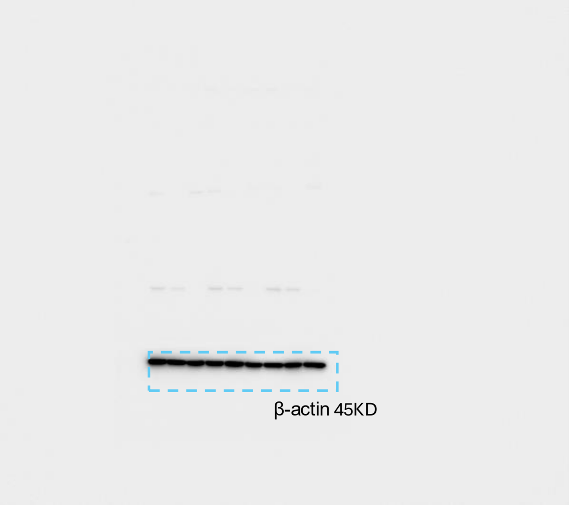

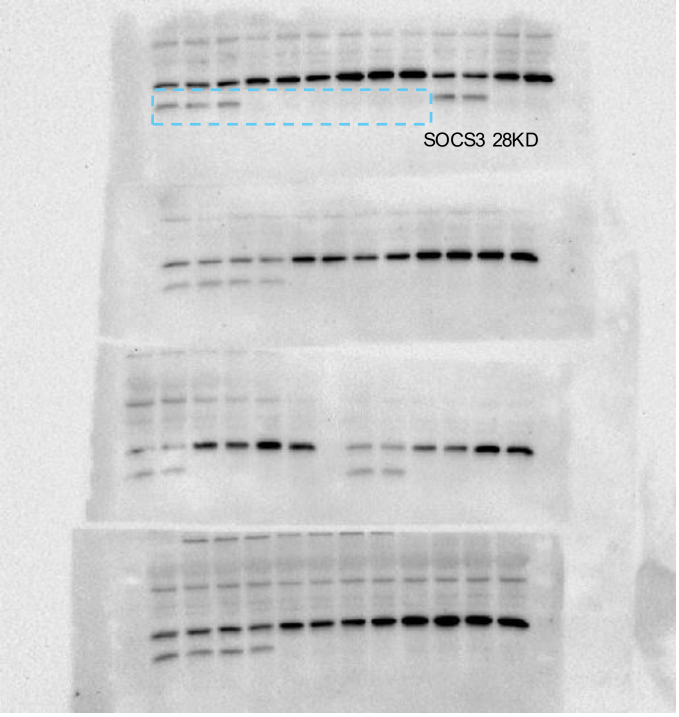

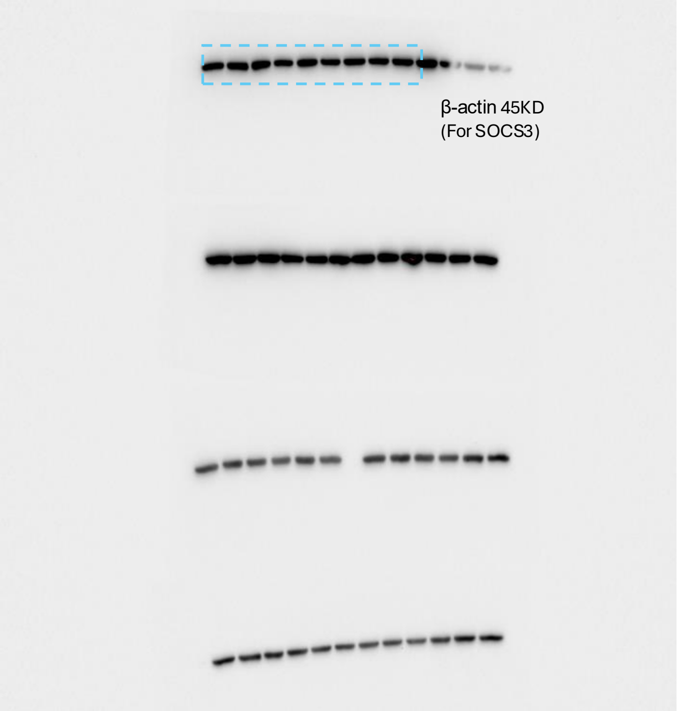

Full unedited blot/gel for Figure S4C

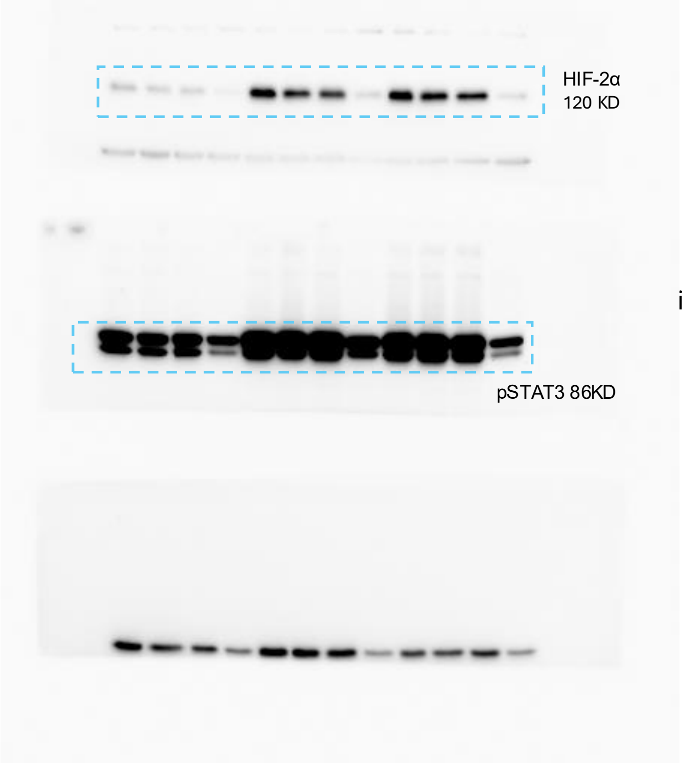

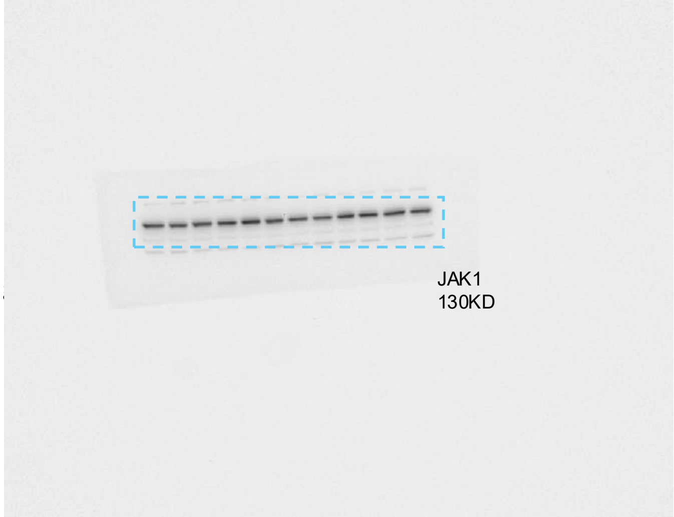

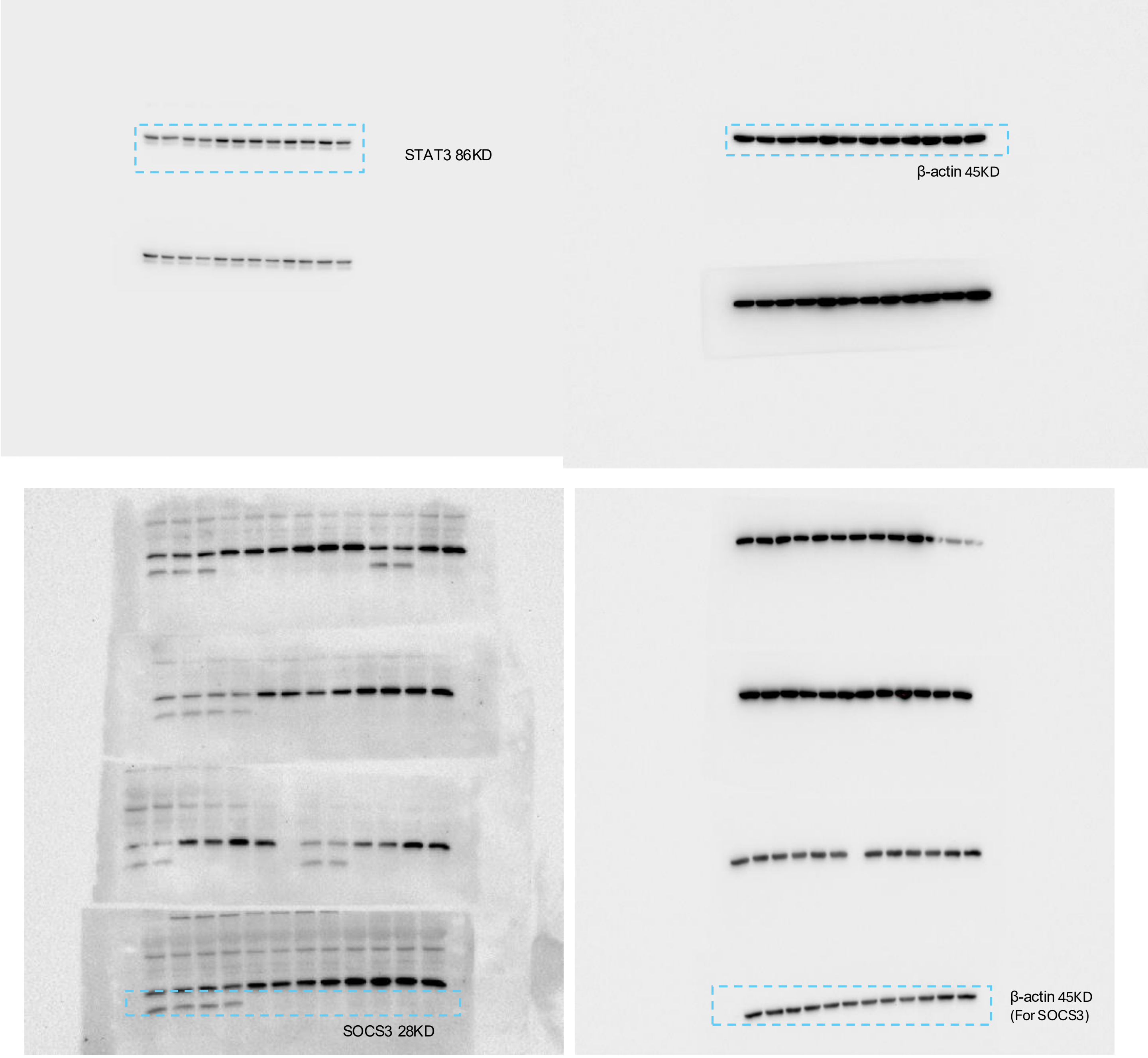

Full unedited blot/gel for Figure S4D

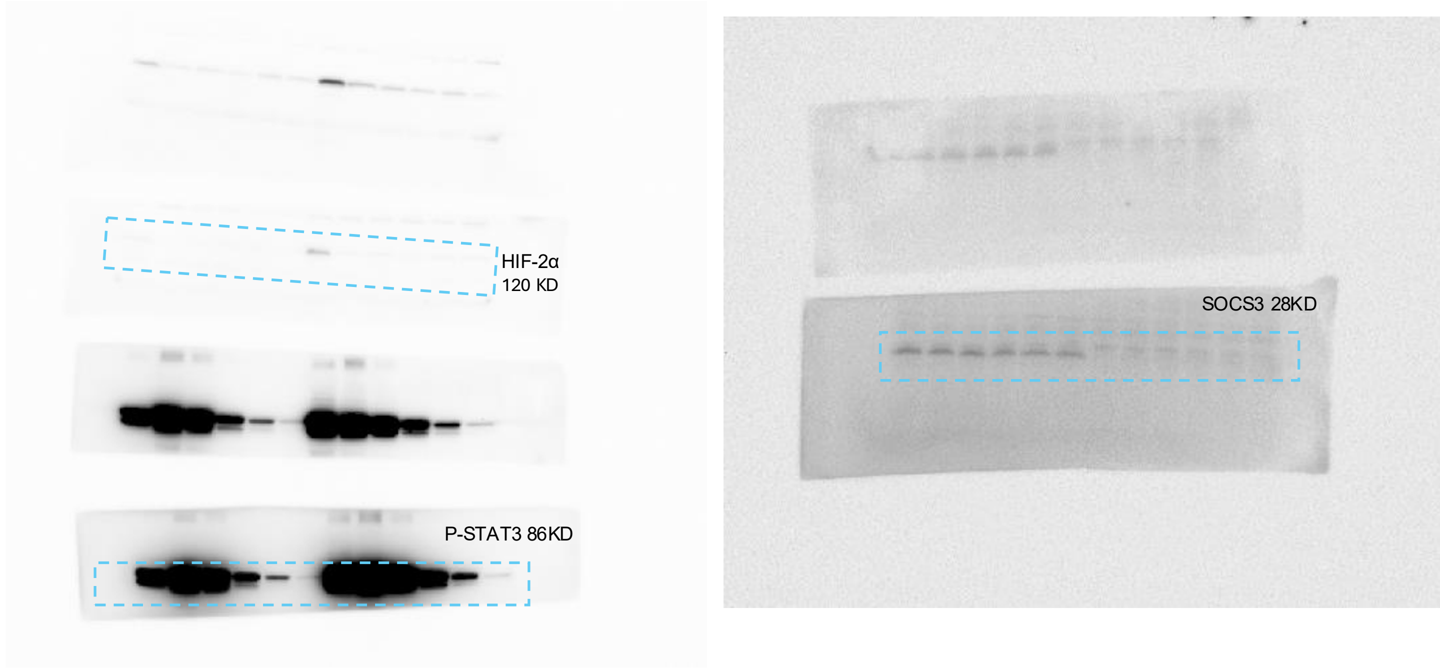

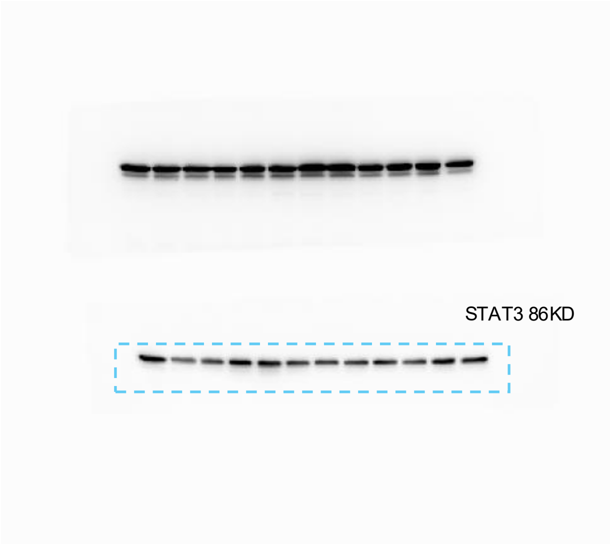

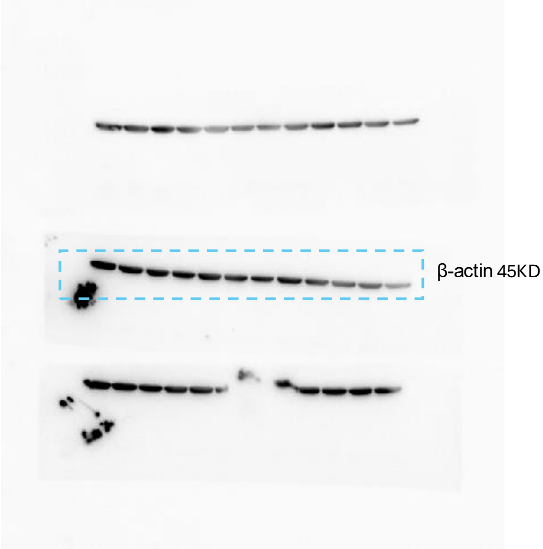

Full unedited blot/gel for Figure S4F

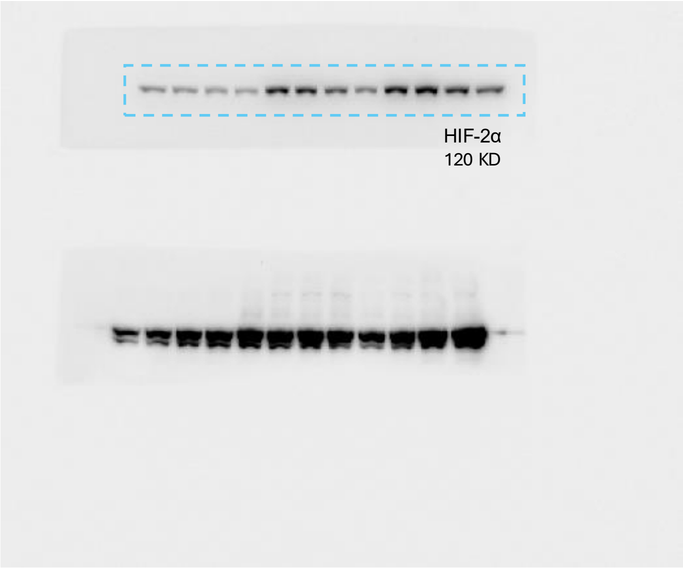

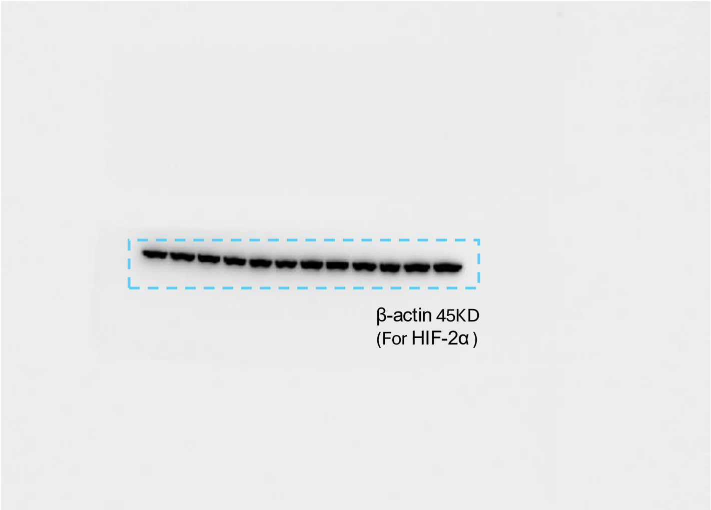

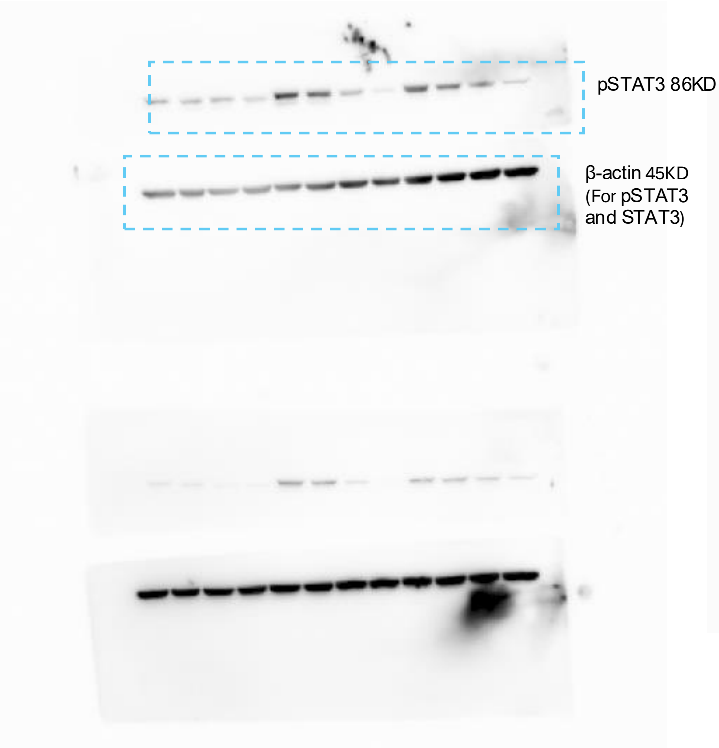

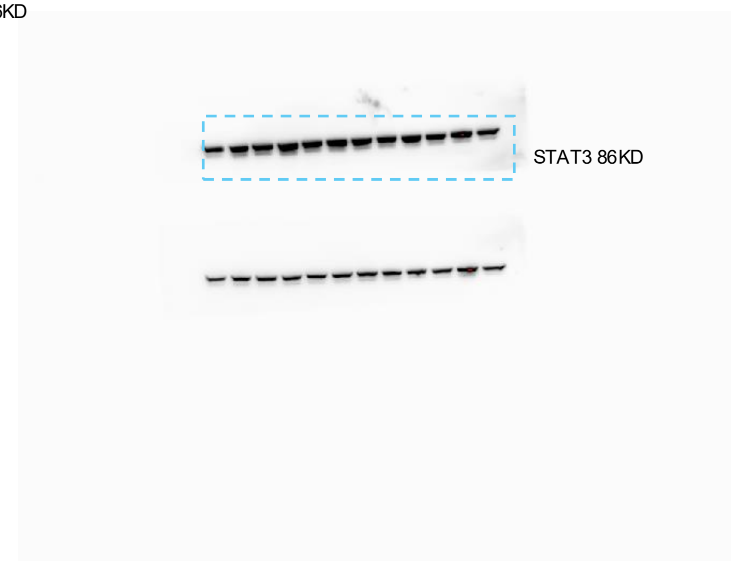

Full unedited blot/gel for Figure S4G

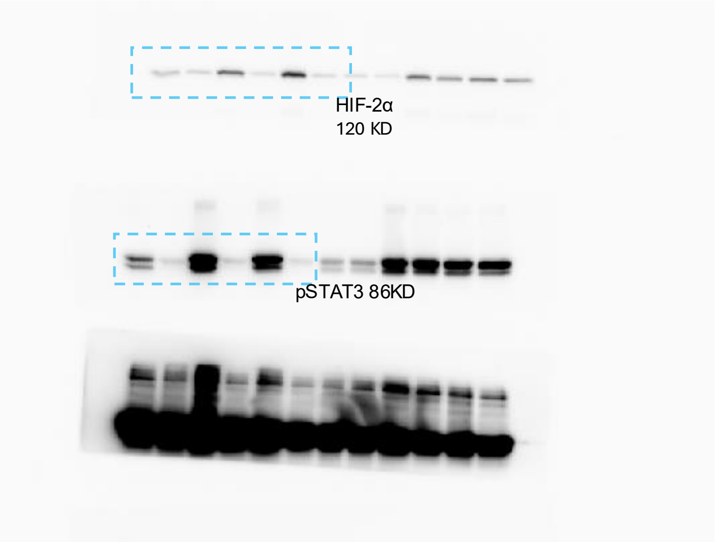

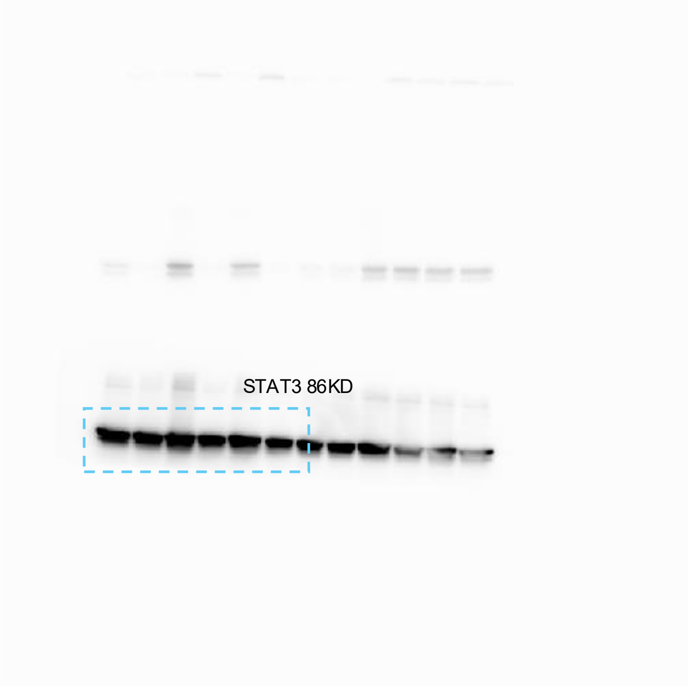

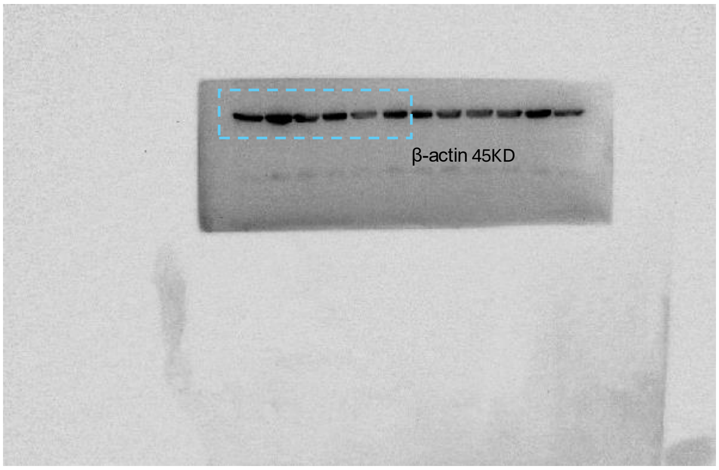

Full unedited blot/gel for Figure 5B

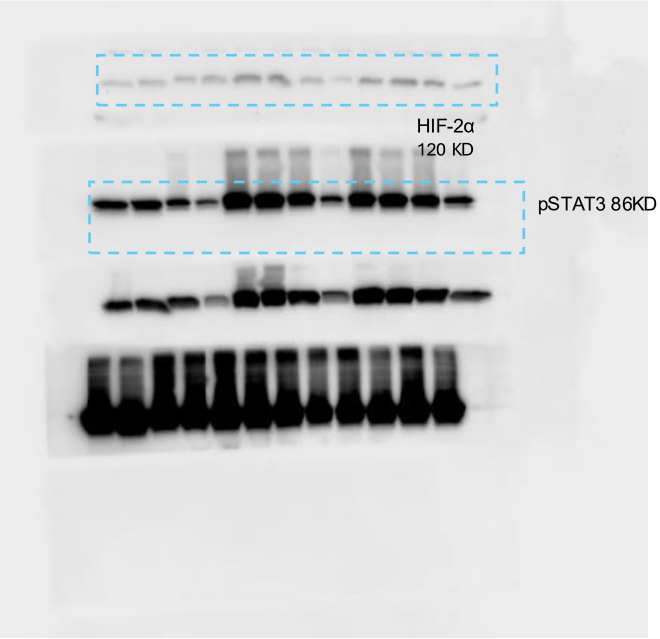

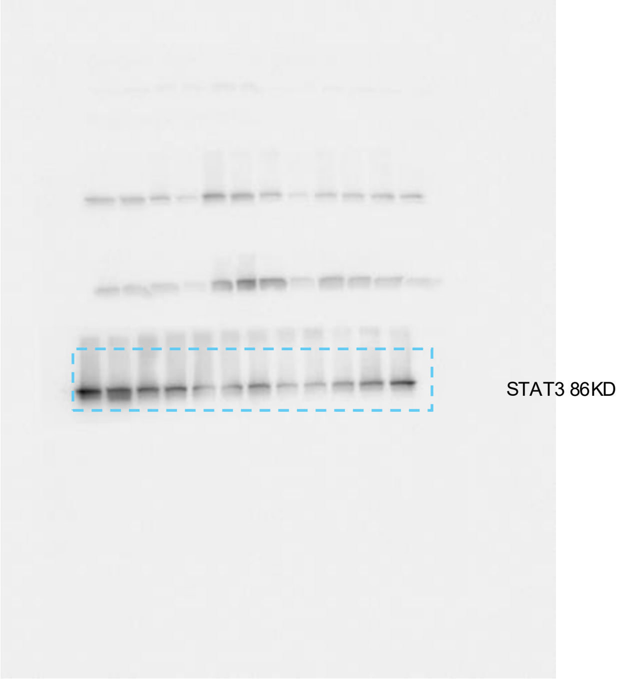

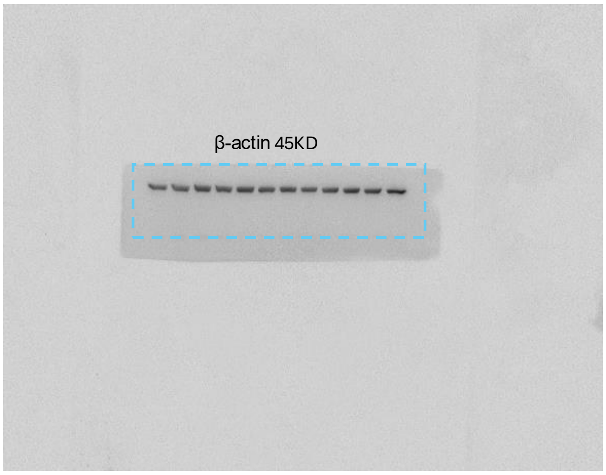

Full unedited blot/gel for Figure 5G

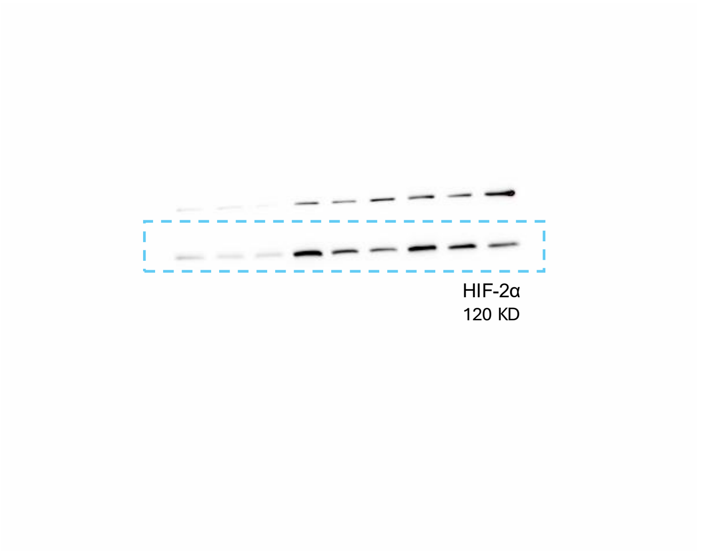

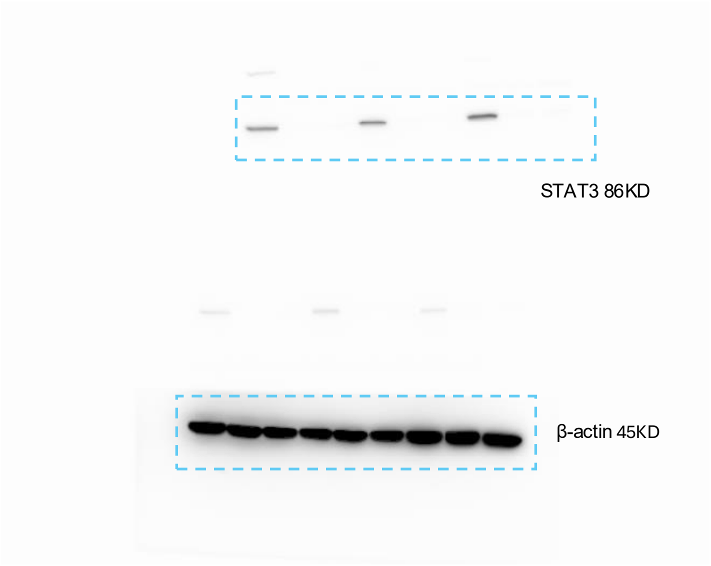

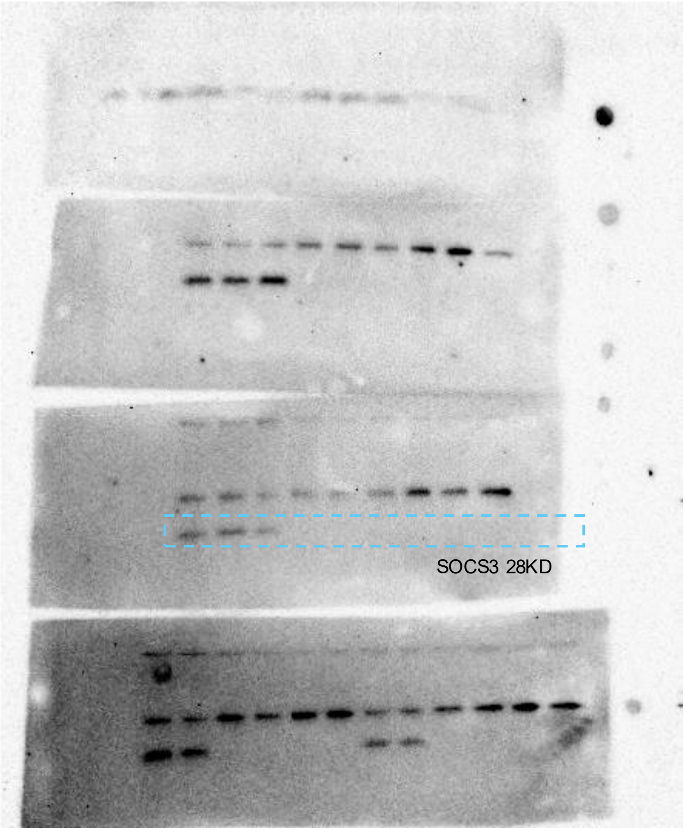

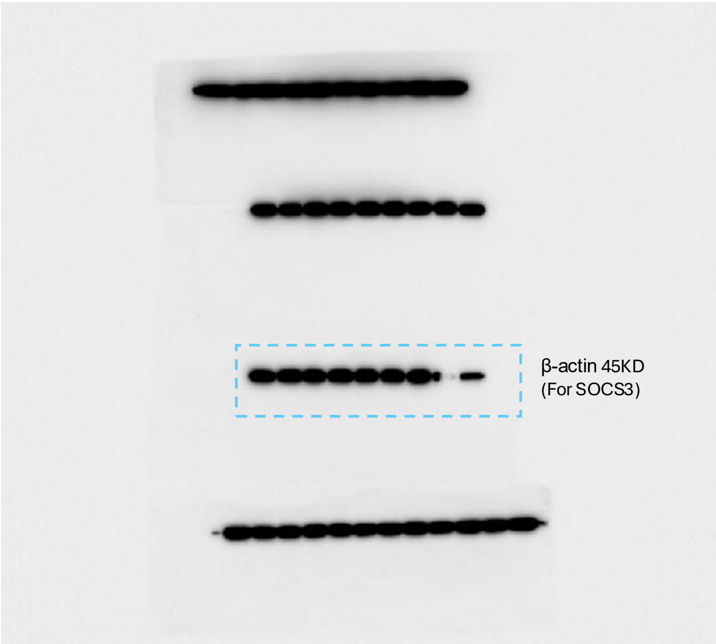

Full unedited blot/gel for Figure 5K

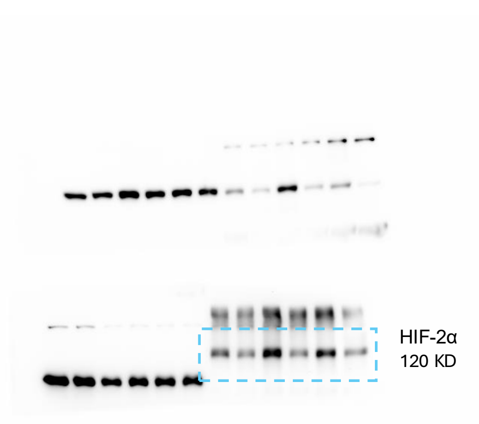

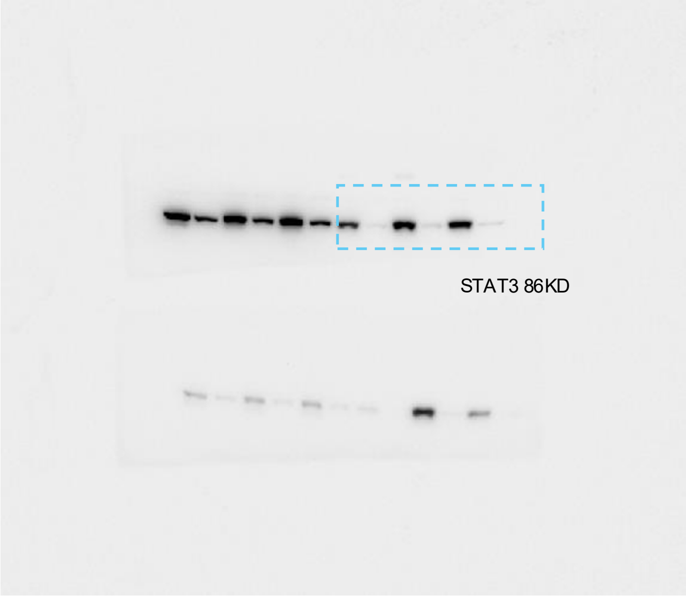

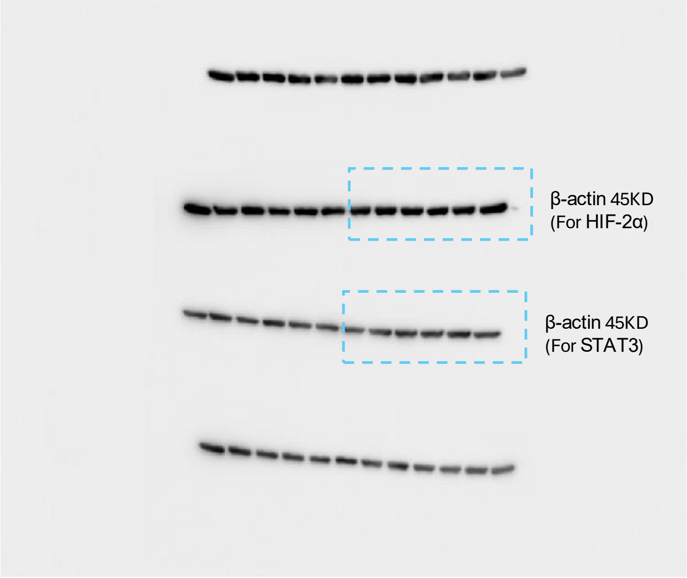

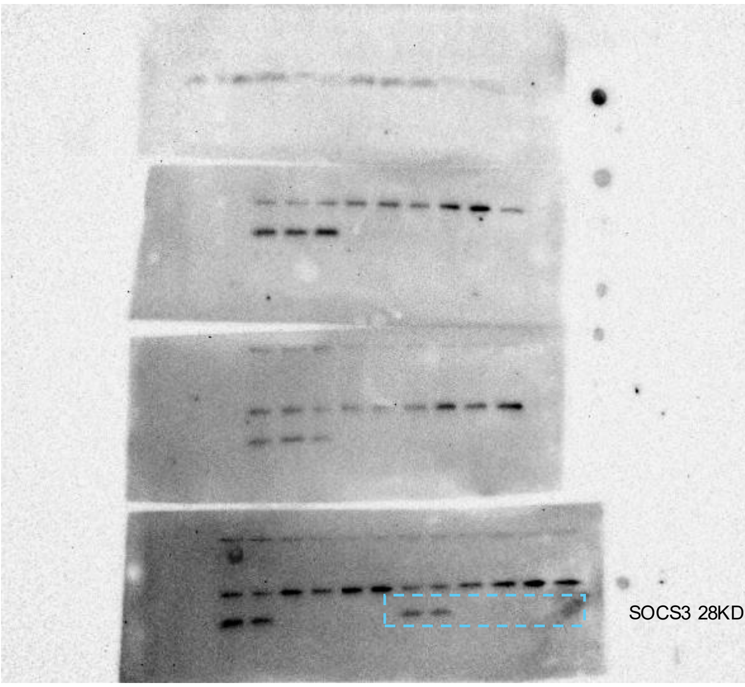

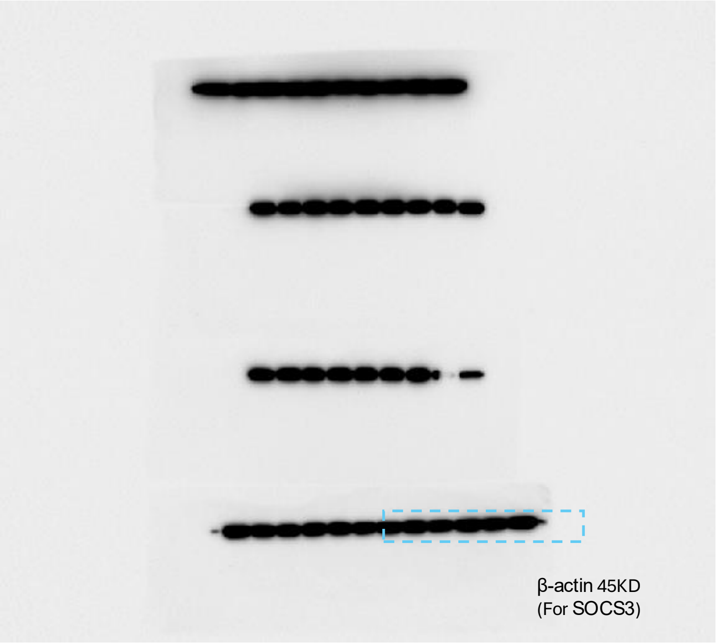

Full unedited blot/gel for Figure 5L

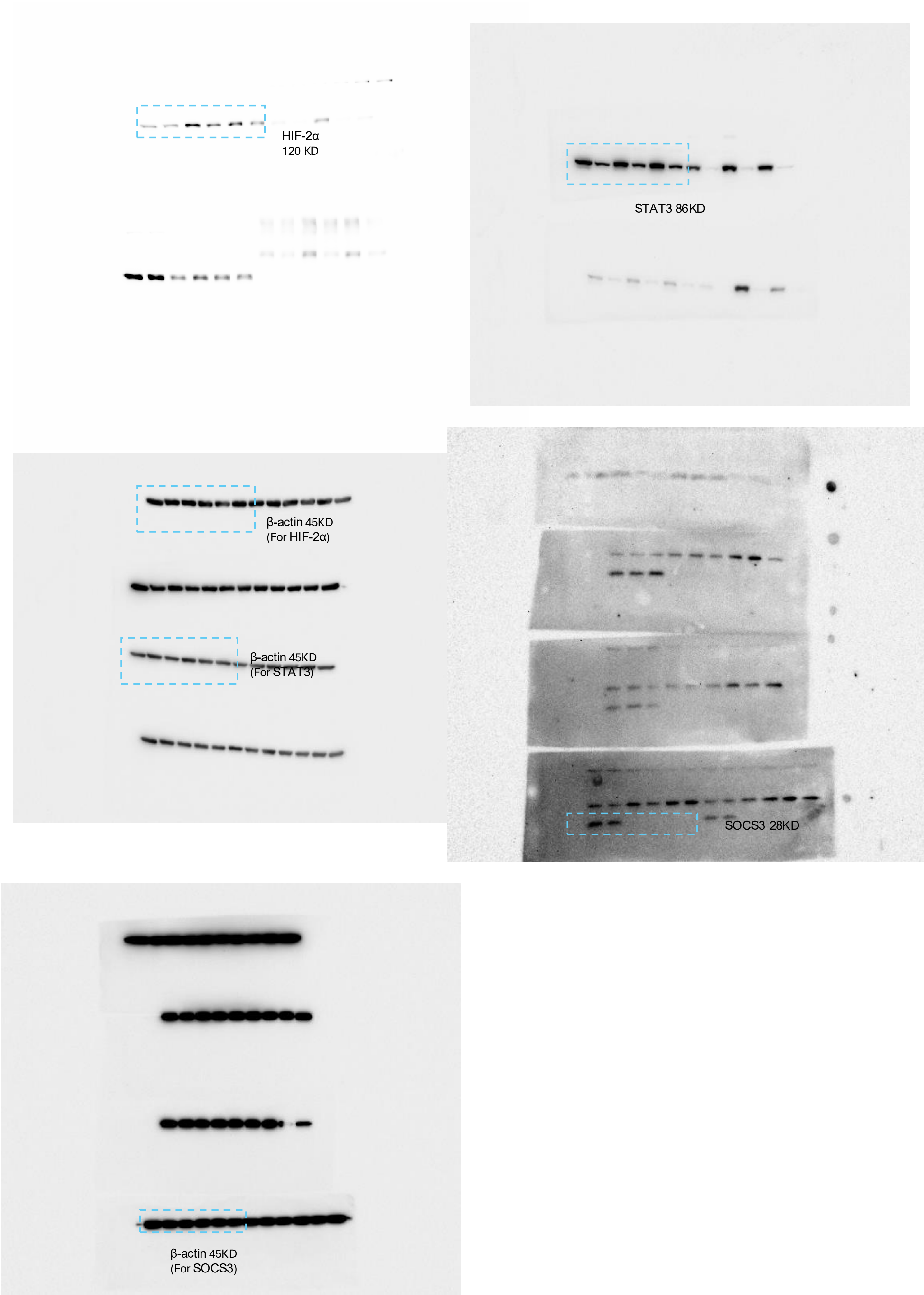

Full unedited blot/gel for Figure 5M

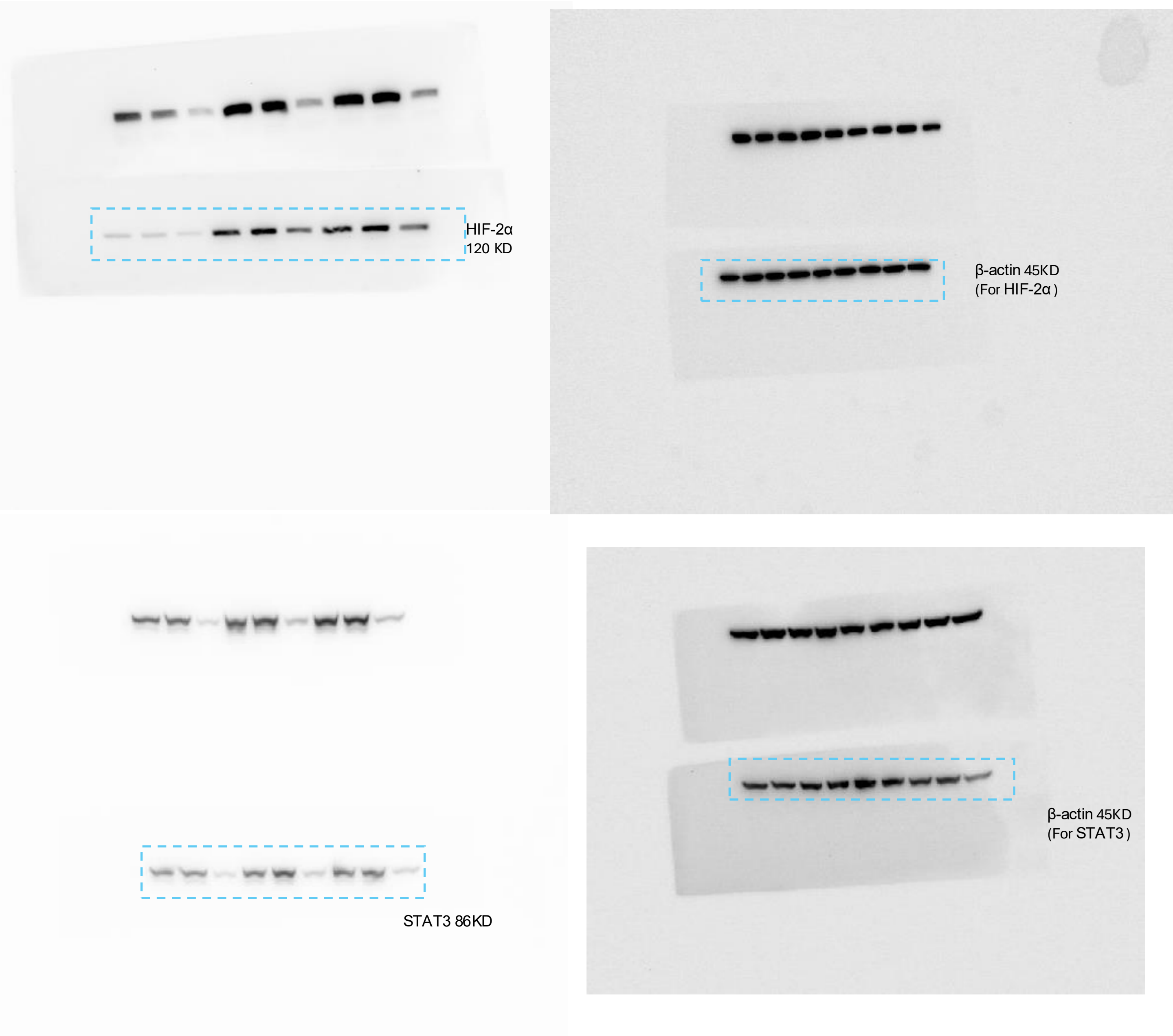

Full unedited blot/gel for Figure 5Q

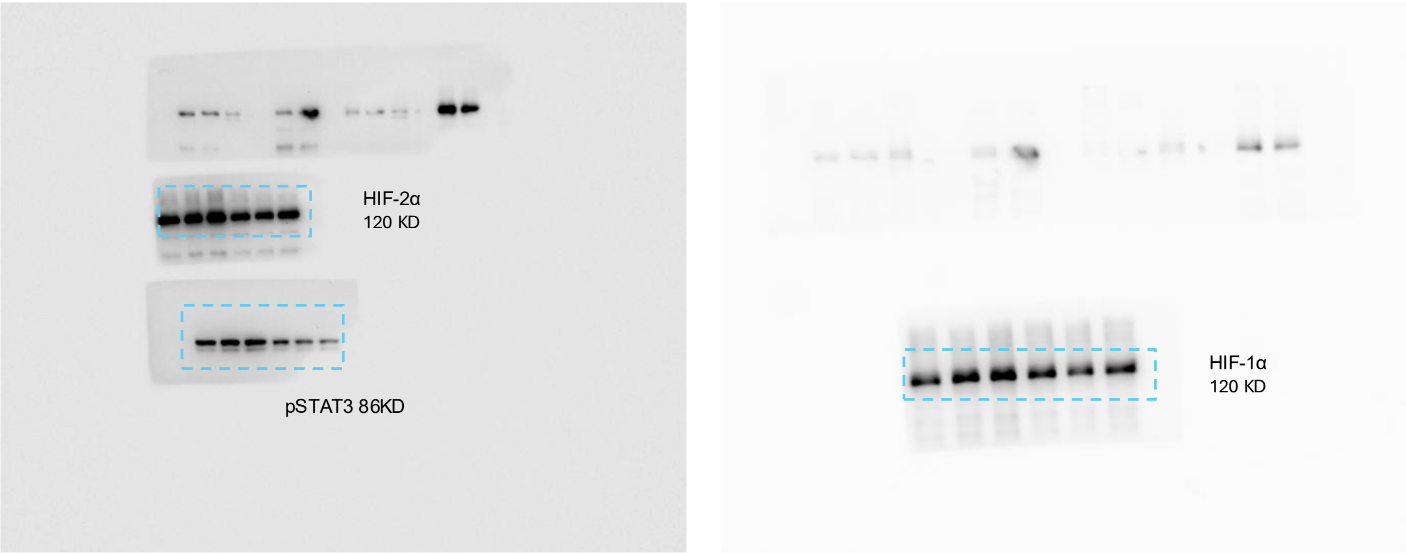

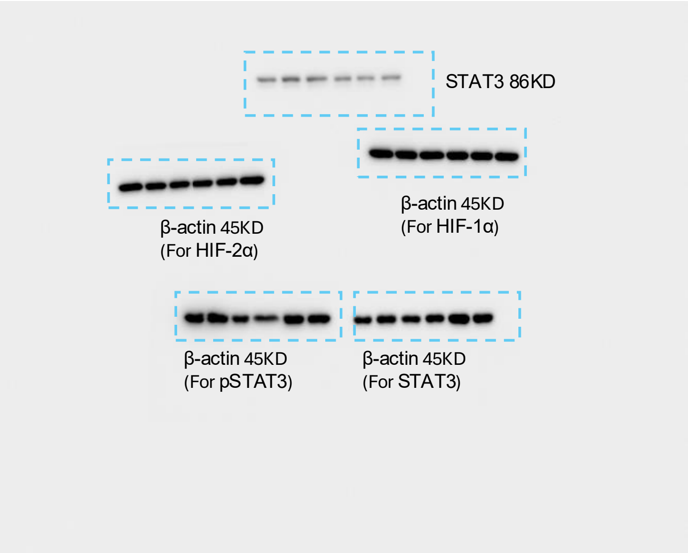

Full unedited blot/gel for Figure S5E

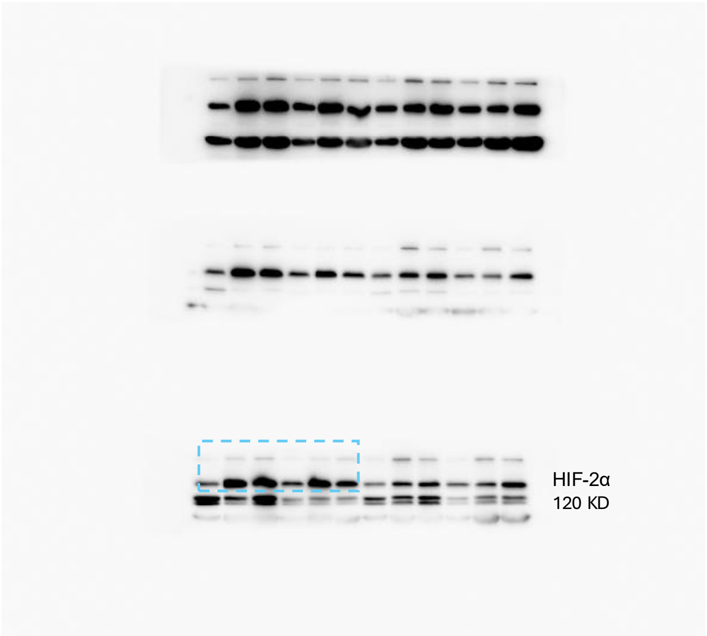

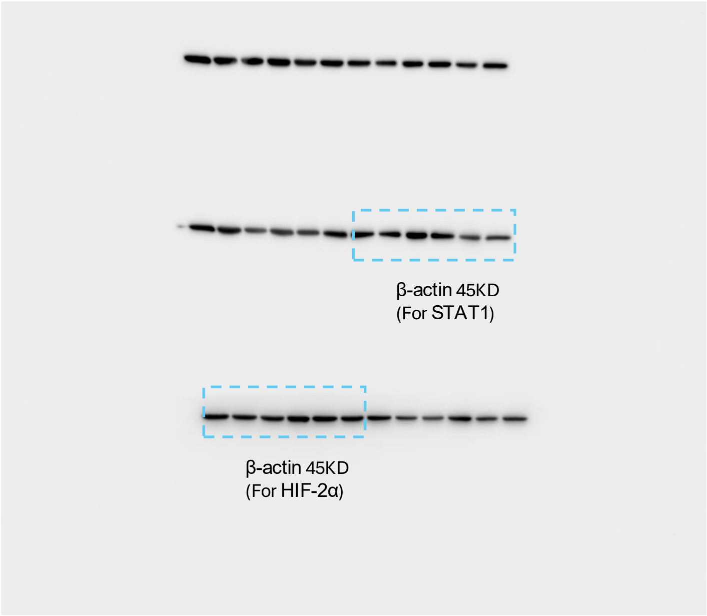

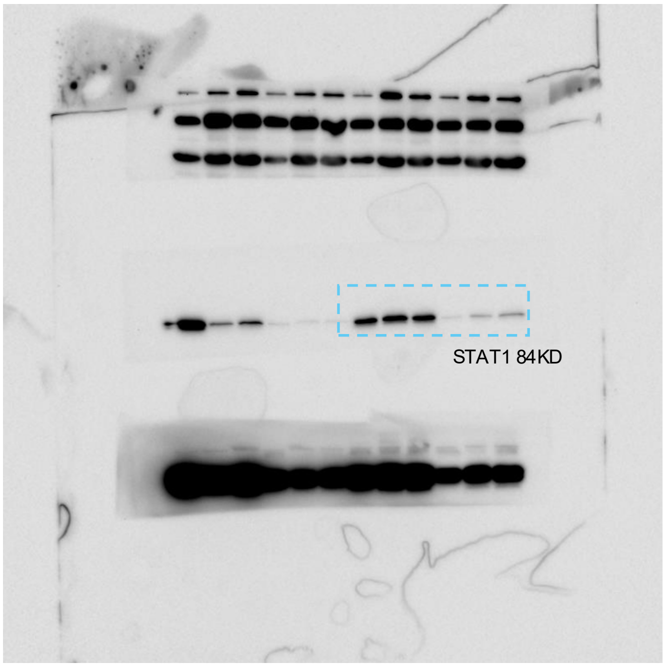

Full unedited blot/gel for Figure S5F

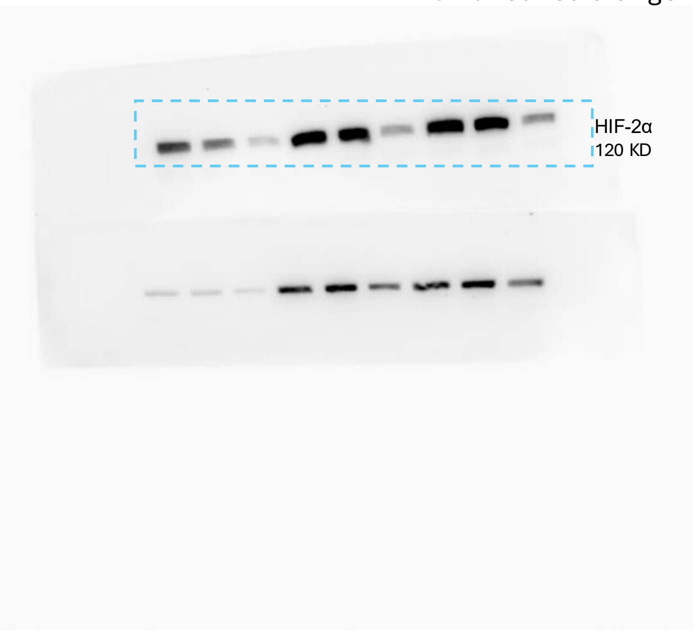

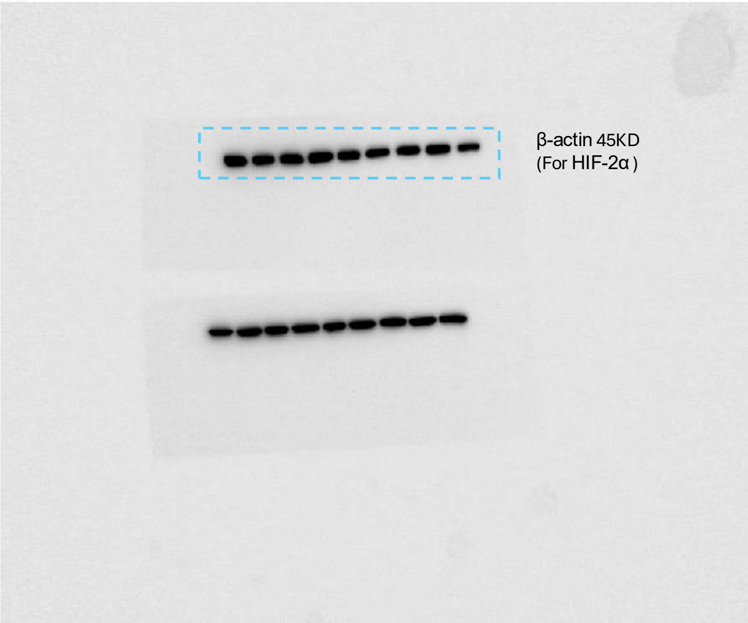

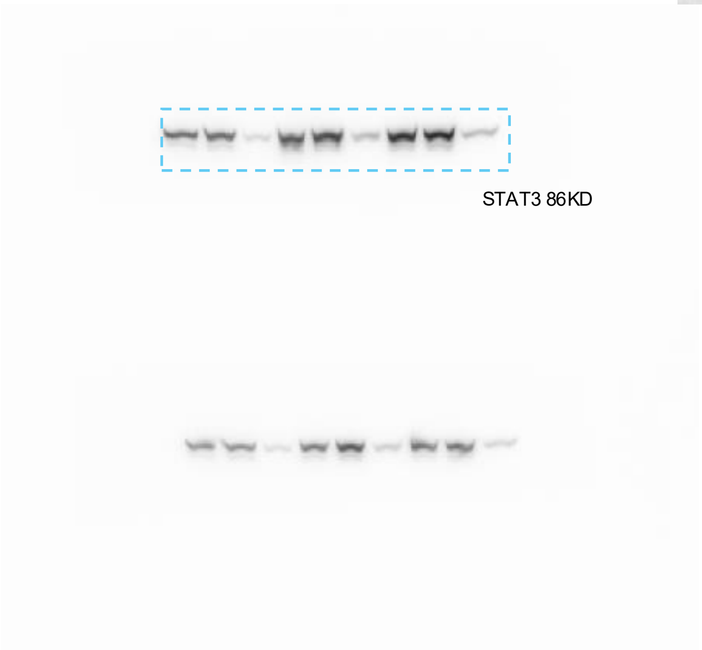

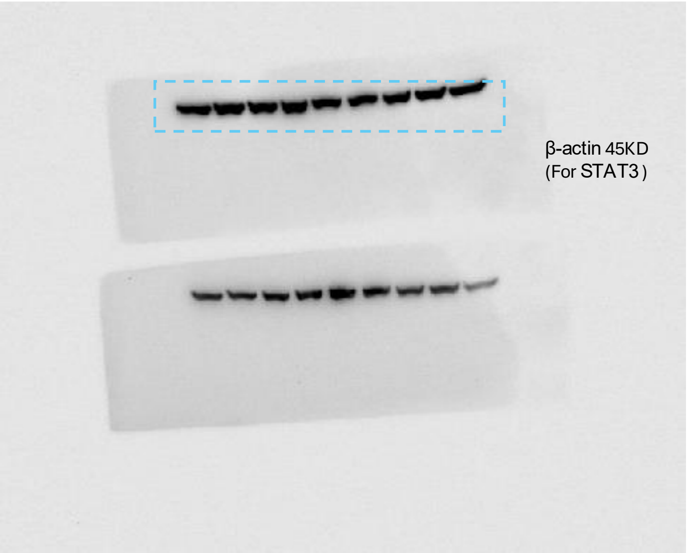

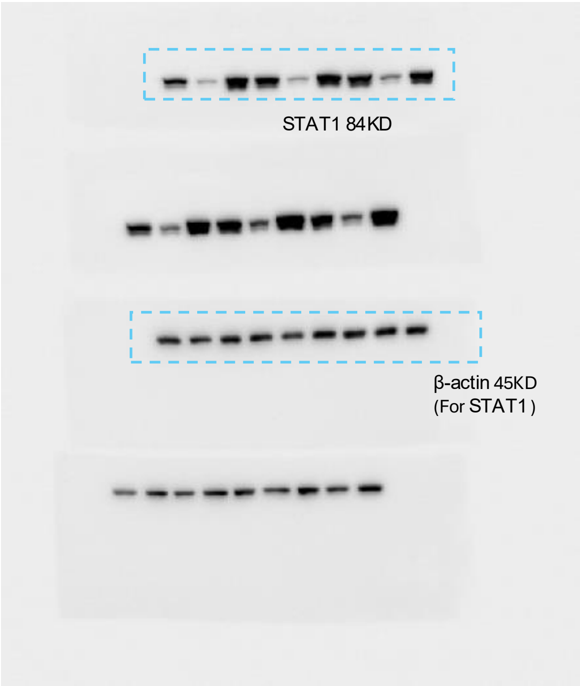

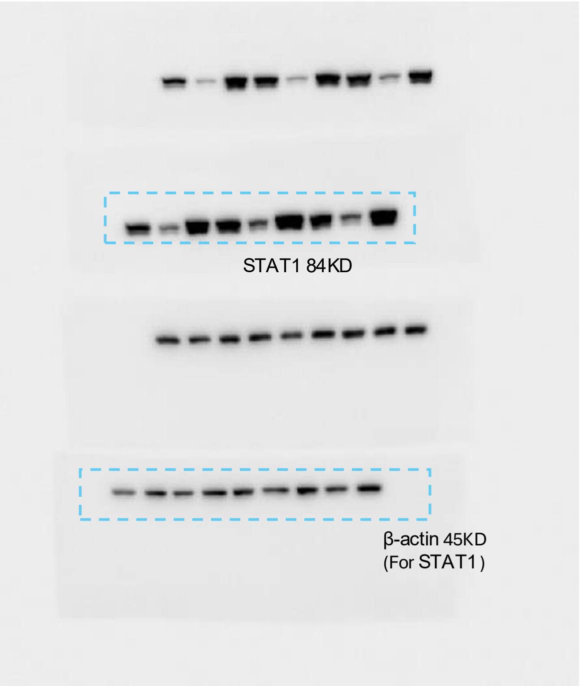

Full unedited blot/gel for Figure S5G

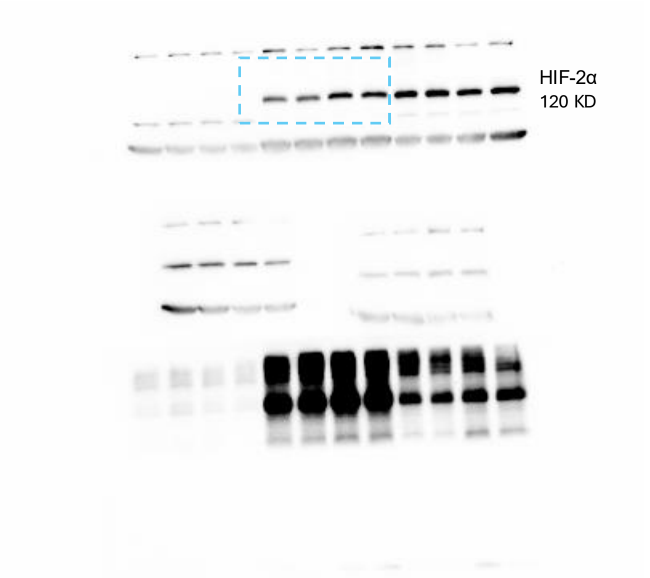

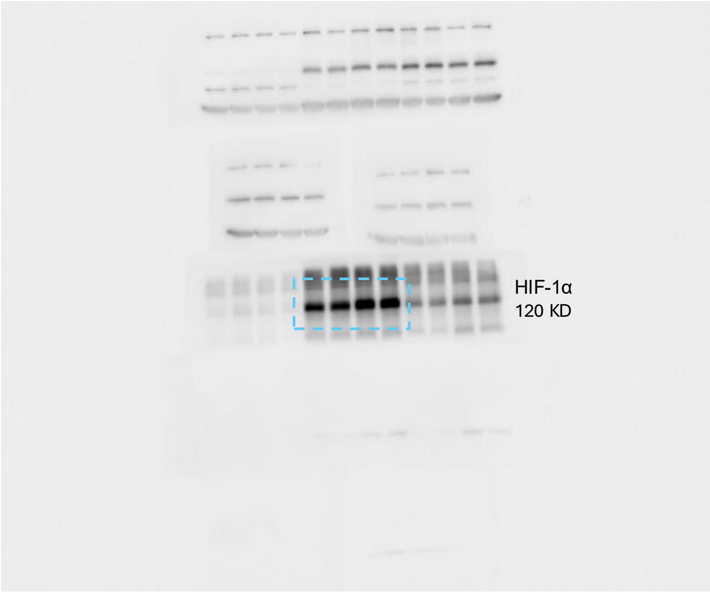

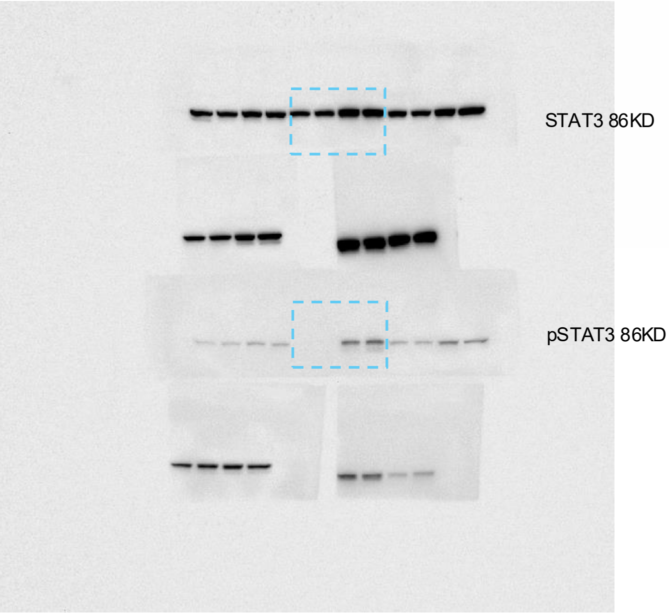

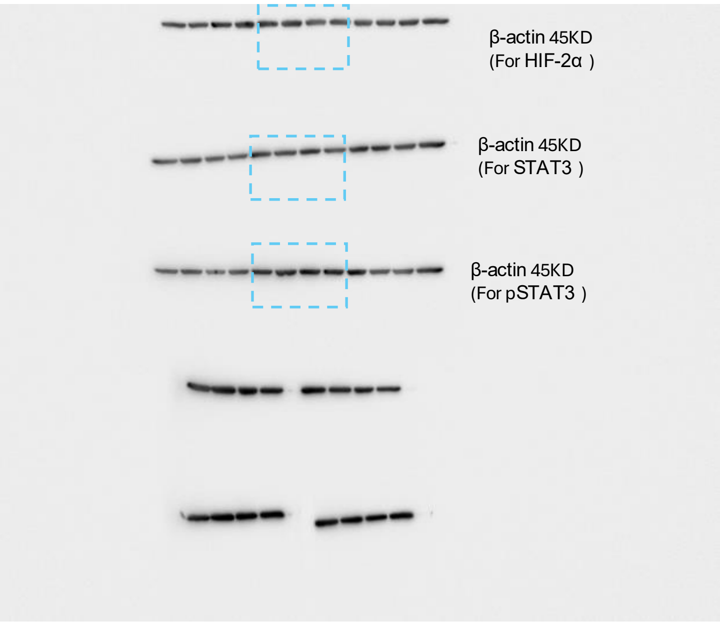

Full unedited blot/gel for Figure 6C

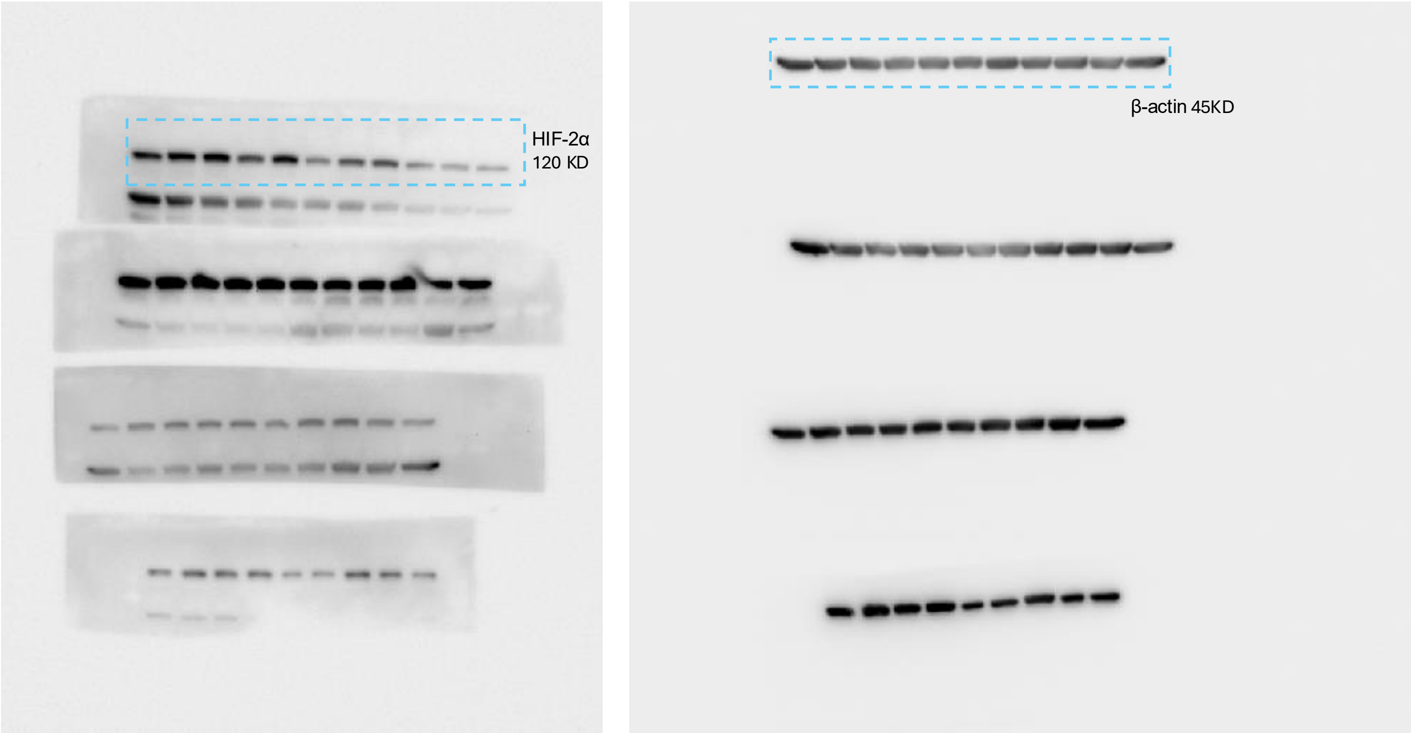

Full unedited blot/gel for Figure 6G

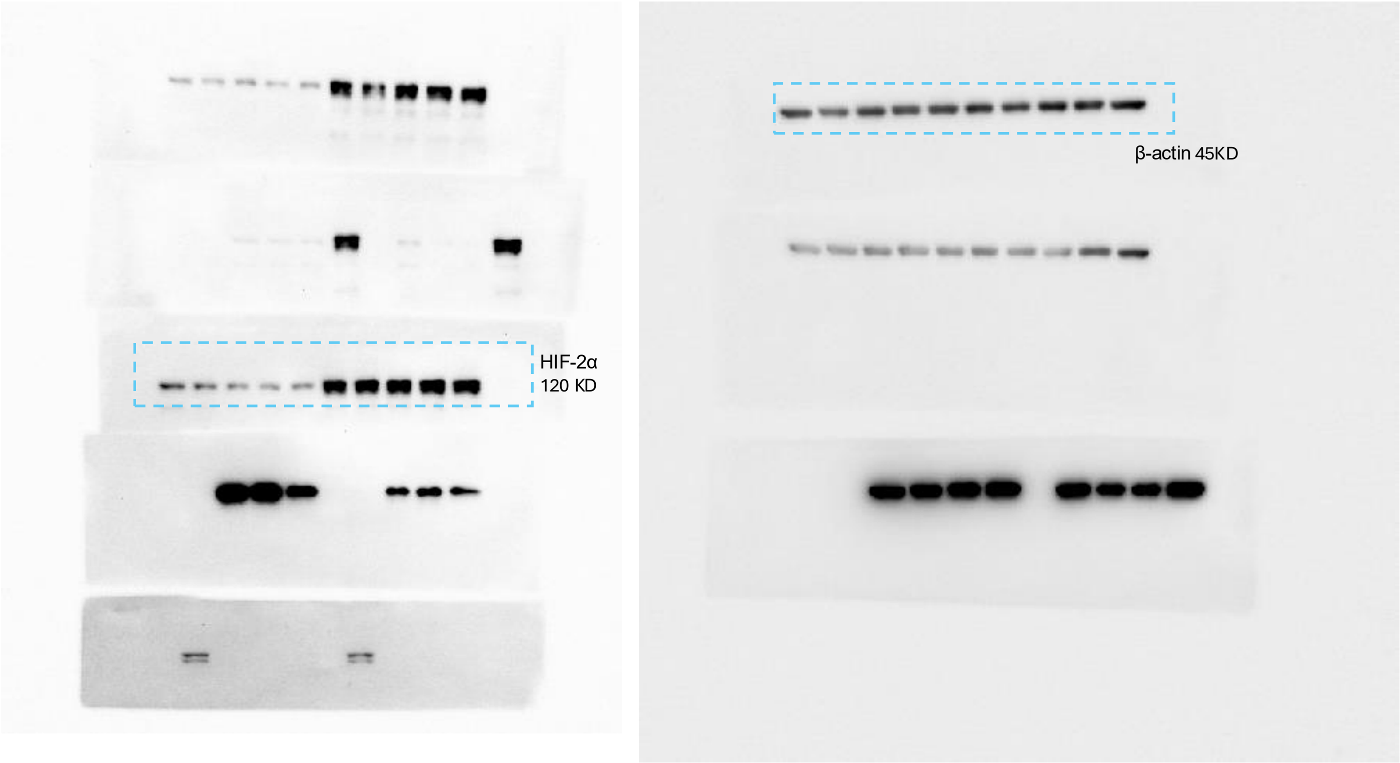

Supplement: Unedited blot and gel images [file jci-136-201639-s008.pdf]
